# A Modular Approach to Tuning Emissive *N*-Quinolyl Through-Space Charge Transfer States Using sp^3^-Scaffolds

**DOI:** 10.1021/acs.jpcb.4c05220

**Published:** 2024-11-04

**Authors:** Joseph O. Watson, Ruth M. Pollard, Mark T. Sims, Marc K. Etherington, Jonathan P. Knowles

**Affiliations:** † Department of Applied Science, Northumbria University, Ellison Place, Newcastle upon Tyne NE1 8ST, U.K.; ‡ Department of Mathematics, Physics and Electrical Engineering, Northumbria University, Ellison Place, Newcastle upon Tyne NE1 8ST, U.K.

## Abstract

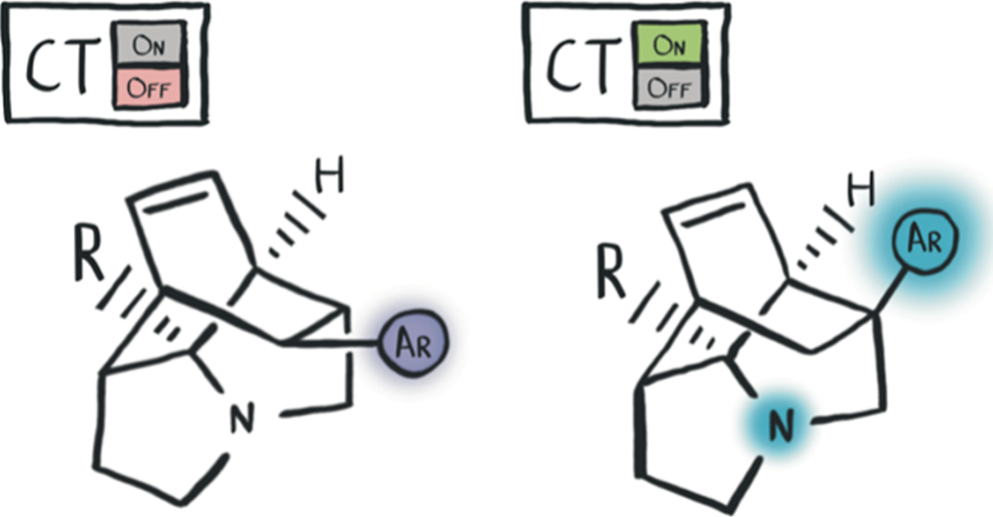

We have shown that palladium-catalyzed cascade processes provide modular access to rigid quinoline-containing tetracyclic amines. This modular approach enables fine-tuning of the through-space charge transfer (TSCT) state formation between the lone pair localized on the nitrogen atom in the cage moiety and the quinoline moiety by variation of both the intramolecular *N*-aryl distance and quinoline substitution. Decreasing this *N*-aryl distance enhances the formation of the TSCT species, giving control over the emission color and photoluminescence quantum yield. Methoxylation of the quinoline unit decreases the propensity of TSCT formation. The development of this structure–activity relationship provides great insight for TSCT formation with an impact on further understanding dimeric, excimeric, and exciplex species. This understanding is crucial for the work underpinning their use in biosensor applications, and the conclusions are of relevance to the broader field of photoluminescence.

## Introduction

**Scheme 1:**
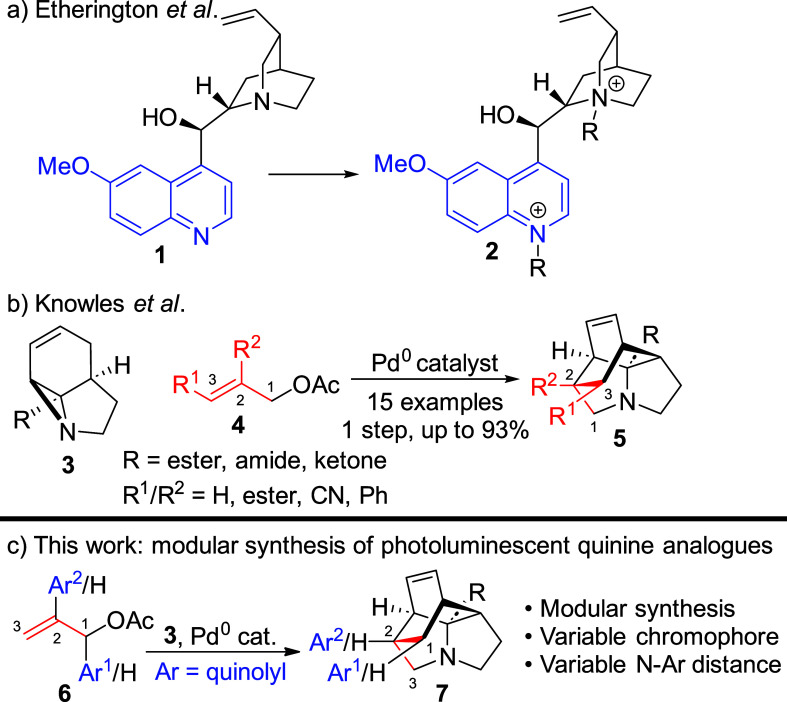
Previous and Current Work toward Photoluminescent Quinolines, including the Naturally Occurring Alkaloid Quinine (**1**) and Syntheses of Tricyclic Scaffolds **5** and **7** via Palladium-Catalyzed Cascade Reactions

Charge transfer (CT) in organic fluorophores is a fundamental photophysical process that can be either beneficial, e.g., facilitating thermally activated delayed fluorescence (TADF),^1,2^ or detrimental, e.g., mediating emission quenching.^3^ It has been previously shown that both protonation^4^ and *N*-alkylation^5,6^ of quinoline-containing compounds allow straightforward synthetic control of CT, emission energies, and photoluminescence quantum yields (PLQYs) (Scheme 1a). The effects of alkylation mirror those of protonation while being inherently more permanent.^5^ Control of photoluminescence and photophysical properties in organic molecules is crucial to a range of applications, from organic light-emitting diodes (OLEDs) to biological imaging and sensing. Given the importance of the CT process in influencing key photophysical parameters, developing structure–activity relationships (SARs) to describe CT state formation will be of interest to chemists, biophysicists, and materials scientists developing new light-emitting materials. CT has historically been considered by two different mechanisms: through-bond (TB) and through-space (TS).^7^ In this work, we establish preliminary SARs to describe through-space charge transfer (TSCT) in systems where the donor is an sp^3^-hybridized nitrogen atom.

Studies on TS interactions are not new,^7−10^ and TSCT is present in a wide range of applications, especially the development of TADF systems.^2,11−14^ However, compared with medicinal chemistry, the development of SARs is not as mature for photophysical studies. There exists some development of SARs for CT in metal–organic frameworks^15,16^ and for photocatalysts,^17,18^ but the emissive nature of CT states and their respective SARs remains less well explored. Recently, there has been increased interest in studying the effect of distance and orientation in sp^3^-linked donor–acceptor systems, in which the link prevents conjugation between the donor and acceptor moieties.^19^ Recent studies have also explored constrained systems involving aryl–aryl CT within mixed cyclophanes.^20^ However, in all cases both donor and acceptor have been extended conjugated systems, creating ambiguity around how the donor–acceptor distance should be measured.^21^ This can be further complicated by issues of orientation relating to the nature of the junction, for example, in the case of spiro-fused compounds.^22^ Systems involving a single, localized lone pair as the donor would thus offer simplification of the measurement of the distance with respect to the donor. Further, since protonation and *N*-alkylation have a significant and predictable effect on CT states in quinoline-containing compounds,^5^ such systems would also provide an ideal starting point for exploring these vital fundamental processes and developing effective SARs.

One prominent set of quinoline-containing compounds are the cinchona alkaloids, particularly the historically important antimalarial quinine. However, both the chromophore and overall architecture of quinine are relatively inflexible due to its isolation from a natural source, limiting the application of such species in the development of SARs. Further, while methods for the synthesis of cinchona alkaloids exist,^23,24^ they are often insufficiently modular to permit significant structural variation. We recently reported that photochemically accessed tricyclic aziridines^25^ undergo efficient Pd-catalyzed cascade processes to provide cage structures **5** (Scheme 1b),^26^ which, while initially lacking a quinoline chromophore, appeared to offer similar rigidity, functionality, and *N*-aryl distances as those observed in quinine. Importantly, the modular nature of this synthesis enables facile interchange of the components employed within it. We therefore considered whether this system could be used as a quinine surrogate, enabling the alteration of both the aryl chromophore and *N*-aryl distance within compound **7**. This would enable elucidation of the fundamental processes of TSCT between the cage nitrogen lone pair and quinoline unit,^5,27,28^ creating a basic SAR around these parameters. *N*-Aryl distance in particular is a crucial parameter as the aliphatic cage nitrogen lone pair acts as the electron donor (D), while the quinoline acts as the electron acceptor (A).^29−31^ Given that the D and A units are not conjugated, the CT process necessarily happens TS, giving species with TSCT character. This has an impact on the color of emission and PLQY depending on the solvent environment but also adds to the knowledge surrounding the photophysics of dimeric, excimeric, and exciplex systems. An understanding of the fundamentals of intramolecular and intermolecular CT is crucial, for instance, in the development of new biosensors and ultimately in the general refinement of light-emitting materials including OLEDs.^32^

## Results and Discussion

Quinoline-containing tethers **8a**–**c** were prepared straightforwardly through standard heterocyclic synthesis, while access to system **9** was provided by a modification of the methodology recently reported by Kutsumura et al.^33^ A full discussion of these syntheses is available in the Supporting Information. Both sets of tethers were reacted with tricyclic aziridines **3a**–**c** under the conditions developed previously. While yields proved somewhat variable, it can be seen from Table 1 that the reaction was successful in all cases, yielding a library of 12 compounds possessing variations of both *N*-aryl distance and quinoline identity. A full discussion of the factors affecting this reaction is again provided within the Supporting Information.

**Table 1:**
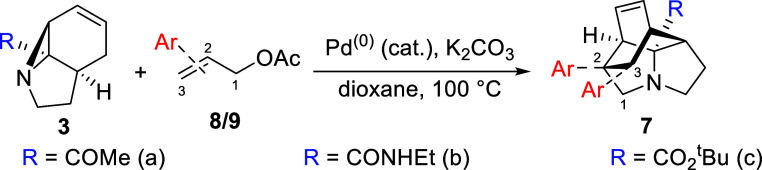

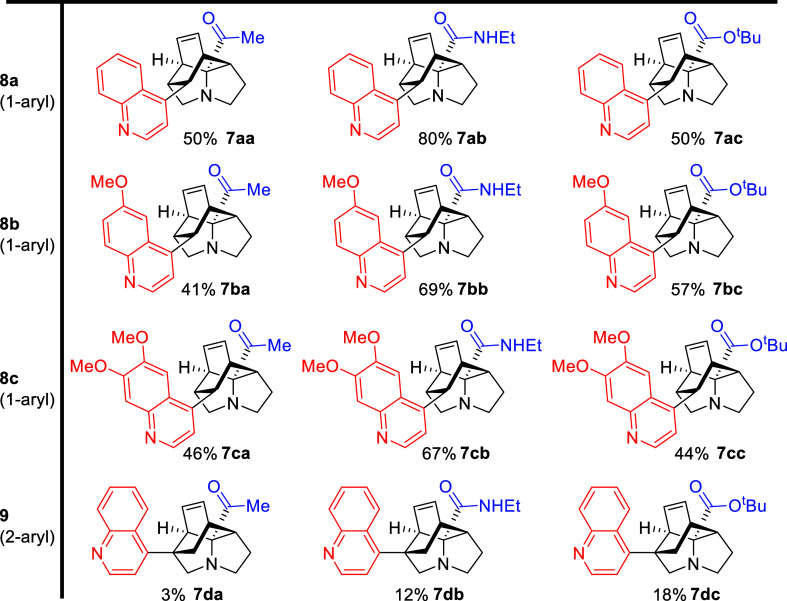
Isolated Yields from the Cascade Reactions of Aziridines **3** with Allylic Quinolines **8** and **9**

With this family of 12 compounds now available, their photophysical properties could be investigated. The focus was to explore TSCT between the nitrogen atom on the cage and the quinoline moiety,^5^ in particular relating this to *N*-aryl distance. Since the general effect of protonation on the two nitrogen atoms was expected to be similar to quinine, MeCN and 0.1 M H_2_SO_4_ were chosen as solvents to allow for the study of the unprotonated and doubly protonated forms of the compounds, respectively. Confirmation that double-protonation of these systems occurs in 0.1 M H_2_SO4 is shown through the NMR studies provided within the Supporting Information and is also consistent with the p*K*_a_ values of the conjugate acids calculated by density functional theory (DFT), which are also given in the Supporting Information (Table S4). This allows the SARs to be built around the changing parameters of the number of methoxy groups and the *N*-aryl distance. Use of an appropriate buffer can also give access to singly protonated systems, which we studied for **7aa** and **7da** (see Figure S1). However, as protonation is necessarily an equilibrium process, which in this case involves a relatively narrow range of p*K*_a_s, this gives a mixture of species that complicates the analysis of the resulting photophysics. This study therefore focuses on neutral and doubly protonated systems.

**Figure 1:**
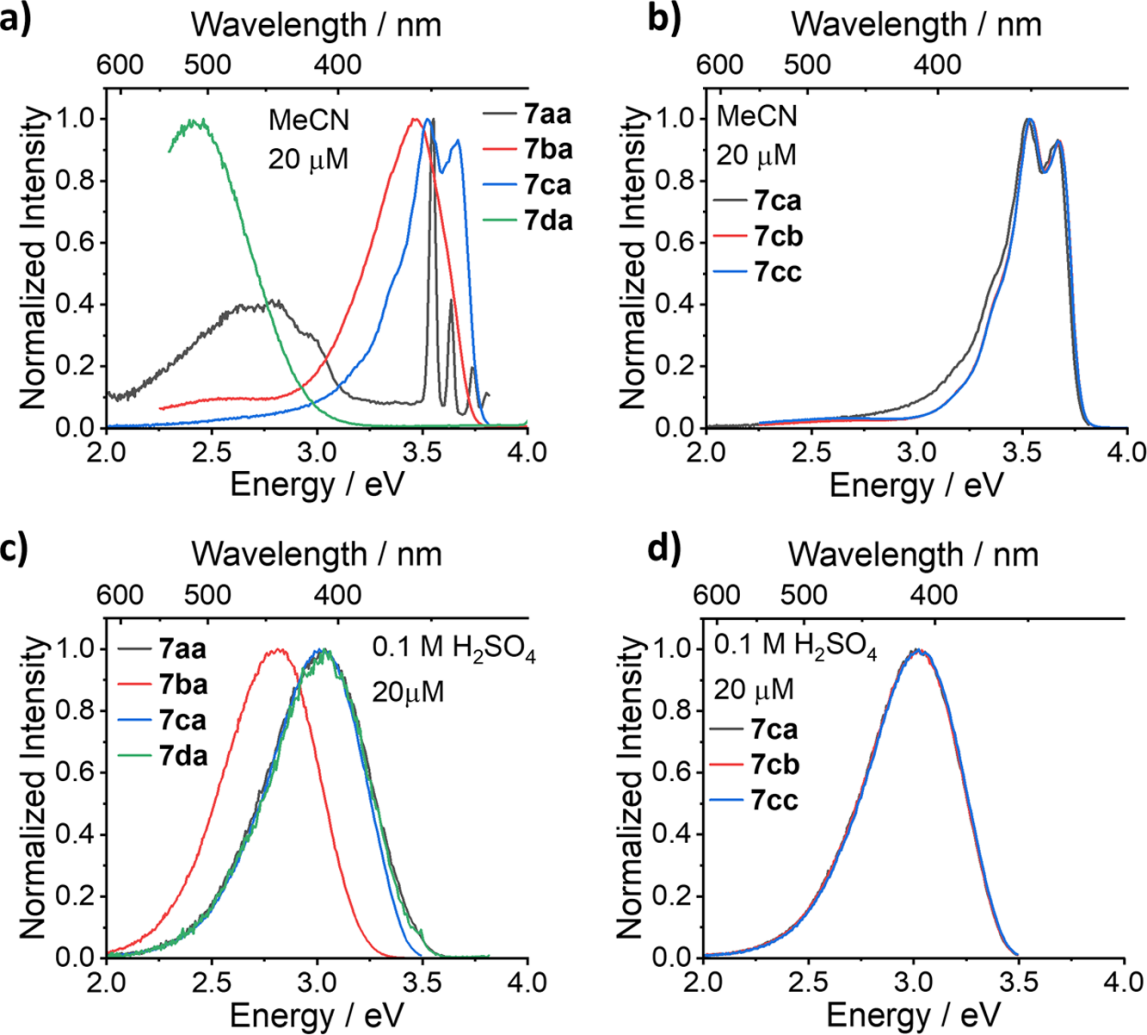
Photoluminescence spectra of **7aa**, **7ba**, **7ca**, and **7da** in (a) MeCN and (c) 0.1 M H_2_SO_4_ demonstrating the effect of the number of methoxy groups and tether position (**7da**) on the spectral profile and that changing the functionality α-to the cage nitrogen has no impact in either (b) MeCN or (d) 0.1 M H_2_SO_4_.

Figure 1 shows a comparison of the compounds across the rows and columns of Table 1 in both MeCN and 0.1 M H_2_SO_4_. Figure 1a, which is a comparison of compounds **7aa** to **7da** (i.e., going down the first column in MeCN), shows the impact of methoxylation and tether attachment location on the photophysics. Compounds **7ba** and **7ca** have clear locally excited (LE) emission peaks at around 3.5 eV, demonstrating the complete lack of TSCT in these systems. A comparison of the MeCN emission bands of **7ba** and **7ca** (and the rest of the **7bx** and **7cx** series) to the corresponding monosubstituted quinoline (**S2b**) and disubstituted quinoline (**S2c**) (Figure S2) shows matching vibronic character. This indicates that the emission in each case is coming solely from the aromatic quinoline moiety, thus further confirming the LE nature described above for these series.

For compound **7aa**, the intensity of the emission is much weaker with Raman scatter from the solvent visible at around 3.5 eV and the real emission onset being located at 3.2 eV. This change in emission is due to the emergence of TSCT in this system. However, **7da** has the most significantly different spectrum with strong TSCT emission that is red-shifted to the other compounds. This is consistent with the lone pair of nitrogen being closer in space to the quinoline unit in **7da** compared to that of the greater distance observed in **7aa**. Distances have been calculated for all compounds through DFT studies but crucially for the **7ax** and **7dx** series they are 4.6, 4.6, and 4.6 Å for **7aa**, **7ab**, and **7ac** and 3.7, 3.7, and 3.8 Å for **7da**, **7db**, and **7dc**, respectively (see Table S3 for additional details). The LE and CT nature of the different series that has been observed experimentally is also supported by DFT calculations, which demonstrate the trend of **7da** having the lowest energy CT state and with the CT states becoming higher in energy (i.e., more inaccessible) going from **7aa** to **7ba** to **7ca.** In contrast, the quinoline π–π* LE states are calculated to have energies in the order **7da** > **7aa** > **7ca** > **7ba** (Table S5 and Figure S17). Figure 1b shows a comparison across the second row of Table 1 in MeCN, clearly showing that varying the functionality α-to the cage nitrogen atom (i.e., ketone, amide, or ester) has very limited impact on the photophysical properties (**7ca**, **7cb**, and **7cc** all have the same spectral profile) but has an appreciable impact on the p*K*_a_ value of the tertiary amine, as indicated by DFT calculations (see Table S4). This therefore enables variation of the p*K*_a_ of the tertiary amine moiety while retaining essentially identical photophysical properties, suggesting that such compounds may have useful function as pH sensors.

Figure 1c,d shows the same comparisons but in 0.1 M H_2_SO_4_. Figure 1c shows that all compounds now have LE emission, with no CT emission visible. **7aa**, **7ca**, and **7da** have almost identical emission spectra with a peak at 3.0 eV. **7ba** has a slightly red-shifted emission peaking at 2.8 eV, and this is attributed to the methoxy group, which has an impact on the electronics in the protonated form. The emission profile of **7ba** is quite consistent with protonated quinine, which also has a single methoxy group.^5^ Again, comparing **7ba** and **7ca** to that of **S2b** and **S2c**, the emission spectra match in 0.1 M H_2_SO_4_. This shows that when the cage nitrogen atom lone pair is not involved in TSCT, the emission profile is dominated by the quinoline unit. Figure 1d shows that in 0.1 M H_2_SO_4_, the **7cx** series is again spectrally consistent, demonstrating that varying the functionality α-to the cage nitrogen atom has no impact on the photophysics. The trends described for the above series are consistent when comparing across all rows and down all columns in Table 1, and the complete photoluminescence and excitation spectra for all compounds, alongside the absorption spectra, can be found in the Supporting Information in Figures S3–S6.

Interestingly, the number of methoxy units on the quinoline unit affects the PLQY of the emission (Table 2), with the monomethoxy series (**7bx**) showing the highest PLQYs consistent with, and at times exceeding, the PLQY for quinine in 0.1 M H_2_SO_4_. However, having no methoxy units (**7ax** series) or having two methoxy units (**7cx** series) serves to reduce this quantum yield to either 6–8% or 25–30%, respectively. The position of attachment to the cage also does not seem to affect the quantum yield with the **7dx** series in 0.1 M H_2_SO_4_ being almost identical to the **7ax** series. The PLQY in MeCN is very low and similar for all compounds, with a slightly higher PLQY observed for the **7bx** and **7cx** series.

**Table 2:** PLQY Data for Tricyclic Quinine Analogues in 0.1 M H_2_SO_4_ and MeCN

	PLQY/%^a^			
compound	H_2_SO_4_	MeCN	PVA	PMMA
**7aa**	6	<1	<1	<1
**7ab**	8	<1	7	<1
**7ac**	7	2	<1	2
**7ba**	63	4	<1	1
**7bb**	67	5	6	<1
**7bc**	70	4	8	1
**7ca**	26	8	3	1
**7cb**	26	16	3	1
**7cc**	29	16	5	2
**7da**	5	<1	6	<1
**7db**	6	<1	6	<1
**7dc**	5	4	5	<1

a Values were obtained as the average of two repeats, apart from **7db**, which are single measurements.

**Figure 2:**
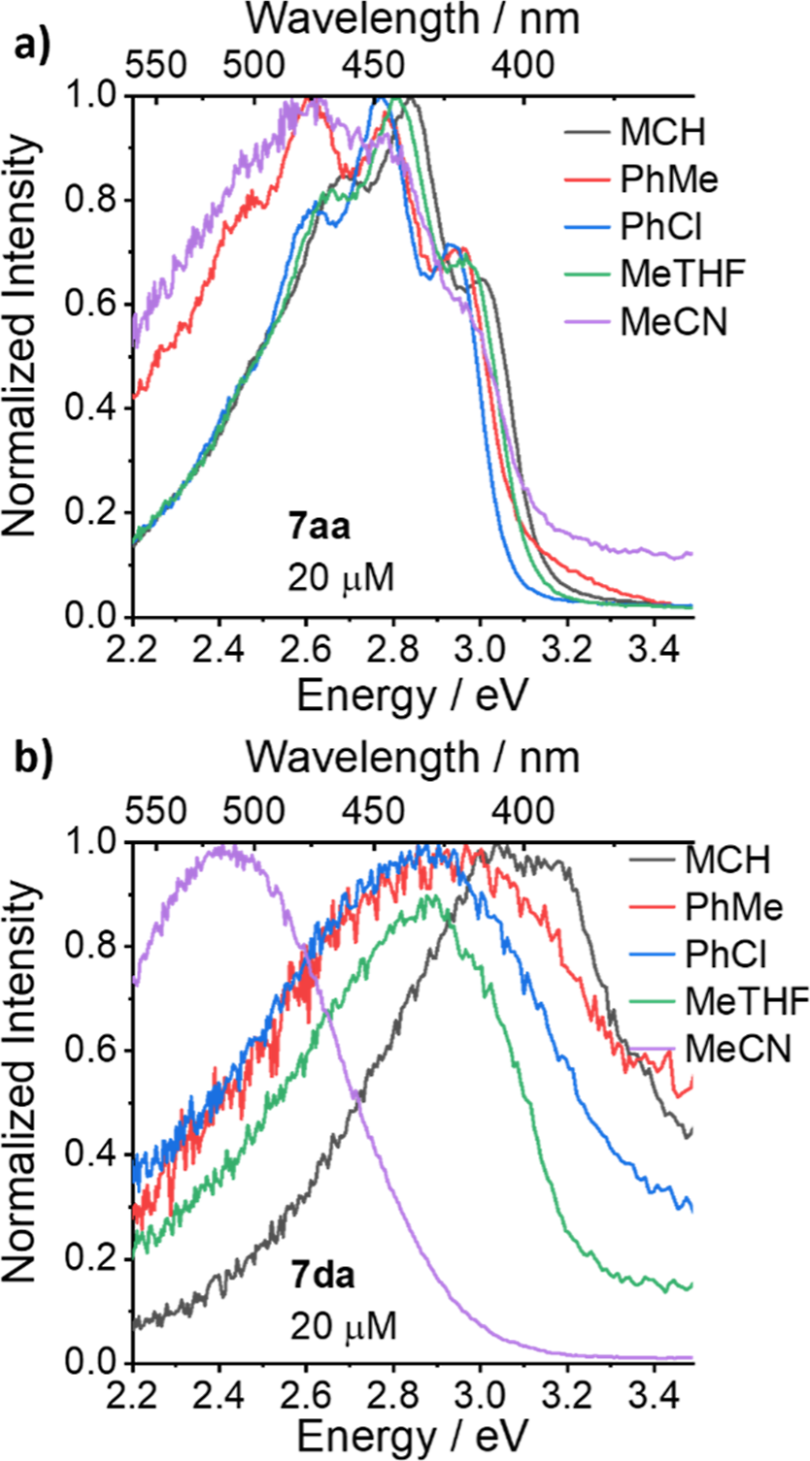
Steady-state photoluminescence spectra of (a) **7aa** and (b) **7da** in a series of solvents. MCH = methylcyclohexane; MeTHF = 2-methyltetrahydrofuran.

Given the observed TSCT character in the **7dx** series and the heavily quenched emission in the **7ax** series, a subsequent investigation exclusively into the position of the quinoline moiety in relation to the nitrogen atom within the cage was performed to explore the effect of the *N*-aryl distance on this TSCT state formation. **7aa** and **7da** were studied (Figure 2) in five different solvents with increasing polarity to observe potential solvatochromism effects. The absorption and excitation profiles in these different solvents can be found in Figures S7 and S8.

Figure 2a shows the emission of **7aa**, which has the quinoline at position 3 of the cage. We see little change in the emission peak on increasing the polarity of the solvents, and there is only a slight red shift in the peak on dissolving the compound in MeCN compared to less polar solvents, indicating weak TSCT character at best for **7aa**. **7aa** also shows a more vibronic character than the **7da** emission profile (Figure 2b), which has a broader emission spectrum characteristic of CT states and a strong bathochromic shift with increasing polarity. **7da** in methylcyclohexane has an emission peak of 3.1 eV, which undergoes a 0.7 eV shift to 2.4 eV when dissolved in MeCN compared with a shift of 0.4 eV between methylcyclohexane and MeCN for **7aa**. This shift in emission and increase in strength of TSCT can be rationalized through the position of the lone pair of the cage nitrogen being closer to the quinoline moiety and so giving clear indication of a TSCT state between the two components. This switching “on and off” of TSCT through a change in position by a single carbon bond shows the sensitivity of the formation of these states. Overall, this shows that the TSCT caused by the lone pair of the nitrogen atom in the cage unit can be effectively controlled via pH and protonation in solution, opening pathways to sensing.

**Figure 3:**
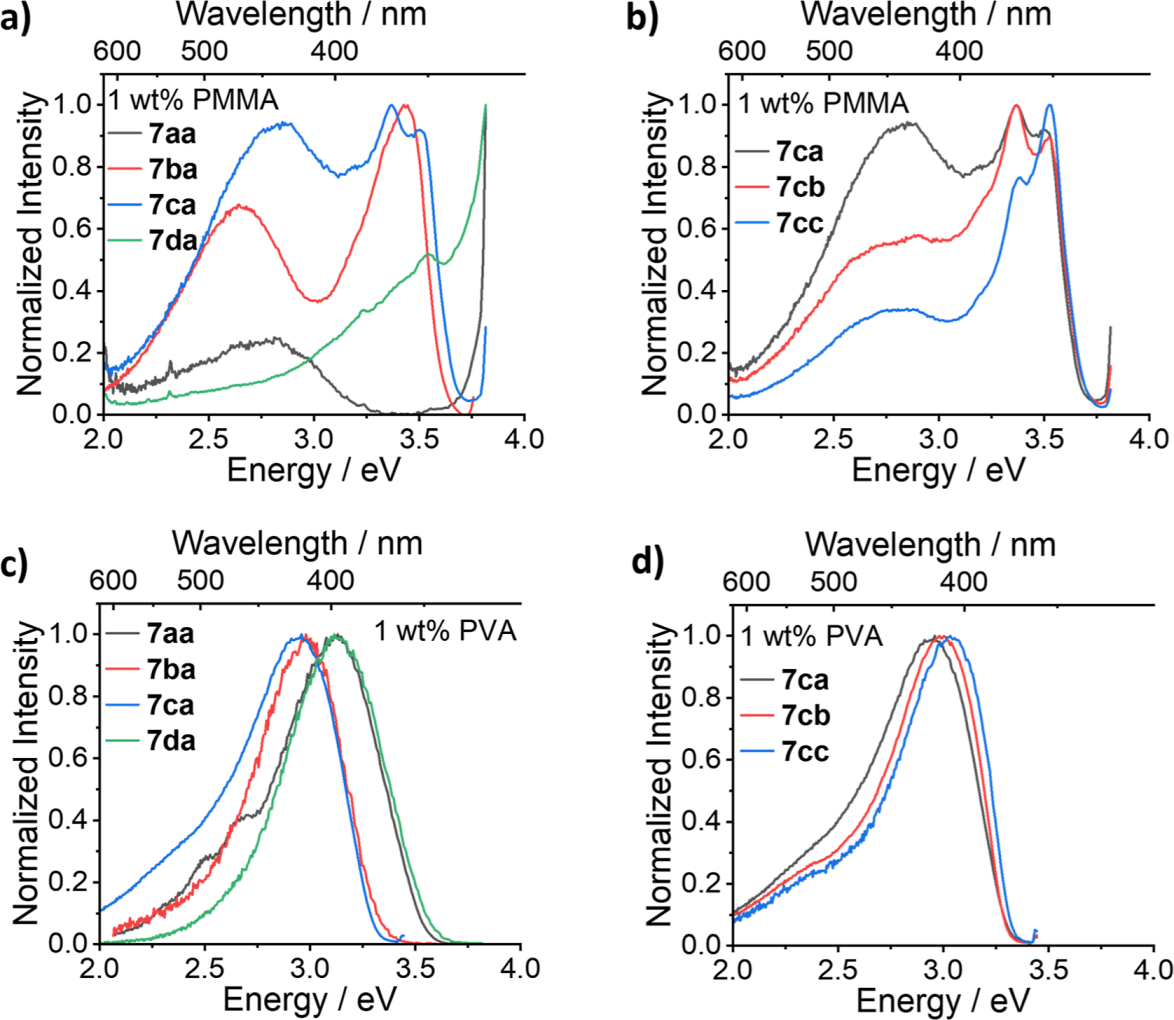
Photoluminescence spectra of **7aa**, **7ba**, **7ca**, and **7da** in (a) 1 wt % PMMA and (c) 1 wt % PVA demonstrating the effect of the number of methoxy groups and tether position (**7da**) on the spectral profile and that changing the functionality α-to the cage nitrogen has no impact in either (b) 1 wt % PMMA or (d) 1 wt % PVA.

A brief study was also performed on this set of compounds in the solid state in both poly(methyl methacrylate) (PMMA) and poly(vinylalcohol) (PVA) polymer hosts, which replicate the MeCN and 0.1 M H_2_SO_4_ environments, respectively, providing an indication of their behavior in solid-state applications. Generally, similar emission profiles were observed in films, with PMMA resembling MeCN and PVA resembling 0.1 M H_2_SO_4_ (see Figure 3), albeit with much reduced PLQY (see Table 2), consistent with aggregation-induced quenching as a result of the increased concentration (ca. 25 mM) within the films.^34,35^ High-concentration solutions (up to 1 mM) of compound **7ab** in MeCN and 0.1 M H_2_SO_4_ were measured (see Figures S9 and S10) to demonstrate the effect of this quenching. At high concentrations and longer excitation wavelengths, an aggregate species can be excited. This aggregate emits at an energy of 2.50 eV in both MeCN and 0.1 M H_2_SO_4._ The intensity of this aggregate emission grows with increasing concentration at the expense of the monomer emission in these solvents. The monomeric species is still present, giving emission from that species at the shorter wavelength excitations we use for the main study. However, the growing presence of the aggregate species, exemplified in the Supporting Information by exciting directly into the new absorption band, explains the severe drop in PLQY in the solid-state films.

Although this reduced PLQY limits their solid-state applications, further interesting differences were observed. Notably, new emission bands were observed for **7ba** and **7ca** in the PMMA films with peak emission energies at approximately 2.75 eV. These bands are in addition to the already existing emission bands at 3.5 eV, which we previously assigned as LE character based on the MeCN solution measurements. We attribute these new bands due to the formation of emissive aggregates in the films, which is a phenomenon consistent with previous studies of thin films.^36−38^ A similar new red band is observed for both **S2b** and **S2c** (Figure S2), which is consistent with aggregation of the aromatic unit in the films. For **7aa** and **7da** in PMMA, the spectral profiles are difficult to resolve because of the low PLQY.

Moving to the PVA films, which were drop-cast from 0.1 M H_2_SO_4_ to replicate the acid environment in solution, the emission is almost identical to that of the 0.1 M H_2_SO_4_ solutions, and the additional emission bands seen for **7bx** and **7cx** in the PMMA films are no longer present. This suggests that the formation of emissive aggregates is much more limited under these conditions, although not entirely absent, as will be discussed subsequently for the **7bx** series.

Comparing the effect of the functionality α-to the cage nitrogen, we generally observed very little change in emission within the **7ax**, **7dx**, and the **7cx** series as exemplified by the **7cx** series in Figure 3b,d for PMMA and PVA films, respectively. It is of note, however, that for the **7bx** series, the emission maxima exhibit greater variation in the PVA films compared to that in the 0.1 M H_2_SO_4_ solutions. Since this effect is only observed for the **7bx** series, we attribute these effects not to the change in α-substituent across the series but due to the influence of the monomethoxy quinolyl unit. If the α-substituent were the main cause of these shifts, it would be expected across the **7ax**, **7cx**, and **7dx** series, suggesting that it is the aromatic unit that drives the aggregation in the PVA films. The full photophysical characterization for both the PMMA and PVA can be found in Supporting Information Figures S11–S14.

## Conclusions

Control of CT character is an important parameter that can have an impact on a wide range of fields and applications, from CT in photosynthesis to the CT states that underpin phenomena such as TADF. In this work, we have shown that fine control of the TSCT character can be achieved through small alteration in the distance between a quinoline chromophore and an aliphatic amine moiety.^38^ Our results demonstrate that by simply changing the position of the quinoline unit with respect to the cage nitrogen atom, the CT character can either be enhanced or reduced. If the cage nitrogen is closer to the quinoline ring, a stronger TSCT species can be obtained. Importantly, for their application in sensors, the p*K*_a_ of the nitrogen atom on the cage can be modified and have minimal effect on the emission profile. This provides an additional parameter for variation, potentially making these compounds viable for use in biological sensing applications. Developing these SARs for the photoluminescence of CT and TSCT states demonstrates the need to have an in-depth understanding of the molecular level requirements for light-emitting applications. SARs for light-emitting materials are just as powerful as SARs for understanding bioactivity in the field of medicinal chemistry and will strengthen our ability to predict fluorophores of the future.

## Supporting Information Available

Synthetic chemistry discussion, experimental methods, synthesis of quinoline tethers, photochemical synthesis of tricyclic aziridine substrates, palladium-catalyzed cascade reactions, NMR spectra, photophysical data, theoretical calculations, PLQY data, and protonation studies on compound **7ab** (PDF)
