# Supplementary material for: A Modular Approach to Tuning Emissive N-Quinolyl Through-Space Charge Transfer States Using sp3-Scaffolds
Source: J Phys Chem B. 2024 Nov 4;128(45):11208–15. doi: 10.1021/acs.jpcb.4c05220 (PMC11571211; doi:10.1021/acs.jpcb.4c05220)

Supporting Information

A Modular Approach to Tuning Emissive *N*-Quinolyl Through-Space Charge Transfer States Using sp^3-Scaffolds.

Joseph Watson,[a] Ruth M. Pollard,[b] Mark T. Sims,[a] Marc K. Etherington*[b] and Jonathan P. Knowles*[a]

[a] Department of Applied Sciences, Northumbria University, Ellison Place, Newcastle upon Tyne, NE1 8ST, UK
E-mail: jonathan.p.knowles@northumbria.ac.uk

[b] Department of Mathematics, Physics and Electrical Engineering, Northumbria University, Ellison Place, Newcastle upon Tyne, NE1 8ST, UK
E-mail: marc.k.etherington@northumbria.ac.uk

Contents

1. **Discussion of Synthetic Chemistry** 3
2. **General Methods and Instrumentation** 5
3. **Synthesis of Quinoline Tethers** 5
4. **Photochemical Synthesis of Tricyclic Aziridine Substrates** 10
5. **Palladium-Catalyzed Cascade Reactions** 10
6. **Photophysical Studies** 16
7. **Theoretical Calculations** 28
8. **Protonation Studies on Compound 7ab** 32
9. **References** 34
10. **NMR Spectra** 37

1. Discussion of Synthetic Chemistry

Quinoline-containing tethers **8a-c** were prepared straightforwardly through standard heterocyclic synthesis as shown in Scheme S1. While compound **S3a** was commercially available, quinoline aldehydes **S3b** and **S3c** were prepared in moderate yield via Skraup synthesis followed by a benzylic oxidation with selenium dioxide.[1,2] All three aldehydes then underwent an efficient Grignard addition/acetylation sequence to yield the desired tethers **8**. These syntheses proved to be scalable, enabling gram quantities to be formed in some cases.

Routes to tether **9** were made somewhat more challenging by the need for the construction of a geminally disubstituted alkene. While various less modular multi-step approaches appeared possible, we observed that Kutsumura *et al*. recently reported[3] a one-pot bromination/cross-coupling strategy to enable the synthesis of 2-aryl substituted allylic alcohol derivatives. Such an approach would enable late-stage variation of the quinoline unit by introducing it in the penultimate cross-coupling step.

Scheme S1. Synthesis of tethers **8** and **9**.

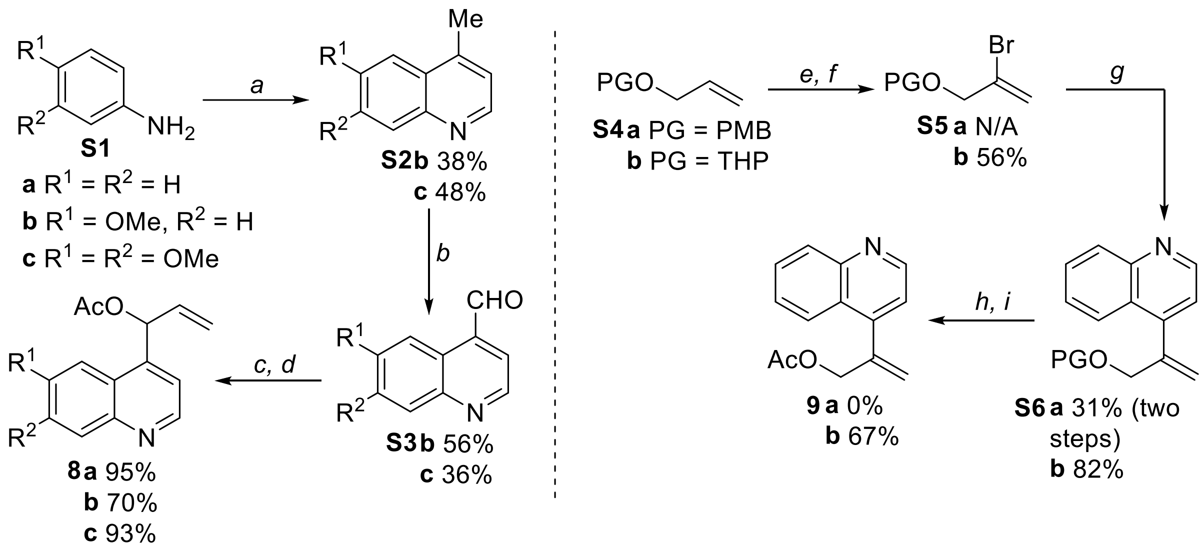

a) $FeCl_3$, methyl vinyl ketone, AcOH, Δ; b) SeO_2, H_2O, dioxane, 90 °C; c) $CH_2CHMgBr$, THF, 0 °C; d) Ac_2O, Et_3N, DMAP, CH_2Cl_2; e) $PyHBr_3$, K_2CO_3, MeCN; f) DBU, 60 °C; g) $ArB(OH)_2$, $Pd(PPh_3)_4$, Na_2CO_3, dioxane, Δ; h) aq. HCl, rt; i) Ac_2O, Et_3N, DMAP, CH_2Cl_2. PMB = 4-methyoxybenzyl. THP = 2-tetrahydropyranyl. DMAP = 4-dimethylaminopyridine.

We therefore prepared 4-methoxybenzyl (PMB)-protected allylic ether **S4a**. Bromination and cross-coupling proceeded as reported,[3] furnishing the PMB-protected quinoline **S6a** in reasonable yield (Scheme 2). However, PMB deprotection proved troublesome, leading to either full recovery of the starting material or degradation. We therefore considered different protecting groups and after some experimentation found that 2-tetrahydropyranyl (THP) worked efficiently. While a one-pot bromination/cross-coupling approach also proved possible in this case, isolation of bromide **S5b** gave improved results in the subsequent cross-coupling, with quinoline **S6b** being isolated in good yield with a reaction that could be scaled to 1 g. Deprotection and acetylation to form **9b** then proved straightforward under standard conditions.

With the required quinoline tethers in hand, three aziridines **3a-c** were prepared by previously reported methods[4] and the compounds were reacted under Pd catalysis to form a small compound library. This proved successful, with 1-substituted tethers **8a-c** reacting as expected to form tricycles **7aa-cc** in good to moderate yield (Table 1). Yields are unoptimized except for that for the reaction forming **7ab**, which was shown to be scalable to 1 mmol without reduction in yield. While reactions of *tert*-butyl substituted aziridine **3c** proceeded somewhat more slowly, as we have previously

reported,[5] all reactions were found to be complete within 15 h at 100 °C. Increasing substitution of the quinoline necessarily leads to an increase in electron density (i.e. moving from **8a** to **8c**) and might thus be expected to impact on the rate limiting Diels-Alder cycloaddition within the cascade process; however, ultimately little difference in conversion vs time profile was observed, suggesting all quinoline moieties are effective in activating this step. Reactions involving 2-substituted tether **9** proved somewhat more challenging, with reactions proceeding significantly more slowly and redox-based side reactions competing at higher temperature. However, increased reaction times led to isolable levels of product formation in all cases. As expected, the reaction to form *tert*-butyl ester **7dc** proved to be the most challenging; but this was improved by pre-mixing tricyclic aziridine **3c** with the Pd-catalyst, with the addition of allylic acetate **9** following this initial allylation step. This was then followed by a separate cycloaddition step at higher temperature. These conditions enabled isolation of the desired product in 18% yield, with this improved yield from pre-mixing (c.f. 1% without) suggesting that a mismatch in rates of oxidative addition may be present in the case of allylic acetate **9**. Further, the reduced rate within the Diels-Alder reactions to form series **7dx** suggests that the 2-substituted allyl component is appreciably less activated, potentially due to reduced conjugation of the alkene with the quinoline moiety or increased steric demand. However, despite some reduction in yield for these latter reactions, the process gave broad access to the compounds shown in Table 1, tolerating significant changes in electron density of the activating quinoline moiety and providing a diverse range of compounds from seven easily prepared building blocks in a single step.

2. General Methods and Instrumentation

All palladium-catalysed processes were carried out using Schlenk technique under argon using commercially available anhydrous dioxane unless stated otherwise. Chemicals were obtained from commercial sources unless otherwise stated. THF and toluene were dried over 3 Å activated molecular sieves for 3 days prior to use. Column chromatography was performed using 40-60 mesh silica powder. $EtOH/NH_3$ refers to an 8:1 v/v mixture of EtOH and saturated aqueous ammonia solution respectively. NMR spectroscopic analysis was performed using Jeol ECS 400 MHz instrument. Chemical shifts are reported in δ ppm. ^{13}C NMR are referenced to solvent as internal standard ($CDCl_3$ or DMSO). Data are reported as follows: chemical shift, integration, multiplicity (s = singlet, d = doublet, t = triplet, q = quartet, p = pentet, hept = heptet, dd = doublet of doublets, dt = doublet of triplets, td = triplet of doublets, tt = triplet of triplets, ddt = doublet of doublet of triplets, ddd = doublet of doublet of doublets, m = multiplet), coupling constants (Hz), and integration. Mass spectrometry analysis was performed using electrospray ionisation.

3. Synthesis of Quinoline Tethers

1-(quinolin-4-yl)-1-acetoxy-prop-2-ene **8a**

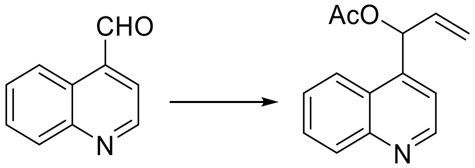

To a stirred solution of quinoline-4-carboxaldehyde (500 mg, 3.18 mmol) in dry THF (10 mL) at -10 °C under nitrogen was added vinylmagnesium bromide (3.8 mL of a 1.0 M solution in THF, 3.8 mmol) dropwise. The reaction was stirred for 10 min, allowed to warm to rt over 30 min and quenched by the addition of sat. aq. NH_4Cl (12 mL). The mixture was extracted with EtOAc (2 × 20 mL) and the combined organic phase washed with brine (20 mL), dried ($MgSO_4$) and evaporated to afford the crude alcohol (583 mg) as an orange oil which was used directly. To a stirred solution of the crude allylic alcohol (583 mg, 3.15 mmol) in DCM (10 mL) at 0 °C under nitrogen was added triethylamine (0.90 mL, 6.5 mmol) and DMAP (10 mg, 0.08 mmol), followed by acetic anhydride (0.60 mL, 5.4 mmol) dropwise. The reaction was allowed to warm to rt, stirred for 18 h and partitioned between DCM (15 mL) and sat. aq. $NaHCO_3$ (25 mL). The phases were separated, the aqueous phase extracted with DCM (10 mL) and the combined organic phase dried ($MgSO_4$) and evaporated to give an orange oil. Purification by silica gel chromatography (EtOAc/petrol, 1:4 to 3:7 as eluent) afforded the title compound (594 mg, 82% over two steps) as a clear oil. Data was in accord with that reported.[6]

4-methyl-6-methoxyquinoline **S2b**

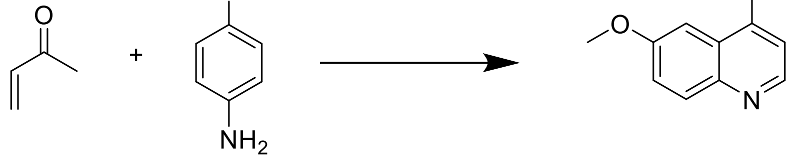

A solution of $FeCl_3.6H_2O$ (5.40 g, 20 mmol) and *p*-anisidine **S1b** (1.26 g, 10 mmol) in acetic acid (32 mL) was heated to 60°C before the dropwise addition of methyl vinyl ketone (0.880 mL, 10.5 mmol). The reaction was then heated to reflux and monitored by TLC (20/80 Et_2O/DCM) until completion. After 4 h the reaction was cooled to RT and solvent evaporated. Water (6 mL) was added and the

mixture basified to pH 10-11 using 50% w/w aq. NaOH. The mixture was concentrated and the residue extracted using DCM (6 x 50 mL), filtering through Celite. The filtrate was washed with 10% w/w aq. K_2CO_3 (150 mL) and the organic phase dried over ($MgSO_4$) and evaporated. Purification by silica gel chromatography (Et_2O/DCM 1:4 as eluent) afforded the title compound (661 mg, 38%) as an orange oil. Spectral data was in accord with that previously reported.[1]

6-methoxyquinoline-4-carbaldehyde **S3b**

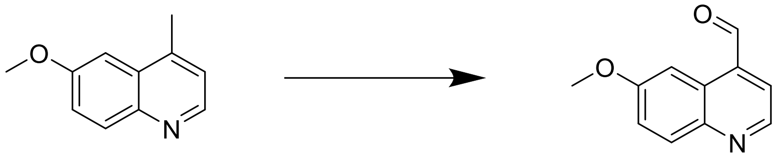

Substrate **S2b** (796 mg, 4.60 mmol) was dissolved in dioxane (2.7 mL) and heated to 80 °C before the dropwise addition of a solution of SeO_2 (632 mg, 5.69 mmol) in a mixture of dioxane/H_2O (5.33 mL/1.33 mL) over 10 min. The reaction was heated to 90°C and stirred overnight. After 16 h the rection was then cooled to rt, diluted with sat. aq. $NaHCO_3$ (12 mL) and extracted with DCM (4 x 25 mL). The organic phase was dried ($MgSO_4$) and evaporated. Purification by silica gel chromatography (Et_2O/DCM 1:3 as eluent) afforded the title compound as a dark yellow solid (475 mg, 56%). Spectral data was in accord with that previously reported.[2]

1-(6-methoxyquinolin-4-yl)-1-acetoxy-prop-2-ene **8b**

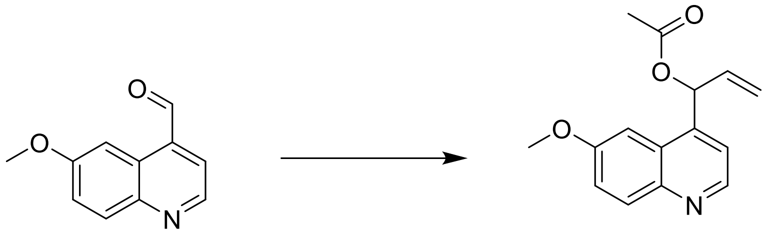

Substrate **S3b** was dissolved in dry THF (9.5 mL) and cooled to 0 °C under argon before the dropwise addition of vinylmagnesium bromide (1.0 M in THF, 3.45 mL, 3.5 mmol). The reaction was stirred for 10 min at 0 °C before warming to rt. After 30 min the reaction was quenched with saturated aq. NH_4Cl (12 mL) and the mixture extracted with EtOAc (2 x 20mL). The combined organic phase was washed with brine (20 mL) dried ($MgSO_4$) and evaporated to afford the crude product as an orange oil (0.533g) which was used directly. To a stirred solution of the crude allylic alcohol (533 mg, 2.48 mmol) in DCM (9 mL) at 0 °C under nitrogen was added triethylamine (0.72 mL, 5.2 mmol) and DMAP (9.0 mg, 0.074 mmol), followed by acetic anhydride (0.34 mL, 4.3 mmol) dropwise. The reaction was allowed to warm to rt, stirred for 18 h and partitioned between DCM (15 mL) and sat. aq. $NaHCO_3$ (25 mL). The phases were separated, the aqueous phase extracted with DCM (10 mL) and the combined organic phase dried ($MgSO_4$) and evaporated to give an orange oil. Purification by silica gel chromatography (EtOAc/petrol, 1:4 to 3:7 as eluent) afforded the title compound (466 mg, 71% over two steps) as a yellow oil. ν_{max} /cm^{-1} (film) 2938, 1742, 1621, 1593, 1508, 1474, 1371 and 1229; ^{1}H NMR ($CDCl_3$, 400 MHz): δ 2.18 (3H, s), 3.92 (3H, s), 5.32 – 5.37 (2H, m), 6.10 (1H, ddd, *J* 17.5, 10.1, 5.7), 6.88 (1H, dd, *J* 5.6, 1.7), 7.27 (1H, d, *J* 2.7), 7.39 (1H, dd, *J* 9.3, 2.8), 7.46 (1H, d, *J* 4.6), 8.08 (1H, d, *J* 9.2) and 8.76 (1H, d, *J* 4.6); ^{13}C{^{1}H} NMR ($CDCl_3$, 101 MHz): δ 21.2, 55.7, 72.3, 101.7, 119.0, 119.3, 122.5, 126.7, 131.3, 134.4, 143.2, 144.0, 147.1, 158.2 and 169.9; HRMS (ESI$^+$-Orbitrap) m/z: $[M+H]^+$ calcd for $C_{15}H_{16}NO_3$ 258.1130; found 258.1123.

4-methyl-6,7-dimethoxyquinoline **S2c**

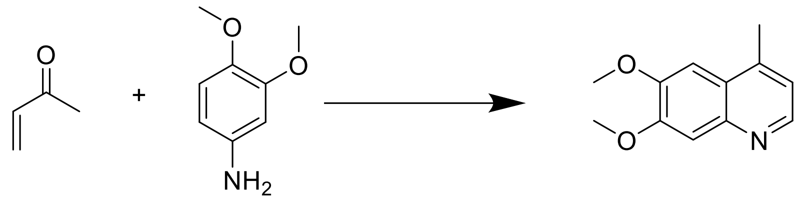

A solution of $FeCl_3.6H_2O$ (5.40 g, 20 mmol) and aniline **S1c** (1.53 g, 10 mmol) in acetic acid (32mL) was heated to 60 °C before the dropwise addition of methyl vinyl ketone (0.880 mL, 10.5 mmol). The reaction was then heated to reflux and monitored by TLC (4/94 $Et_2O.NH_3$/DCM) until completion. After 4 h the reaction was then cooled, evaporated and redissolved in water (8 mL). The mixture was basified to pH 10-11 using 50% w/w aq. NaOH. The mixture was concentrated and the residue extracted using DCM (6 x 50 mL), filtering through Celite. The filtrate was washed with 10% w/w aq. K_2CO_3 (150 mL) and the organic phase dried over ($MgSO_4$) and evaporated. The compound was purified by silica gel chromatography (4/94 EtOH:NH_3/DCM as eluent) to afford the title compound (967 mg, 48%) as a yellow oil. Spectral data was in accord with that previously reported.[1]

6,7-dimethoxyquinoline-4-carbaldehyde **S3c**

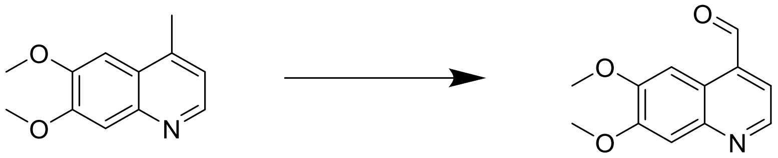

Substrate **S2c** (967 mg, 4.76 mmol) was dissolved in dioxane (3.2 mL) and heated to 80 °C before the dropwise addition of a solution of SeO_2 (792 mg, 7.14 mmol) in a mixture of dioxane/H_2O (6.67mL/1.67mL) over 10 min. The reaction was then heated to 90 °C. After 16 h the rection was cooled to rt, diluted with sat. aq. $NaHCO_3$ (15 mL) and extracted with DCM (4 x 30 mL). The organic phase was dried ($MgSO_4$) and evaporated. Purification by silica gel chromatography (Et_2O/DCM 1:3 as eluent) afforded the title compound (369 mg, 36%) as a dark yellow solid.[7]

1-(6-methoxyquinolin-4-yl)-1-acetoxy-prop-2-ene **8c**

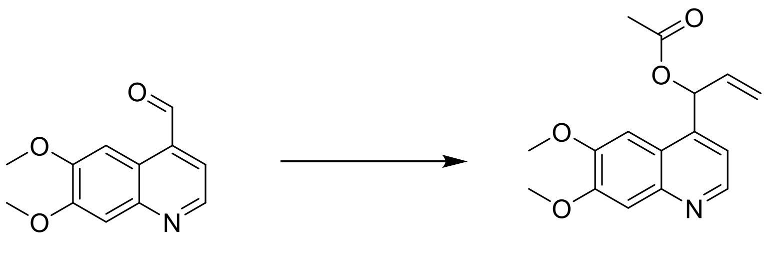

Substrate **3c** (369 mg, 1.7 mmol) was dissolved in dry THF (7.4 mL) and cooled to 0°C under argon before the dropwise addition of vinylmagnesium bromide (2.04 mL of a 1.0 M solution in THF, 2.0 mmol). The reaction was stirred for 10 min at 0 °C and warmed to rt. After a further 30 min the reaction was quenched with saturated aq. NH_4Cl (12 mL) and the mixture extracted with EtOAc (2 x 20 mL). The combined organic phase was washed with brine (20mL) dried ($MgSO_4$) and evaporated to afford the crude product as an orange oil (409 mg) which was used directly. To a stirred solution of the crude allylic alcohol (409 mg, 2.48 mmol) in DCM (7 mL) at 0 °C under nitrogen was added triethylamine (0.50 mL, 3.37 mmol) and DMAP (6 mg, 0.051 mmol), followed by acetic anhydride (0.27mL, 2.9 mmol) dropwise. The reaction was allowed to warm to rt, stirred for 18 h and partitioned between DCM (15 mL) and sat. aq. $NaHCO_3$ (25 mL). The phases were separated, the aqueous phase extracted with

DCM (10 mL) and the combined organic phase dried ($MgSO_4$) and evaporated to give an orange oil. Purification by silica gel chromatography ($EtOH.NH_3$/DCM, 4% as eluent) afforded the title compound (465 mg, 91% over two steps) as a yellow oil. ^1H NMR ($CDCl_3$, 400 MHz): δ 2.16 (3H, s), 3.98 (3H, s), 4.01 (3H, s), 5.26 – 5.36 (2H, m), 6.09 (1H, ddd, *J* 17.0, 10.5, 5.7), 6.84 (1H, d, *J* 5.6), 7.24 (1H, s), 7.32 (1H, d, *J* 4.5), 7.43 (1H, s) and 8.70 (1H, d, *J* 4.9); $^{13}C\{^1H\}$ NMR ($CDCl_3$, 101 MHz): δ 21.2, 56.1, 56.2, 72.5, 101.5, 108.8, 117.5, 118.6, 121.1, 134.6, 141.9, 145.9, 148.1, 148.1, 149.9, 152.2 and 170.0.

Pyridinium tribromide **S7**

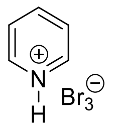

Prepared via a modified procedure.[8] Stirred aqueous HBr (48% in H_2O, 15mL, 130 mmol) was cooled to 0°C before the dropwise addition of pyridine (2.37 mL, 29.3 mmol). After 5 min Br_2 (1.68 mL) was added dropwise, followed by water (20mL) to aid stirring. The red precipitate formed was collected by filtration and washed with cold water (5 mL). The product was purified by recrystallization from acetic acid to afford the title compound (4.13 g, 44%) as a red solid.[8]

Allylic ether **S4b**

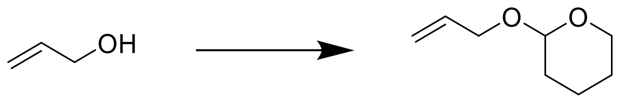

Allyl alcohol (1.36 mL, 20 mmol) and p-toluenesulfonic acid (344 mg, 2.0 mmol) were dissolved in dry THF (10 mL) and cooled to 0°C before the addition of 3,4-dihydro-2H-pyran (2.74 mL, 30 mmol). The reaction was stirred overnight, diluted with EtOAc (20mL) and washed with sat. aq. $NaHCO_3$ (20 mL) and brine (20 mL). The organic phase was dried ($MgSO_4$) and evaporated. Purification by silica gel chromatography (10/90 EtOAc/petrol as eluent) afforded the title compound (2.30 g, 81 %) as a clear oil. Spectral data was in accord with that previously reported.[9]

Brominated allylic ether **S5b**

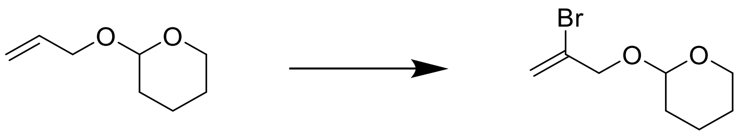

Using an adapted procedure,[3] substrate **S4b** (4.35 g, 30.6 mmol) was dissolved in MeCN (130 mL) and K_2CO_3 (4.65 g, 33.7 mmol) added. $PyHBr_3$ **S7** (10.76 g, 33.7 mmol) was added to the stirred suspension and the reaction stirred overnight at rt. After 16 h the reaction was cooled to 0°C and DBU (5.03 mL, 33.7 mmol) was added dropwise and the mixture warmed to 60°C. After 2 h the reaction was filtered through Celite, eluting with EtOAc. Evaporation gave the crude product as a red oil which was purified by silica gel chromatography (EtOAc/petrol, 8/92 as eluent) to afford the title compound (3.75 g, 56%) as a pale yellow oil. ν_{max} /cm^{-1} (film) 2942, 1638, 1454, 1441 and 1119; ^1H NMR ($CDCl_3$, 400 MHz): δ 1.46 – 1.89 (6H, m), 3.51 (1H, dtd, *J* 11.1, 4.3, 1.6), 3.85 (1H, ddd, *J* 11.4, 9.0, 3.2), 4.14 (1H, dt, *J* 14.2, 1.2), 4.27 (1H, dt, *J* 14.3, 1.4), 4.68 (1H, t, *J* 3.5), 5.58 (1H, s) and 5.91 (1H, q, *J* 1.6); $^{13}C\{^1H\}$ NMR ($CDCl_3$, 101 MHz): δ 19.1, 25.4, 30.4, 62.2, 70.7, 97.5, 117.6 and 129.5; HRMS: molecular ion not observed.

Protected quinoline tether **S6b**

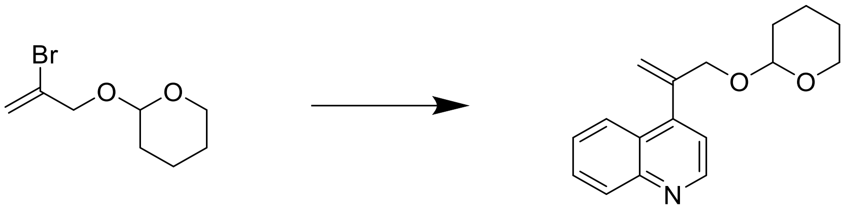

To a solution of bromide **S5b** (960 mg, 4.34 mmol) in dioxane (26 mL) was added 2 M aq. Na_2CO_3 (4.2 mL, 8.6 mmol) and the stirred mixture degassed by sparging with argon. After 5 min quinoline-4-boronic acid (720 mg, 4.16 mmol) and $Pd(PPh_3)_4$ (400 mg, 0.346 mmol) were added, the mixture sparged for a further 1.5 min and the mixture heated to reflux under argon with vigorous stirring. After 16 h the reaction was cooled to rt, diluted with water (100 mL) and extracted with EtOAc (3 × 50 mL). The combined organic phase was dried ($MgSO_4$) and evaporated to give an orange oil. Purification by silica gel chromatography (EtOH/NH_3 in DCM, 1% to 2% as eluent) afforded the title compound (0.92 g, 82%) as a yellow oil. ν_{max} /cm^{-1} (film) 2942, 1566, 1506, 1438 and 1184; ^{1}H NMR ($CDCl_3$, 400 MHz): δ 1.43 – 1.83 (6H, m), 3.46 (1H, dt, *J* 10.1, 4.5), 3.74 (1H, td, *J* 10.2, 8.6, 3.1), 4.29 (1H, d, J 13.7), 4.56 (1H, d, *J* 13.5), 4.71 (1H, t, *J* 3.4), 5.32 (1H, s), 5.79 (1H, s), 7.24 (1H, s), 7.53 (1H, t, *J* 7.7), 8.06 (1H, d, J 8.5), 8.11 (1H, d, *J* 8.5) and 8.86 (1H, d, *J* 4.3); ^{13}C{^{1}H} NMR ($CDCl_3$, 101 MHz): δ 19.3, 25.5, 30.5, 62.1, 69.7, 98.0, 118.0, 120.3, 125.6, 126.6, 126.8, 128.5, 128.7, 129.4, 129.9, 132.0, 132.1, 132.2, 142.7, 147.3, 148.6 and 149.9; HRMS (ESI^{+}-Orbitrap) m/z: [M+H]$^{+}$ calcd for $C_{17}H_{20}NO_2$ 270.1494; found 270.1486.

2-quinoline allylic alcohol **S8**

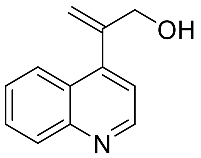

To a solution of substrate **S6b** (1.13 g, 4.21 mmol) in THF (22 mL) at rt was added a 2 M aq. HCl (22 mL, 44 mmol) and the reaction stirred at rt. After 19 h the reaction was concentrated in vacuo, neutralised with aq. Na_2CO_3 (7%, 150 mL) and the mixture extracted with DCM (3 × 60 mL). The combined organic phase was dried ($MgSO_4$) and evaporated to give a yellow oil. Purification by silica gel chromatography (EtOAc/petrol, 3:7 to 9:1 as eluent) afforded the title compound (0.70 g, 90%) as a light yellow oil. ν_{max} /cm^{-1} (film) 3232, 2853, 1581, 1507 and 1056; ^{1}H NMR ($CDCl_3$, 400 MHz): δ 2.09 (1H, brs), 4.49 (2H, s), 5.32 (1H, s), 5.78 (1H, s), 7.23 (1H, d, *J* 4.4), 7.53 (1H, t, *J* 7.6), 7.71 (1H, t, *J* 7.7), 8.02 (1H, d, *J* 8.5), 8.10 (1H, d, *J* 8.4) and 8.82 (1H, d, *J* 4.4); ^{13}C{^{1}H} NMR ($CDCl_3$, 101 MHz): δ 66.3, 116.6, 120.3, 125.4, 126.8, 129.6, 129.9, 145.4, 146.9, 148.5 and 149.9; HRMS (ESI^{+}-Orbitrap) m/z: [M+H]$^{+}$ calcd for $C_{12}H_{12}NO$ 186.0919; found 186.0913.

2-quinoline allylic acetate **9b**

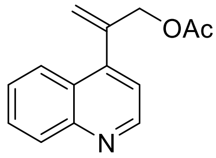

To a stirred solution of substrate **S8** (210 mg, 1.14 mmol) in DCM (8 mL) was added triethylamine (0.27 mL, 1.9 mmol), DMAP (3 mg, 0.025 mmol) and acetic anhydride (0.16 mL, 1.7 mmol). The mixture was stirred at rt for 2.5 h, quenched with sat. aq. $NaHCO_3$ (25 mL) and extracted with DCM (2 × 20 mL). The combined organic phase was dried ($MgSO_4$) and evaporated to give the crude product as a yellow oil. Purification by silica gel chromatography (EtOAc/petrol, 1:4 to 2:3 as eluent) afforded

the title compound (192 mg, 74%) as a clear oil. ν_{max} /cm^{-1} (film) 3061, 1738, 1566, 1506, 1375 and 1221; ^{1}H NMR ($CDCl_3$, 400 MHz): δ 2.04 (3H, s), 4.91 (1H, s), 5.36 (1H, s), 5.73 (1H, s), 7.25 (1H, s), 7.55 (1H, t, J 7.6), 7.72 (1H, t, J 7.7), 8.03 (1H, d, J 8.3), 8.12 (1H, d, J 8.6) and 8.88 (1H, d, J 4.3); ^{13}C{^{1}H} NMR ($CDCl_3$, 101 MHz): δ 20.9, 66.7, 119.1, 120.3, 125.3, 126.7, 126.9, 129.6, 130.0, 140.7, 146.2, 148.6, 149.9 and 170.6; HRMS (ESI$^+$-Orbitrap) m/z: $[M+H]^+$ calcd for $C_{14}H_{14}NO_2$ 227.0946; found 227.0938.

4. Photochemical Synthesis of Tricyclic Aziridine Substrates

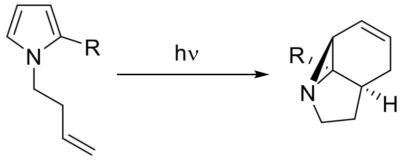

The synthesis of tricyclic aziridines **3a**, **3b**, and **3c** was performed from the corresponding *N*-butenyl pyrroles as previously described.[4,10] In all cases, a single 36 W UVC lamp enclosed in an FEP flow reactor was employed.

5. Palladium-Catalyzed Cascade Reactions

General procedure 1[5]

To a dried Schlenk tube under nitrogen was added palladium(II) acetate (0.10 equiv.), triphenylphosphine (0.40 equiv.), potassium carbonate (1.3 equiv.) and dry dioxane (8 mL/mmol). The mixture was sparged with argon, stirred for 5 min and a solution of aziridine substrate (1.0 equiv.) and allylic acetate (1.1 equiv.) in dry dioxane (8 mL/mmol) was added. The mixture was sparged with argon and heated to reflux. After 15 h the reaction was cooled and passed through a silica plug, eluting with EtOH/NH_3 in DCM (15%, 50 mL). The filtrate was concentrated and the residue purified by silica gel chromatography.

Quinoline **7aa**

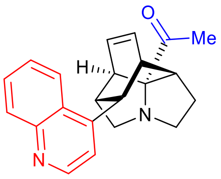

Following General Procedure 1 with aziridine **3a** (79 mg, 0.485 mmol) and allylic acetate **8a** (123 mg, 0.541 mmol). Purified by silica gel chromatography (EtOH.NH_3/DCM as eluent) to afford the title compound (81 mg, 50%) as a yellow oil. ν_{max} /cm^{-1} (film) 2943, 2869, 1703, 1587, 1511 and 1353; ^{1}H NMR ($CDCl_3$, 400 MHz): δ 1.88 – 2.00 (1H, m), 2.08 (1H, qd, *J* 10.2, 9.1, 5.0), 2.28 (3H, s), 2.45 – 2.46 (2H, m), 2.69 – 2.79 (2H, m), 3.07 – 3.25 (2H, m), 3.31 (1H, t, *J* 5.5), 3.62 (1H, s), 3.72 (1H, dd, *J* 11.6, 4.3), 6.00 (1 H, t, *J* 7.3), 6.19 (1 H, t, *J* 7.3), 6.86 (1 H, d, *J* 4.5), 7.56 (1 H, t, *J* 7.7), 7.67 (1 H, t, *J* 7.7), 7.99 (1 H, d, *J* 8.7), 8.08 (1 H, d, *J* 8.3), 8.72 (1 H, d, *J* 4.6); ^{13}C{^{1}H} NMR ($CDCl_3$, 101 MHz): δ 25.2, 28.0, 41.1, 41.9, 44.1, 45.7, 46.8, 57.7, 64.5, 85.5, 121.2, 122.7, 126.7, 127.1, 128.4, 128.9, 130.7, 136.1, 148.2, 149.7, 150.0 and 211.7; HRMS (ESI$^+$-Orbitrap) m/z: $[M+H]^+$ calcd for $C_{22}H_{22}N_2O$ 331.1811; found 331.1804.

Quinoline **7ba**

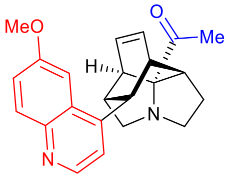

Following General Procedure 1 with aziridine **3a** (79 mg, 0.485 mmol) and allylic acetate **8b** (139 mg, 0.541 mmol). Purified by silica gel chromatography (EtOH.NH_3/DCM as eluent) to afford the title compound (73 mg, 41%) as a yellow oil. ν_{max} /cm^{-1} (film) 2932, 2861, 1700, 1621, 1587, 1508 and 1429; ^{1}H NMR ($CDCl_3$, 400 MHz): δ 1.88 – 2.01 (1H, m), 2.13 (1H, dq, *J* 16.3, 6.5 Hz, 1H), 2.32 (3H, s), 2.50 – 2.56 (1H, m), 2.60 (1H, d, *J* 11.6), 2.78 (1H, d, *J* 9.1), 2.82 – 2.87 (1H, m), 3.13 – 3.31 (2H, m), 3.36 (1H, t, *J* 5.5), 3.55 (1H, s), 3.79 (1H, dd, *J* 11.7, 4.4), 3.98 (3H, s), 6.08 (1H, t, *J* 7.3), 6.23 (1H, t, *J* 7.4), 6.88 (1H, d, *J* 4.7), 7.39 (1H, d, *J* 9.0), 8.03 (1H, d, *J* 9.2) and 8.63 (1H, d, *J* 4.5); ^{13}C{^{1}H} NMR ($CDCl_3$, 101 MHz): δ 28.4, 31.2, 43.8, 45.4, 47.2, 48.9, 50.1, 58.7, 61.0, 67.8, 88.7, 104.7, 123.9, 124.5, 131.2, 131.5, 135.4, 139.4, 147.5, 150.5, 151.6, 161.0 and 214.9; HRMS (ESI$^+$-Orbitrap) m/z: $[M+H]^+$ calcd for $C_{23}H_{24}N_2O_2$ 361.1917; found 361.1910.

Quinoline **7ca**

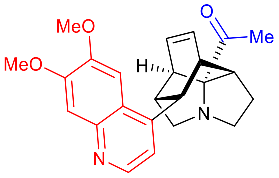

Following General Procedure 1 with aziridine **3a** (79 mg, 0.485 mmol) and allylic acetate **8c** (155 mg, 0.541 mmol). Purified by silica gel chromatography (EtOH/NH_3 in Et_2O, 2% to 25% as eluent) to afford the title compound (87 mg, 46%) as a yellow oil. ν_{max} /cm^{-1} (film) 2941, 1702, 1621, 1588, 1508 and 1357; ^{1}H NMR ($CDCl_3$, 400 MHz): δ 1.89 (1H, qd, *J* 11.6, 10.3, 5.5), 2.07 – 2.18 (1H, m), 2.30 (3H, s), 2.52 (1H, s), 2.59 (1H, d, *J* 11.7), 2.73 – 2.84 (2H, m), 3.12 – 3.29 (2H, m), 3.33 (1H, t, *J* 5.5), 3.49 (1H, s), 3.77 (1H, dd, *J* 11.8, 4.2), 4.02 (3H, s), 4.04 (3H, s), 6.06 (1H, t, *J* 7.3), 6.20 (1H, t, *J* 7.4), 6.77 (1H, d, *J* 4.7), 7.19 (1H, s), 7.42 (1H, s) and 8.56 (1H, d, *J* 4.7); ^{13}C{^{1}H} NMR ($CDCl_3$, 101 MHz): δ 25.2, 28.1, 40.7, 42.4, 43.9, 45.7, 46.9, 55.9, 56.2, 57.8, 64.6, 85.5, 100.7, 109.1, 119.6, 122.3, 128.3, 136.1, 145.4, 147.6, 148.1, 149.7, 151.8 and 211.7; HRMS (ESI$^+$-Orbitrap) m/z: $[M+H]^+$ calcd for $C_{24}H_{26}N_2O_3$ 391.2023; found 391.2013.

Quinoline **7ab**

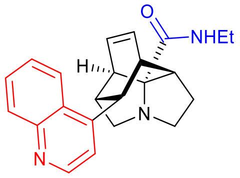

Following General Procedure 1 with aziridine **3b** (93 mg, 0.485 mmol) and allylic acetate **8a** (123 mg, 0.541 mmol). Purified by silica gel chromatography (EtOH.NH_3/Et_2O as eluent) to afford the title compound (113 mg, 65%) as a yellow oil. ν_{max} /cm^{-1} (film) 3338, 2937, 2872, 1654, 1587 and 1376; ^{1}H NMR ($CDCl_3$, 400 MHz): δ 1.15 (3H, t, *J* = 7.3), 1.95 – 2.06 (1H, m), 2.16 (1H, dq, *J* = 15.1, 6.8), 2.43 – 2.55 (2H, m), 2.73 – 2.83 (2H, m), 3.12 (1H, t, J 4.9), 3.19 – 3.31 (3H, m), 6.06 (1H, t, J 7.3), 6.38 (1H, t, J = 7.5), 6.91 (1H, d, J 4.6), 7.58 (1H, t, J 7.7), 7.70 (1H, t, J 7.8), 7.77 – 7.85 (1H, m), 8.02 (1H, d, J 8.6), 8.11 (1H, d, J 8.6) and 8.75 (1H, d, J 4.6); ^{13}C{^{1}H} NMR ($CDCl_3$, 101 MHz): δ 15.1, 28.2, 34.0, 41.5, 41.8, 44.6, 47.8, 48.3, 57.5, 63.6, 80.8, 121.4, 122.6, 126.6, 127.2, 128.9, 129.3, 130.8,

134.6, 148.3, 149.7, 150.2 and 174.5; HRMS (ESI$^+$-Orbitrap) m/z: $[M+H]^+$ calcd for $C_{23}H_{25}N_3O$ 360.2077; found 360.2069.

Quinoline **7bb**

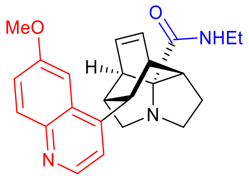

Following General Procedure 1 with aziridine **3b** (93 mg, 0.485 mmol) and allylic acetate **8b** (139 mg, 0.541 mmol). Purified by silica gel chromatography ($EtOH.NH_3/Et_2O$ as eluent) to afford the title compound (130 mg, 69%) as a yellow oil. ν_{max} /cm^{-1} (film) 3249, 2929, 1647, 1625, 1594, 1508 and 1225; ^{1}H NMR ($CDCl_3$, 400 MHz): δ 1.86 – 1.99 (1H, m), 2.10 – 2.22 (1H, m), 2.41- 2.48 (1H, m), 2.52 (1H, d, *J* 11.6), 2.76 (1H, d, *J* 8.4), 2.80 – 2.85 (1H, m), 3.11 (1H, t, *J* 5.4), 3.19 – 3.31 (4H, m), 3.51 (1H, s), 3.65 (1H, dd, *J* 11.6, 4.0), 3.95 (3H, s), 6.06 (1H, t, J 7.2), 6.36 (1H, t, J 7.2), 6.86 (1H, d, J 4.6), 7.35 (1H, dd, J 9.5, 2.7), 7.80 (1H, m), 8.01 (1H, dd, J 9.2, 1.4) and 8.54 – 8.66 (1H, d, J 4.5); ^{13}C{^{1}H} NMR ($CDCl_3$, 101 MHz): δ 15.1, 28.2, 34.0, 40.9, 42.0, 44.5, 47.7, 48.4, 55.5, 57.5, 63.7, 80.8, 101.5, 120.6, 121.4, 128.0, 129.2, 132.2, 134.6, 144.2, 147.3, 148.5, 157.7 and 174.5; HRMS (ESI$^+$-Orbitrap) m/z: $[M+H]^+$ calcd for $C_{24}H_{27}N_3O_2$ 390.2183; found 390.2172.

Quinoline **7cb**

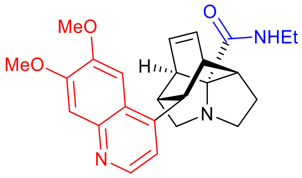

Following General Procedure 1 with aziridine **3b** (93 mg, 0.485 mmol) and allylic acetate **8c** (155 mg, 0.541 mmol). Purified by silica gel chromatography ($EtOH.NH_3/Et_2O$ as eluent) to afford the title compound (134 mg, 67%) as a yellow oil. ν_{max} /cm^{-1} (film) 3338, 2932, 1659, 1621, 1587, 1433 and 1237; ^{1}H NMR ($CDCl_3$, 400 MHz): δ 1.12 (1H, t, *J* 7.3), 1.89 (1H, ddd, *J* 13.4, 8.9, 3.7), 2.08 – 2.19 (1H, m), 2.40 – 2.45 (1H, m), 2.50 (1H, d, *J* 11.7), 2.70 – 2.81 (2H, m), 3.09 (1H, t, *J* 5.3), 3.17 – 3.28 (4H, m), 3.45 (1H, s), 3.62 (1H, dd, *J* 11.8, 4.1), 3.99 (3H, s), 4.01 (3H, s), 6.04 (1H, t, *J* 7.3), 6.33 (1H, t, *J* 7.4), 6.75 (1H, d, *J* 4.6), 7.17 (1H, s), 7.39 (1H, s), 7.78 (1H, t, *J* 5.9) and 8.52 (1H, d, *J* 4.7); ^{13}C{^{1}H} NMR ($CDCl_3$, 101 MHz): δ 15.0, 28.2, 34.0, 41.0, 42.1, 44.3, 47.7, 48.4, 55.9, 56.2, 57.5, 63.7, 80.7, 100.7, 109.1, 119.7, 122.3, 129.2, 134.5, 145.4, 147.6, 148.2, 149.6, 151.7 and 174.4; HRMS (ESI$^+$-Orbitrap) m/z: $[M+H]^+$ calcd for $C_{25}H_{29}N_3O_3$ 420.2288; found 420.2277.

Quinoline **7ac**

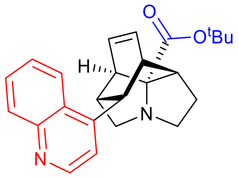

Following General Procedure 1 with aziridine **3c** (96 mg, 0.434 mmol) and allylic acetate **8a** (118 mg, 0.519 mmol). Purified by silica gel chromatography ($EtOH.NH_3/Et_2O$ as eluent) to afford the title compound (83 mg, 50%) as a yellow oil. ν_{max} /cm^{-1} (film) 2963, 1722, 1587, 1568 and 1165; ^{1}H NMR ($CDCl_3$, 400 MHz): δ 1.45 (9H, s), 1.96 – 2.07 (1H, m), 2.22 – 2.33 (1H, m), 2.38 – 2.45 (2H, m), 2.49 (1H, d, *J* 11.8), 2.68 – 2.75 (1H, m), 3.28 (1H, t, J 10.7), 3.51 (1H, t, *J* 5.5), 3.54 – 3.64 (2H, m), 3.69 (1H, dd, *J* 11.9, 4.2), 5.96 (1H, t, *J* 7.3), 6.39 (1H, t, *J* 7.3), 6.92 (1H, d, *J* 4.5), 7.58 (1H, t, *J* 7.7), 7.69

(1H, t, *J* 7.7), 8.01 (1H, d, *J* 8.3), 8.10 (1H, d, *J* 8.5) and 8.74 (1H, d, *J* 4.5); $^{13}C\{^1H\}$ NMR ($CDCl_3$, 101 MHz): δ 28.1, 28.3, 41.3, 41.8, 43.3, 45.4, 50.1, 59.0, 64.7, 80.9, 81.1, 121.3, 122.6, 126.6, 127.2, 128.9, 130.3, 130.8, 134.5, 148.2, 149.7, 150.1 and 173.6; HRMS (ESI^+-Orbitrap) m/z: $[M+H]^+$ calcd for $C_{25}H_{28}N_2O_2$ 389.2230; found 389.2222.

Quinoline **7bc**

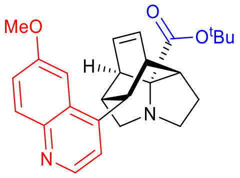

Following General Procedure 1 with aziridine **3c** (96 mg, 0.434 mmol) and allylic acetate **8b** (134 mg, 0.521 mmol). Purified by silica gel chromatography ($EtOH.NH_3/Et_2O$ as eluent) to afford the title compound (103mg, 57%) as a yellow oil. ν_{max} /cm^{-1} (film) 2947, 2869, 1722, 1621, 1591, 1508, 1369 and 1225; ^1H NMR ($CDCl_3$, 400 MHz): δ 1.45 (9H, s), 1.97 (1H, ddt, *J* 13.9, 9.3, 4.3), 2.28 (1H, qd, *J* 11.3, 9.9, 3.1), 2.38 – 2.46 (2H, m), 2.51 (1H, d, *J* 11.8), 2.75 (1H, d, *J* 6.5), 3.28 (1H, t, J 10.7), 3.42 – 3.53 (2H, m), 3.59 (1H, dt, *J* 13.2, 9.0), 3.70 (1H, dd, *J* 11.7, 4.2), 3.95 (3H, s), 5.98 (1H, t, *J* 7.3), 6.38 (1H, t, *J* 7.4), 6.88 (1H, d, *J* 4.5), 7.35 (1H, d, *J* 9.3), 8.00 (1H, d, *J* 9.2) and 8.61 (1H, d, *J* 4.5); $^{13}C\{^1H\}$ NMR ($CDCl_3$, 101 MHz): δ 28.1, 28.4, 40.7, 42.0, 43.2, 45.4, 50.0, 55.5, 59.1, 64.8, 80.8, 81.1, 101.5, 120.7, 121.3, 128.0, 130.3, 132.2, 134.5, 144.3, 147.4, 148.5, 157.8 and 173.6; HRMS (ESI^+-Orbitrap) m/z: $[M+H]^+$ calcd for $C_{26}H_{30}N_2O_3$ 419.2336; found 419.2327.

Quinoline **7cc**

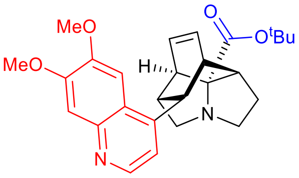

Following General Procedure 1 with aziridine **3c** (96 mg, 0.434 mmol) and allylic acetate **8c** (149 mg, 0.520 mmol). Purified by silica gel chromatography ($EtOH.NH_3/Et_2O$ as eluent) to afford the title compound (86 mg, 44%) as a yellow oil. ν_{max} /cm^{-1} (film) 2932, 1722, 1621, 1587, 1508 and 1237; ^1H NMR ($CDCl_3$, 400 MHz): δ 1.44 (9H, s), 1.92 (1H, ddt, *J* 17.1, 12.6, 5.9), 2.28 (1H, ddd, *J* 18.7, 9.1, 4.4), 2.38 – 2.44 (2H, m), 2.50 (1H, d, *J* 11.5), 2.69 – 2.74 (1H, m), 3.27 (1H, t, J 10.6), 3.41 – 3.51 (2H, m), 3.52 – 3.63 (1H, m), 3.64 – 3.73 (2H, m), 4.01 (3H, s), 4.02 (3H, s), 5.98 (1H, t, *J* 7.3), 6.36 (1H, t, *J* 7.3), 6.79 (1H, d, *J* 4.7), 7.18 (1H, s), 7.40 (1H, s) and 8.55 (1 H, d, *J* 4.7); $^{13}C\{^1H\}$ NMR ($CDCl_3$, 101 MHz): δ 28.1, 28.4, 40.9, 42.2, 43.1, 45.5, 50.0, 55.9, 56.2, 59.1, 64.8, 80.8, 81.0, 100.7, 109.1, 119.6, 122.3, 130.2, 134.5, 145.4, 147.6, 148.3, 149.6, 151.7 and 173.7; HRMS (ESI^+-Orbitrap) m/z: $[M+H]^+$ calcd for $C_{27}H_{32}N_2O_4$ 449.2442; found 449.2434.

Quinoline **7da**

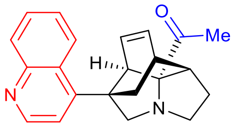

Following a modified General Procedure 1 with aziridine **3a** (100 mg, 0.613 mmol) and allylic acetate **9** (140 mg, 0.617 mmol). Allylic acetate **15** was only added after the reaction had spent 20 min at 50 °C before heating to reflux. Purification by silica gel chromatography ($EtOH.NH_3/Et_2O$ as eluent) afforded the title compound (5.8 mg, 3%) as a yellow oil. ν_{max} /cm^{-1} (film) 2927, 2865, 1702, 1584, 1509 and 1353; ^1H NMR ($CDCl_3$, 400 MHz): δ 1.58 (1H, d, J 13.2), 1.79 (1H, dtd, *J* 13.0, 9.0, 3.7), 2.02 – 2.12

(1H, m), 2.30 (3H, s), 2.52 – 2.60 (2H, m), 2.72 (1H, dq, J 9.4, 2.7), 3.16 – 3.26 (2H, m), 3.29 (1H, d, *J* 11.6), 3.43 (1H, d, *J* 11.5), 3.65 (d, *J* = 5.9), 6.31 (1H, dd, *J* 8.0, 6.3), 6.51 (1H, t, *J* 7.5), 7.20 (1H, d, *J* 4.6), 7.54 (1H, ddd, *J* 8.4, 6.8, 1.4), 7.69 (1H, ddd, *J* 8.6, 7.0, 1.3), 8.06 (1H, d, *J* 8.6), 8.12 (1H, dd, *J* 8.4, 1.4) and 8.81 (1H, d, *J* 4.6); $^{13}C\{^{1}H\}$ NMR ($CDCl_3$, 101 MHz): δ 25.3, 28.5, 36.0, 38.3, 46.2, 49.1, 51.3, 58.1, 69.3, 85.2, 118.2, 125.6, 126.1, 127.1, 127.5, 128.9, 130.9, 138.2, 149.2, 150.4, 152.2 and 211.6; HRMS (ESI^+-Orbitrap) m/z: $[M+H]^+$ calcd for $C_{22}H_{22}N_2O$ 331.1811; found 331.1806.

Quinoline **7db**

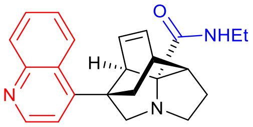

Following General Procedure 1 with aziridine **3b** (93 mg, 0.484 mmol) and allylic acetate **9** (110 mg, 0.568 mmol). Purified by silica gel chromatography ($EtOH.NH_3/Et_2O$ as eluent) to afford the title compound (21 mg, 12%) as a yellow oil. ^{1}H NMR ($CDCl_3$, 400 MHz): δ 1.13 (3H, t, J 6.9), 1.60 (1H, d, *J* 13.0), 1.69 – 1.84 (1H, m), 2.06 (1H, app. p, *J* 7.3), 2.50 – 2.61 (2H, m), 2.77 (1H, d, *J* 6.8), 3.16 – 3.36 (6H, m), 3.45 (1H, d, *J* 6.2), 6.45 (1H, t, *J* 7.2), 6.53 (1H, t, *J* 7.2), 7.16 (1H, d, *J* 4.7), 7.53 (1H, t, *J* 7.6), 7.68 (1H, t, *J* 7.6), 7.76 (1H, s), 8.05 (1H, d, *J* 8.6), 8.11 (1H, d, *J* 8.2) and 8.79 (1H, d, *J* 4.6); $^{13}C\{^{1}H\}$ NMR ($CDCl_3$, 101 MHz): δ 15.0, 28.5, 34.0, 36.2, 38.3, 47.7, 50.8, 51.6, 57.6, 68.5, 80.5, 118.3, 125.6, 126.0, 127.4, 127.8, 128.8, 130.9, 136.8, 149.3, 150.5, 152.1 and 174.6; HRMS (ESI^+-Orbitrap) m/z: $[M+H]^+$ calcd for $C_{23}H_{25}N_3O$ 360.2077; found found 360.2071.

Quinoline **7dc**

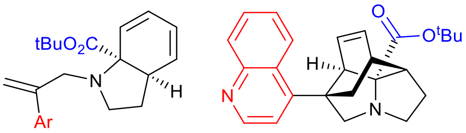

Following a modified General Procedure 1 with aziridine **3c** (120 mg, 0.543 mmol) and allylic acetate **9** (132 mg, 0.576 mmol). Allylic acetate **15** was added after the reaction had been heated to 70 °C for 20 min and the reaction was then maintained at 100 °C for 64 h. Purified by silica gel chromatography ($EtOH.NH_3/Et_2O$, 1% to 10% as eluent) to yield N-allylated diene **S9** (40 mg) and the title compound **3dc** (15 mg), both as clear oils. Diene **S9** was dissolved in degassed xylene (20 mL) and heated to 140 °C under argon. After 15 h the reaction was cooled to rt, evaporated and purified by silica gel chromatography ($EtOH/NH_3$ in Et_2O, 1% to 10% as eluent) to afford further **3dc** (23 mg) as a clear oil. Total yield: 38 mg, 18%. ν_{max} /cm^{-1} (film) 2968, 1723, 1587, 1508 and 1162; ^{1}H NMR ($CDCl_3$, 400 MHz): δ 1.47 (9H, s), 1.55 – 1.62 (1H, m), 1.81 (1H, dtd, J 13.1, 9.0, 3.3), 2.20 (1H, qd, J 9.6, 4.8), 2.39 – 2.45 (1H, m), 2.47 – 2.53 (2H, m), 3.20 (1H, d, J 11.6), 3.26 (1H, ddd, J 12.7, 9.3, 3.3), 3.36 (1H, d, J 11.6), 3.53 – 3.62 (1H, m), 3.85 (1H, d, J 6.1), 6.39 – 6.50 (2H, m), 7.51 (1H ddd, J 8.4, 6.8, 1.4), 7.66 (1H, ddd, J 8.3, 6.8, 1.3), 8.03 (1H, d, J 8.5), 8.10 (1H, dd, J 8.5, 1.4) and 8.80 (d, J 4.6); $^{13}C\{^{1}H\}$ NMR ($CDCl_3$, 101 MHz): δ 28.1, 28.8, 36.2, 38.1, 47.9, 50.4, 50.6, 59.3, 69.5, 80.6, 81.1, 118.4, 125.7, 126.0, 127.5, 128.8, 128.9, 130.9, 136.7, 149.3, 150.5, 152.1 and 173.8; HRMS (ESI^+-Orbitrap) m/z: $[M+H]^+$ calcd for $C_{25}H_{29}N_2O_2$ 389.2229; found 389.2222.

Larger Scale Reactions

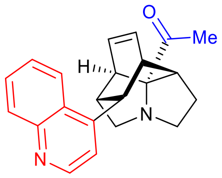

To a dried Schlenk tube under argon were added palladium(II) acetate (46.6 mg, 0.208 mmol), triphenylphosphine (218.7 mg, 0.834 mmol), potassium carbonate (348.3 mg, 2.52 mmol) and dry dioxane (12 mL). The mixture was sparged with argon, stirred for 5 min and a solution of aziridine **3a** (320 mg, 1.96 mmol) and allylic acetate **8a** (535 mg, 2.35 mmol) in dry dioxane (12 mL) was added. The mixture was sparged with argon and heated to reflux. After 15 h the reaction was cooled, and passed through Celite, eluting with EtOAc. The filtrate was concentrated and the residue purified by silica gel chromatography (EtOH.NH_3/DCM as eluent) to afford the title compound (356 mg, 55%) as a yellow oil.

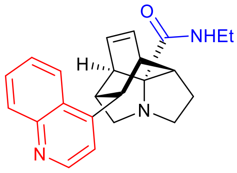

To a dried Schlenk tube under argon were added palladium(II) acetate (29.1 mg, 0.130 mmol), triphenylphosphine (136.5 mg, 0.520 mmol), potassium carbonate (217 mg, 1.57 mmol) and dry dioxane (10 mL). The mixture was sparged with argon, stirred for 5 min and a solution of aziridine **3b** (235 mg, 1.22 mmol) and allylic acetate **8a** (306 mg, 1.34 mmol) in dry dioxane (10 mL) was added. The mixture was sparged with argon and heated to reflux. After 15 h the reaction was cooled, and passed through Celite, eluting with EtOAc. The filtrate was concentrated and the residue purified by silica gel chromatography (EtOH.NH_3/Et_2O as eluent) to afford the title compound (351 mg, 80%) as a yellow solid.

6. Photophysical Studies

The photophysical analysis was performed using a Shimadzu UV-2600 for the UV-Vis absorption spectra and a HORIBA Fluorolog-QM for the photoluminescence emission and excitation spectra. The films were made by dropcasting a mixture of the compound and the polymer in question at the stated weight percentage on to a sapphire substrate. Photoluminescence quantum yields (PLQYs) were obtained using an integrating sphere in conjunction with the Fluorolog-QM with neutral density filters to correct for high intensity excitation. The PLQYs were calculated using the mathematical formula included within the HORIBA FelixFl software.

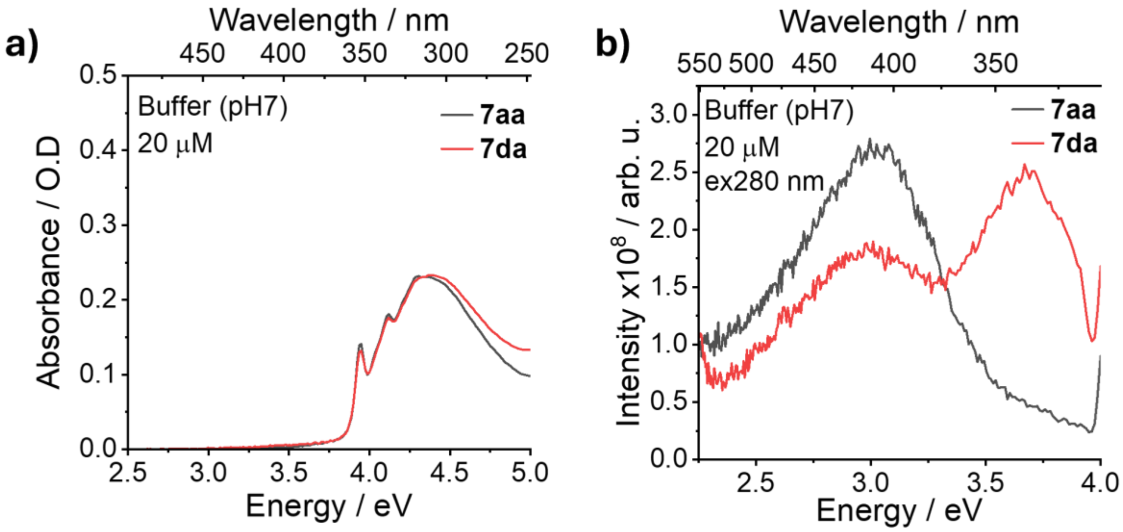

Figure S1: a) The absorption and b) photoluminescence of **7aa** and **7da** in pH7 aqueous phosphate buffer. This shows the complex nature of interpreting the equilibrium species. For **7aa** we observe very weak photoluminescence from the doubly protonated species and for the **7da** there is a mixture of the locally excited emission of the singly protonated species and some remaining emission from the doubly protonated species.

Further to the discussion in the main manuscript, the difference in PLQY between the doubly protonated and that of the neutral and singly protonated species of these systems means that at intermediate pHs such as 7 there will always be competing emission pathways and emissive species. This complexity means that only MeCN and 0.1 M H_2SO_4 are used as solvents for the main studies.

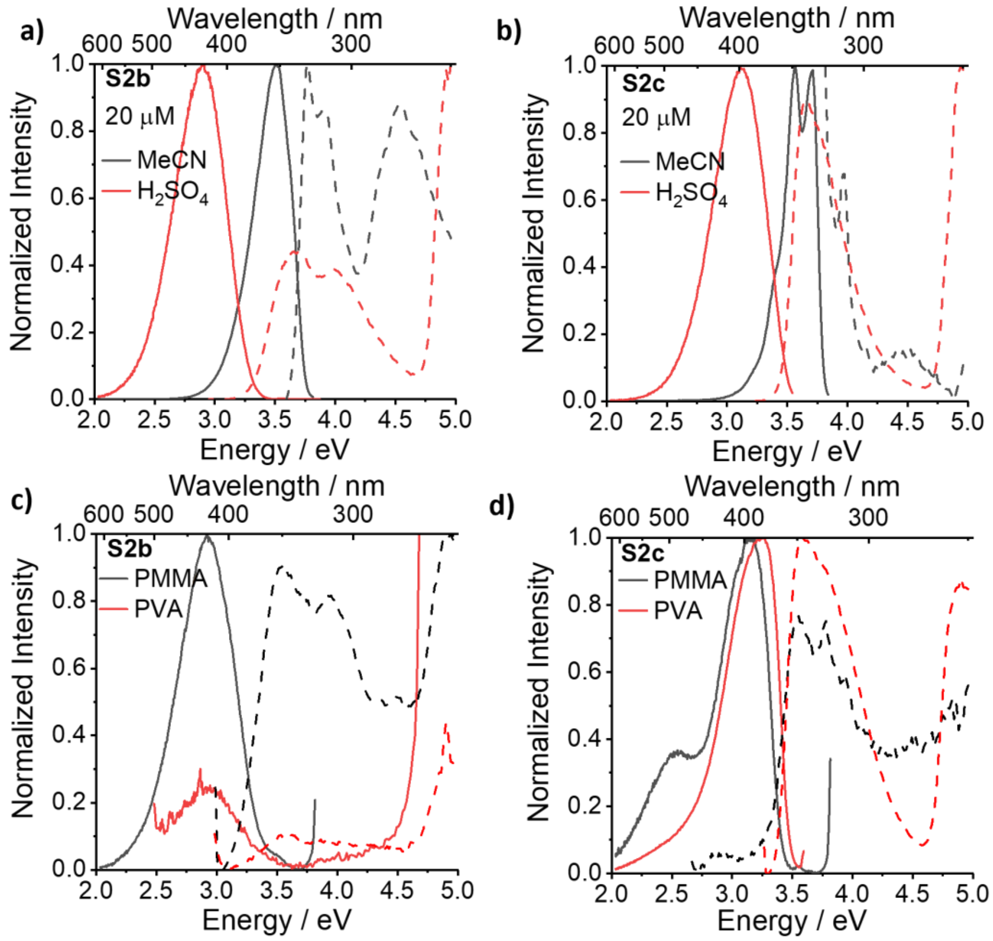

Figure S2: The excitation (dashed) and emission (solid) profiles of the compounds (**S2b** and **S2c**) in both solution (MeCN and 0.1 M H_2SO_4) and dropcast film (PMMA and PVA) at 1 wt%. Peak value for excitation (Peak) and excitation wavelength for emission (Ex) in the form (Peak/Ex) as follows: a) MeCN (350/315 nm) and 0.1 M H_2SO_4 (430/310 nm); b) MeCN (340/315 nm) and 0.1 M H_2SO_4 (400/340 nm); c) PMMA (425/315 nm) and PVA (420/280 nm); and d) PMMA (395/315 nm) and PVA (380/345 nm).

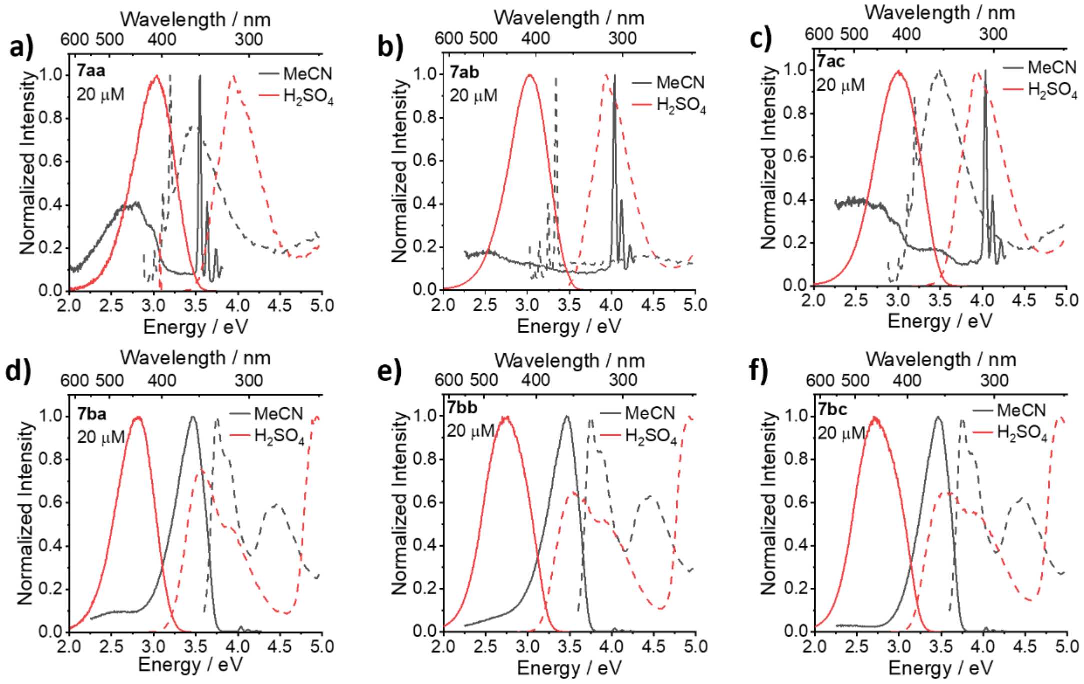

Figure S3: The excitation (dashed) and emission (solid) profiles of the compounds (**7aa** to **7bc**) in both MeCN and 0.1 M H_2SO_4 at 20 μM. Peak value for emission (Peak) and excitation wavelength for emission (Ex) in the form (Peak/Ex) as follows: a) MeCN (455/315 nm) and 0.1 M H_2SO_4 (410/315 nm); b) MeCN (490/280 nm) and 0.1 M H_2SO_4 (410/315 nm); c) MeCN (485/280 nm) and 0.1 M H_2SO_4 (410/315 nm); d) MeCN (355/280 nm) and 0.1 M H_2SO_4 (440/345 nm); e) MeCN (355/280 nm) and 0.1 M H_2SO_4 (450/345 nm); and f) MeCN (360/280 nm) and 0.1 M H_2SO_4 (455/345 nm). The sharp peaks in the **7ax** series MeCN data are Raman scatter from the solvent due to the low intensity emission from the molecules.

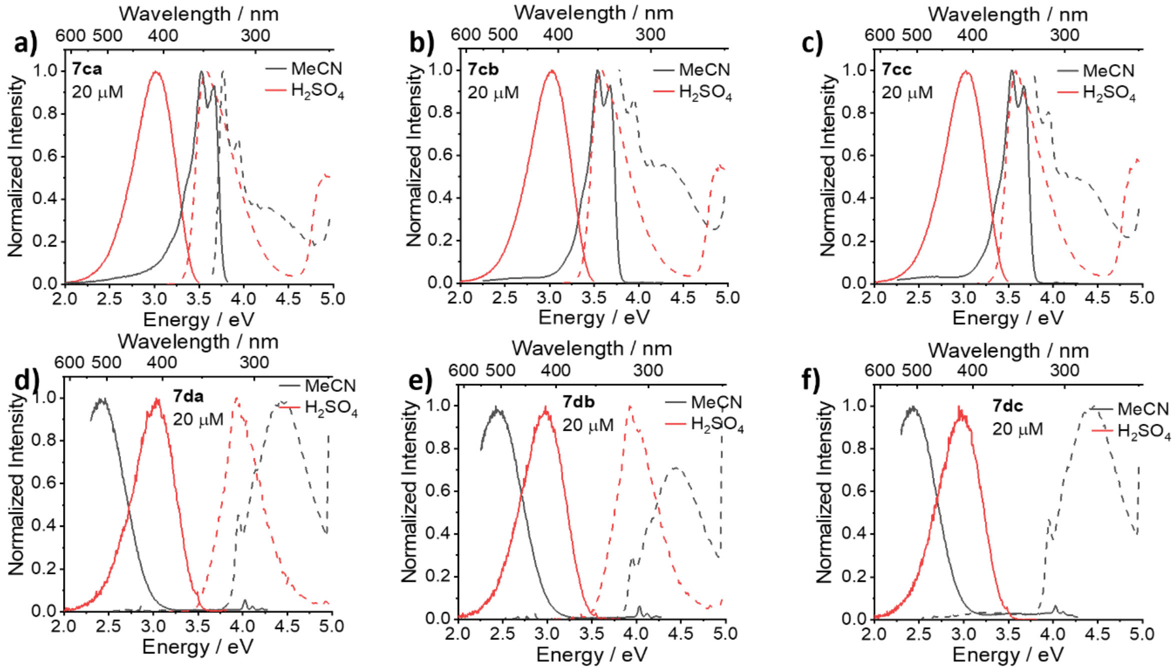

Figure S4: The excitation (dashed) and emission (solid) profiles of the compounds (**7ca** to **7dc**) in both 0.1 M H_2SO_4 and MeCN at 20 μM. Peak value for emission (Peak) and excitation wavelength for emission (Ex) in the form (Peak/Ex) as follows: a) MeCN (350/315 nm) and 0.1 M H_2SO_4 (410/345 nm); b) MeCN (350/280 nm) and 0.1 M H_2SO_4 (410/345 nm); c) MeCN (350/280 nm) and 0.1 M H_2SO_4

(410/345 nm); d) MeCN (510/280 nm) and 0.1 M H_2SO_4 (405/315 nm); e) MeCN (510/280 nm) and 0.1 M H_2SO_4 (415/315 nm); and f) MeCN (510/280 nm) and 0.1 M H_2SO_4 (415/315 nm).

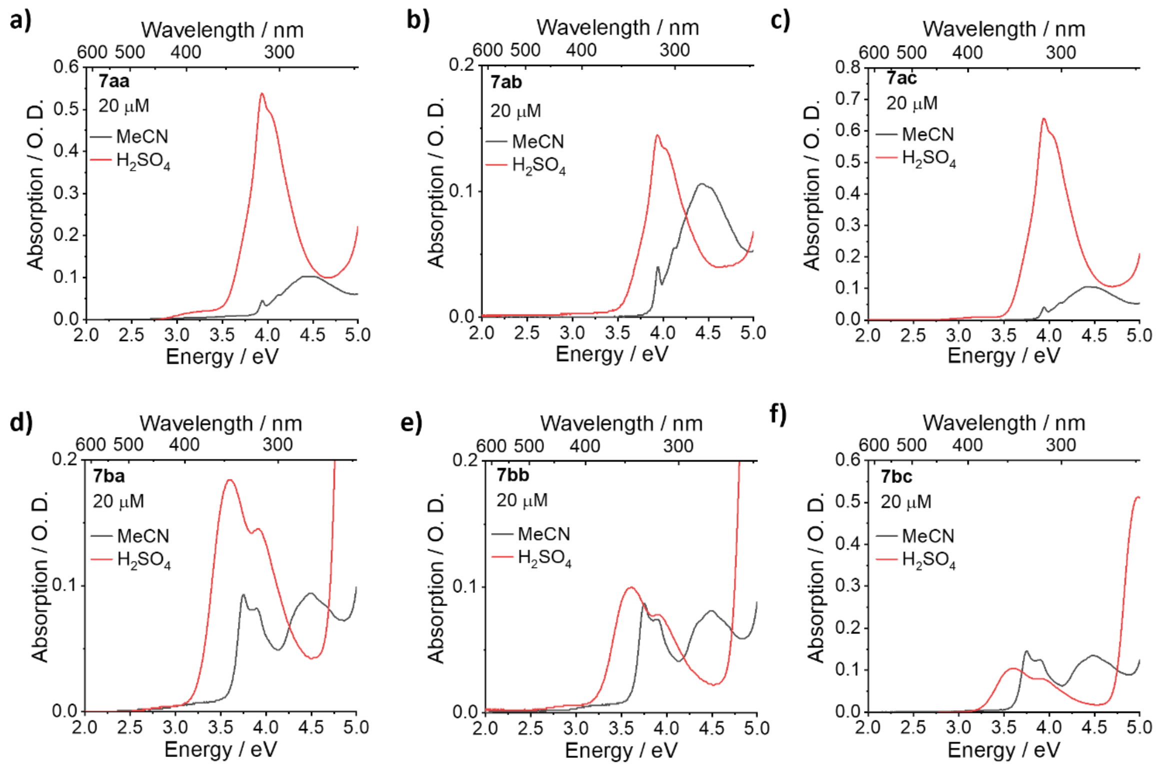

Figure S5: The absorption profiles of the compounds (**7aa** to **7bc**) in both 0.1M H_2SO_4 and MeCN at 20 μM.

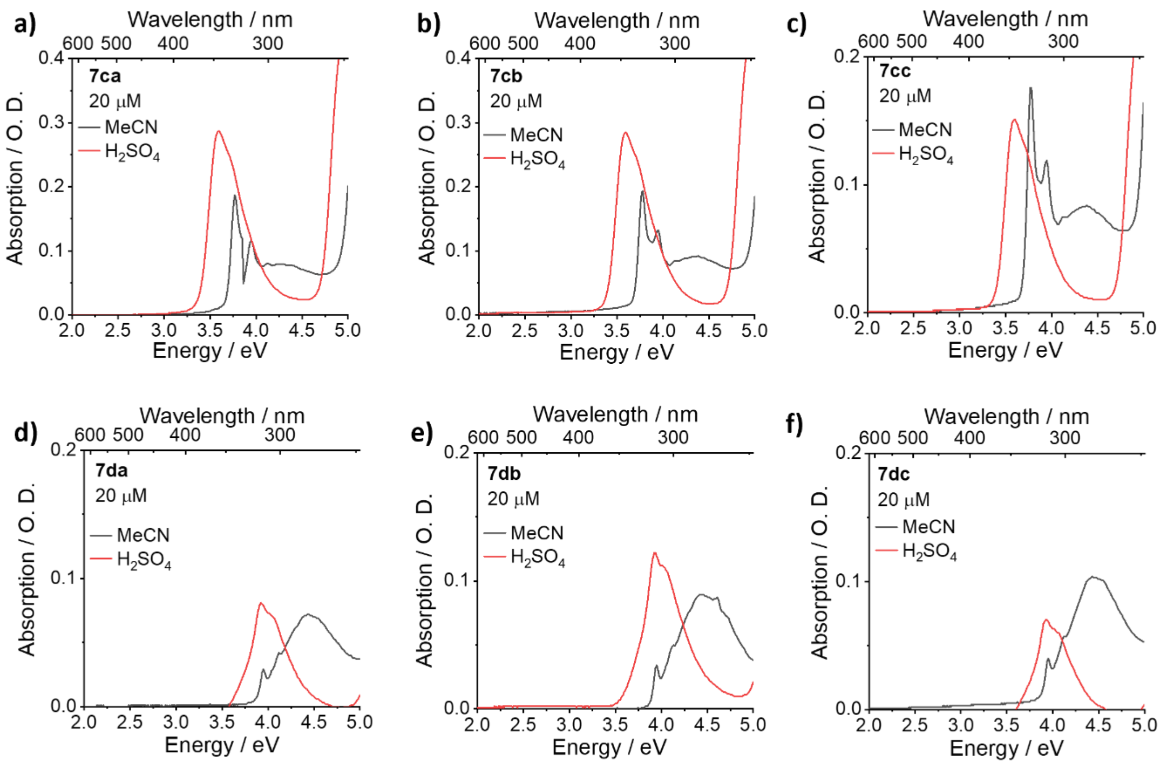

Figure S6: The absorption profiles of the compounds (***7*ca** to ***7*dc**) in both 0.1M H_2SO_4 and MeCN at 20 μM.

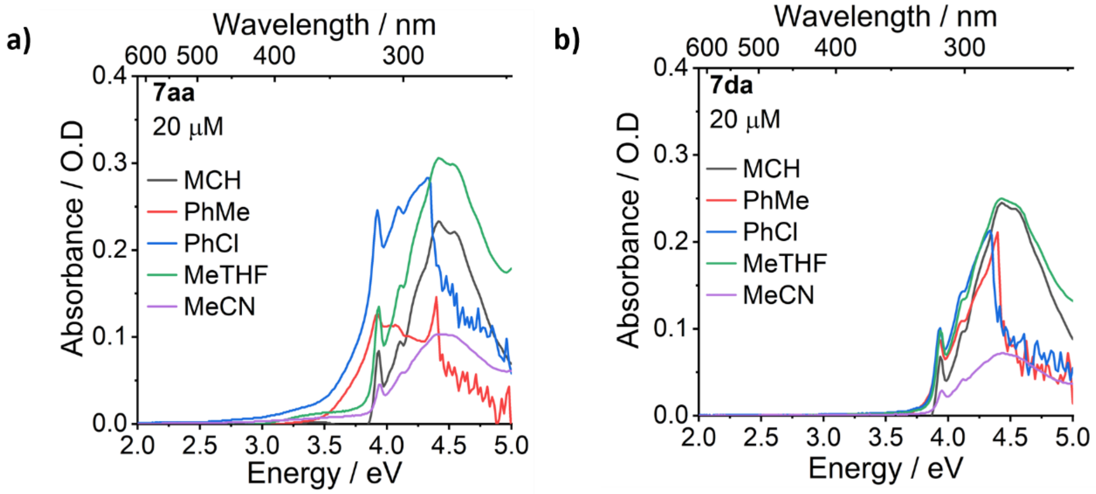

Figure S7: The absorption profiles of the compounds a) **7aa** and b) **7da** in a series of solvents. MCH = methylcyclohexane; MeTHF = 2-methyltetrahydrofuran.

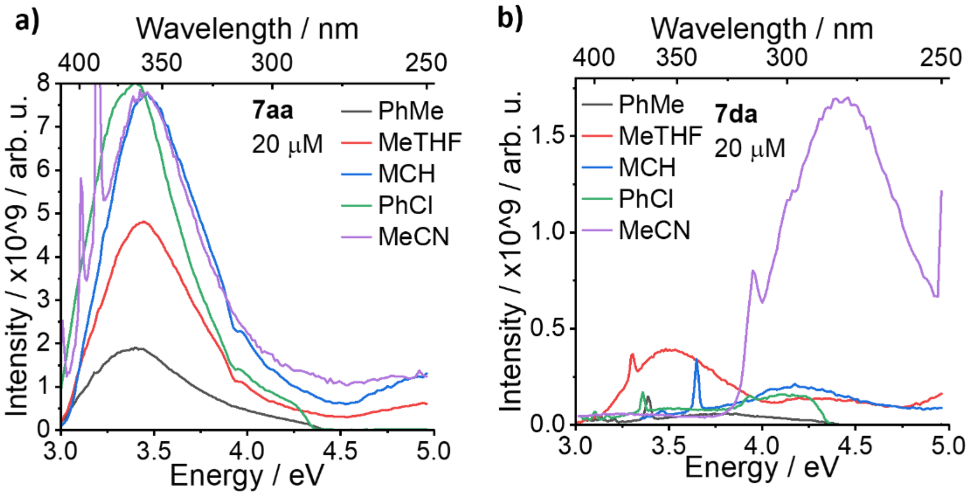

Figure S8: The excitation profiles of the compounds a) **7aa** and b) **7da** in a series of solvents. MCH = methylcyclohexane; 2-MeTHF = 2-methyltetrahydrofuran.

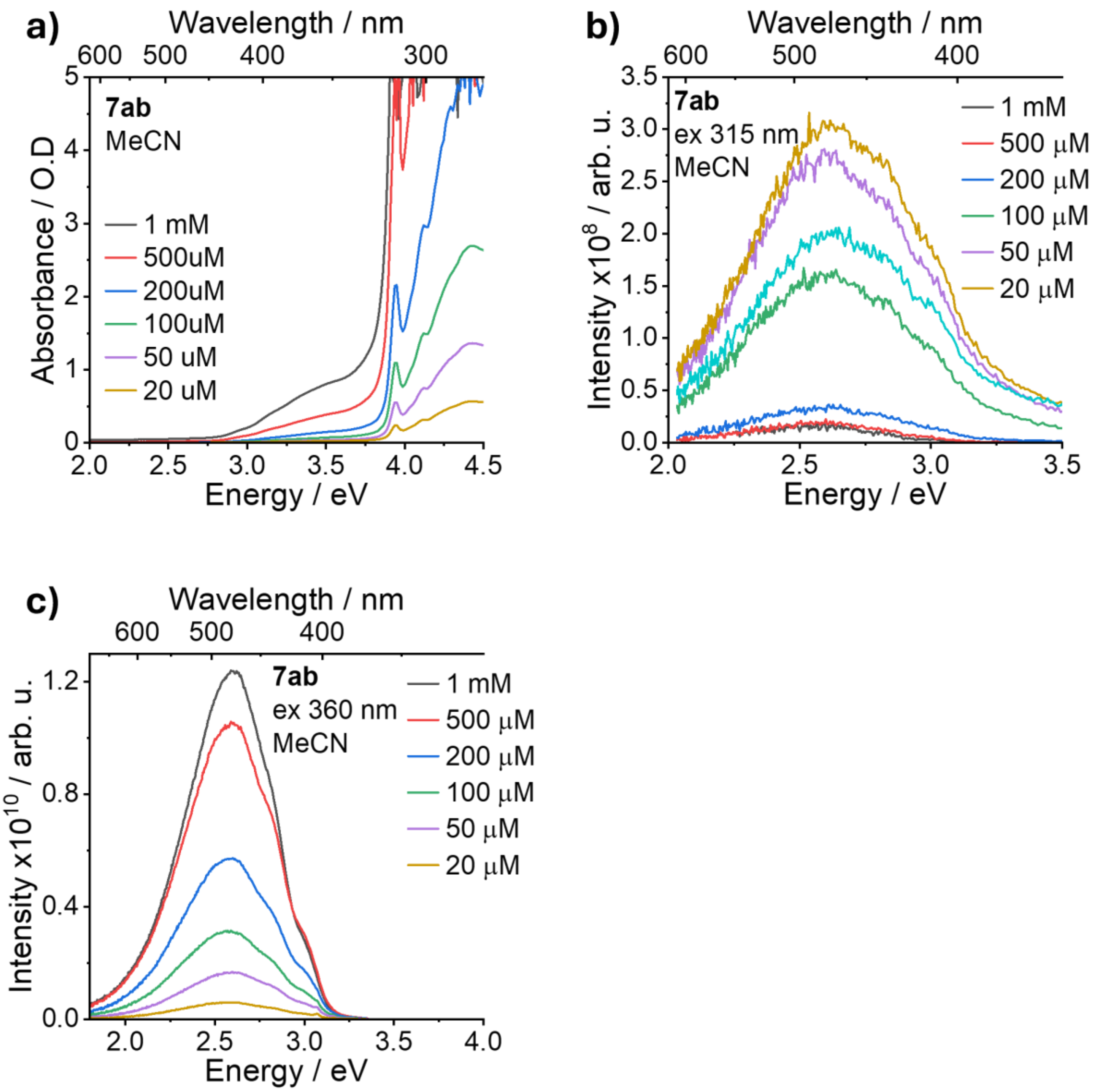

Figure S9: Concentration series of **7ab** in MeCN showing a) the absorption spectra, b) photoluminescence when excited at 315 nm and c) photoluminescence when excited at 360 nm. The absorption shows a new band appearing between 3.0 eV (413 nm) and 3.75 eV (330 nm). The excitation into the new band at 360 nm shows the presence of a red-shifted aggregate. It is this new species, which grows in with increasing concentration, which is attributed to causing a lower PLQY in the high concentration PMMA films.

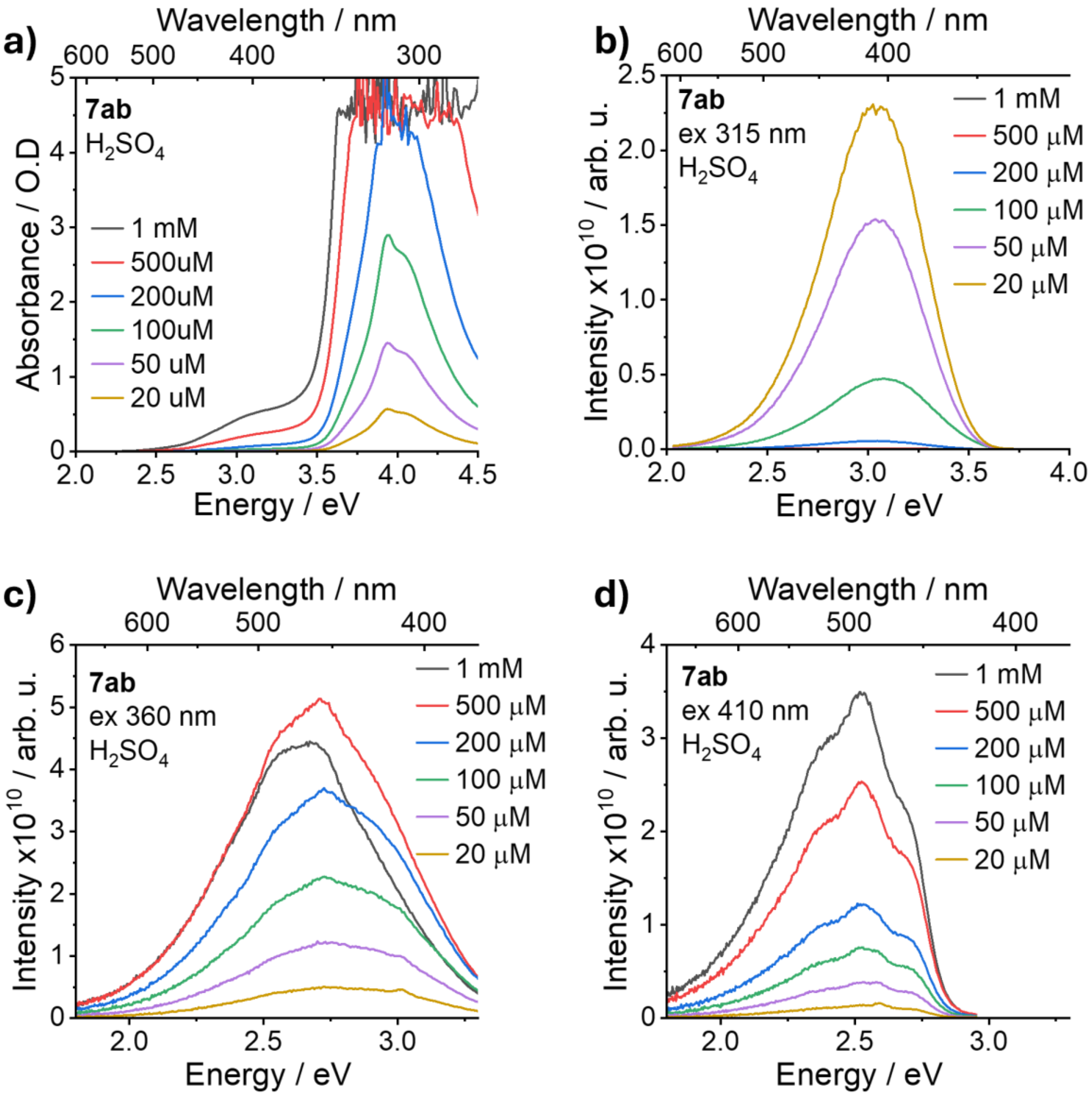

Figure S10: Concentration series of **7ab** in 0.1 M H_2SO_4 showing a) the absorption spectra, b) photoluminescence when excited at 315 nm and c) photoluminescence when excited at 360 nm and d) photoluminescence when excited at 410 nm. The absorption shows a new band appearing between 2.50 eV (496 nm) and 3.50 eV (354 nm). The excitation into the new band at 360 nm and 410 nm shows the presence of a red-shifted aggregate that peaks at 2.50 eV. It grows in at 360 nm excitation with some monomer emission remaining and then dominates completely at 410 nm excitation as the aggregate absorption band is prioritised. It is this new species, which grows in with increasing concentration, which is attributed to causing a lower PLQY in the high concentration PVA films.

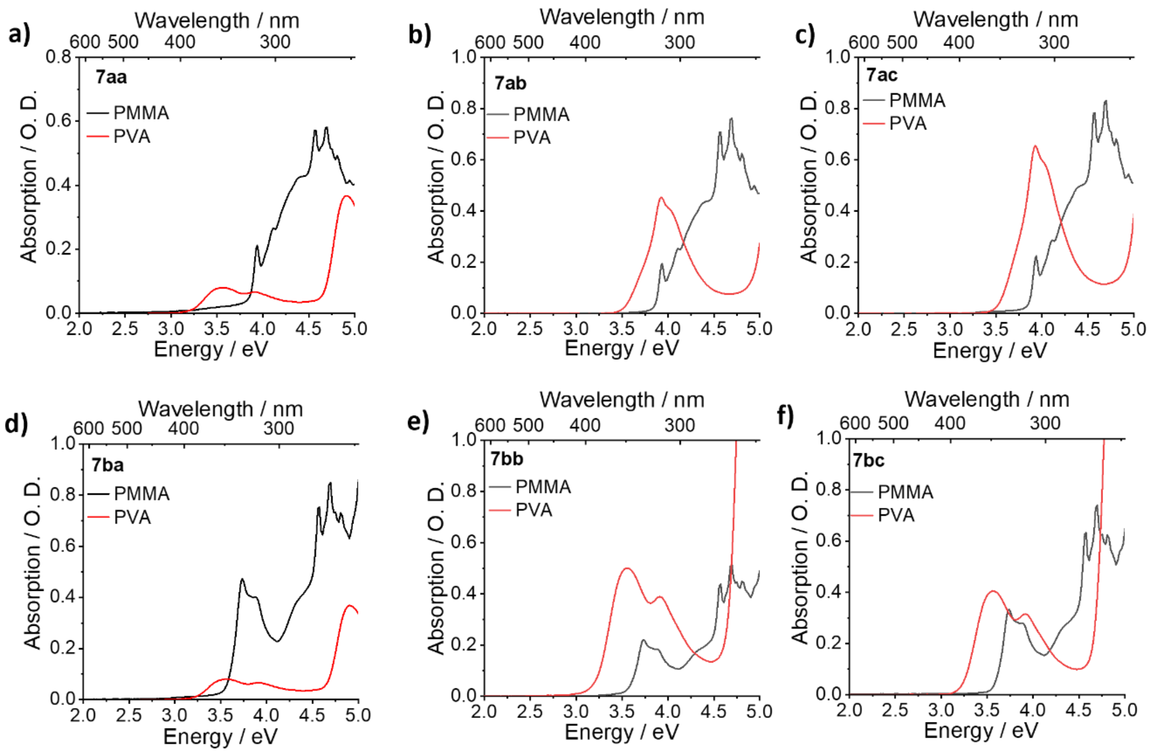

Figure S11: The absorption profiles of the compounds (***7*aa** to ***7*bc**) in both PMMA and PVA at 1 wt%.

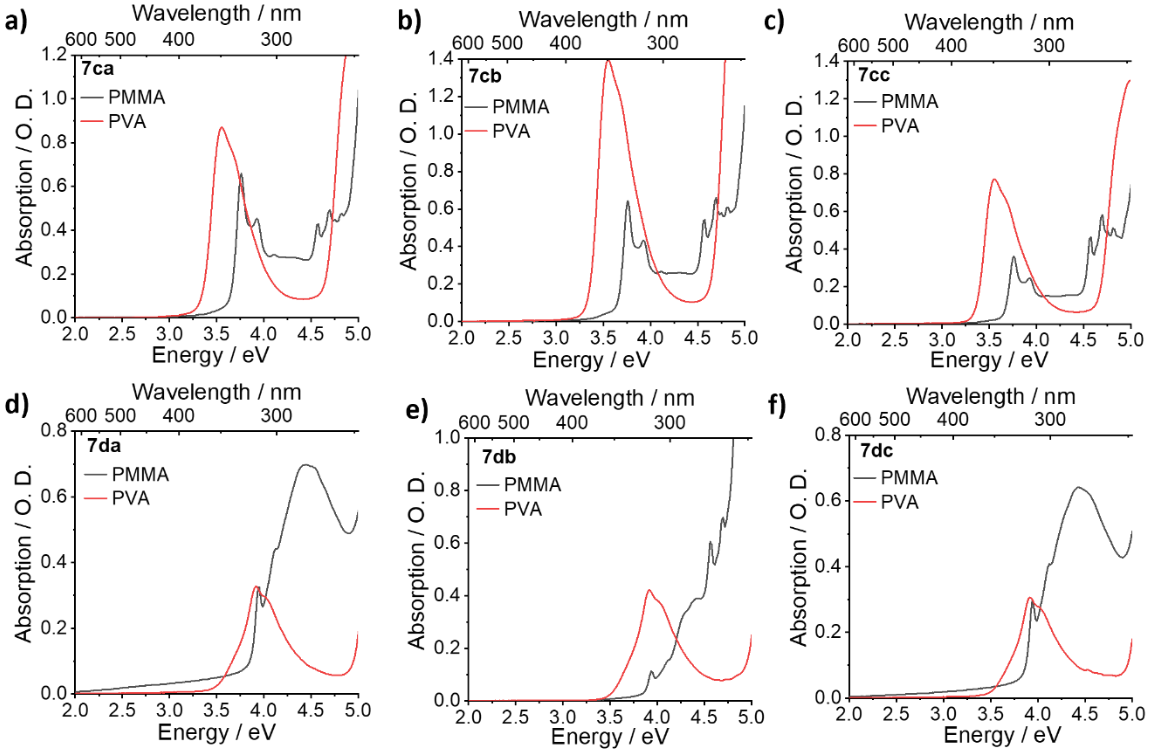

Figure S12: The absorption profiles of the compounds (***7*ca** to ***7*dc**) in both PMMA and PVA at 1 wt%.

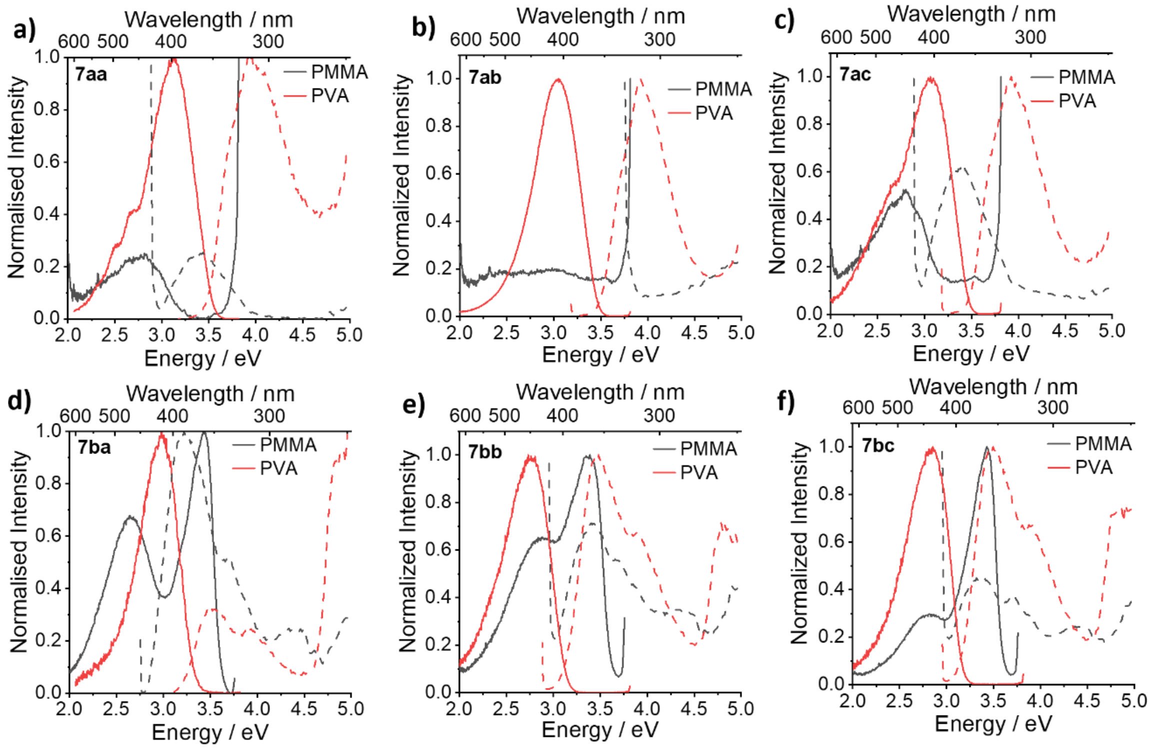

Figure S13: The excitation (dashed) and emission (solid) profiles of the compounds (**7aa** to **7bc**) in both PMMA and PVA at 1 wt%. Peak value for excitation (Peak) and excitation wavelength for emission (Ex) in the form (Peak/Ex) as follows: a) PMMA (440/315 nm) and PVA (400/315 nm); b) PMMA (340/315 nm) and PVA (400/315 nm); c) PMMA (440/315 nm) and PVA (400/315 nm); d) PMMA (460/320 nm) and PVA (420/315 nm); e) PMMA (430/320 nm) and PVA (440/315 nm); and f) PMMA (430/320 nm) and PVA (430/315 nm).

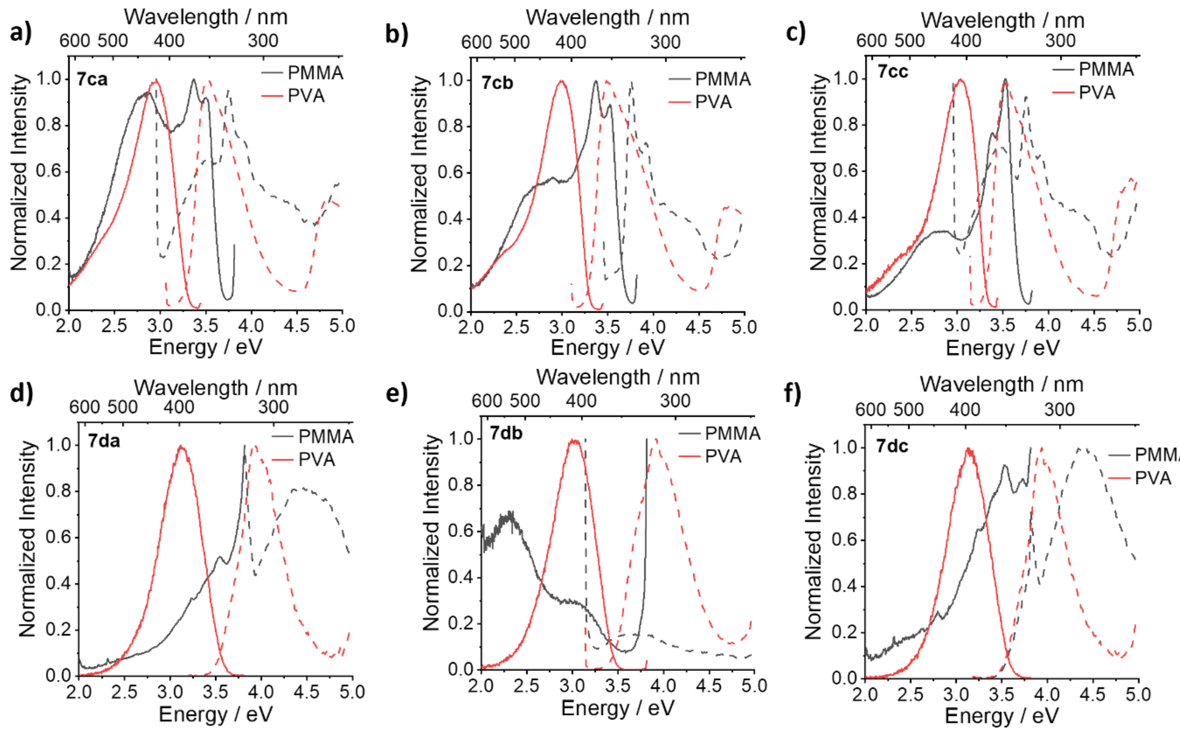

Figure S14: The excitation (dashed) and emission (solid) profiles of the compounds (**7ca** to **7dc**) in both PMMA and PVA at 1 wt%. Peak value for excitation (Peak) and excitation wavelength for emission (Ex) in the form (Peak/Ex) as follows: a) PMMA (430/315 nm) and PVA (415/350 nm); b) PMMA (370/315 nm) and PVA (410/350 nm); c) PMMA (430/315 nm) and PVA (405/350 nm); d) PMMA (400/315 nm) and PVA (400/315 nm); e) PMMA (405/315 nm) and PVA (405/315 nm); and f) PMMA (400/315 nm) and PVA (400/315 nm).

Table S1: PLQY data for tricyclic quinine analogues in 1wt% PVA and PMMA films. The values were obtained from two repeats apart from **7db** which are single measurements.

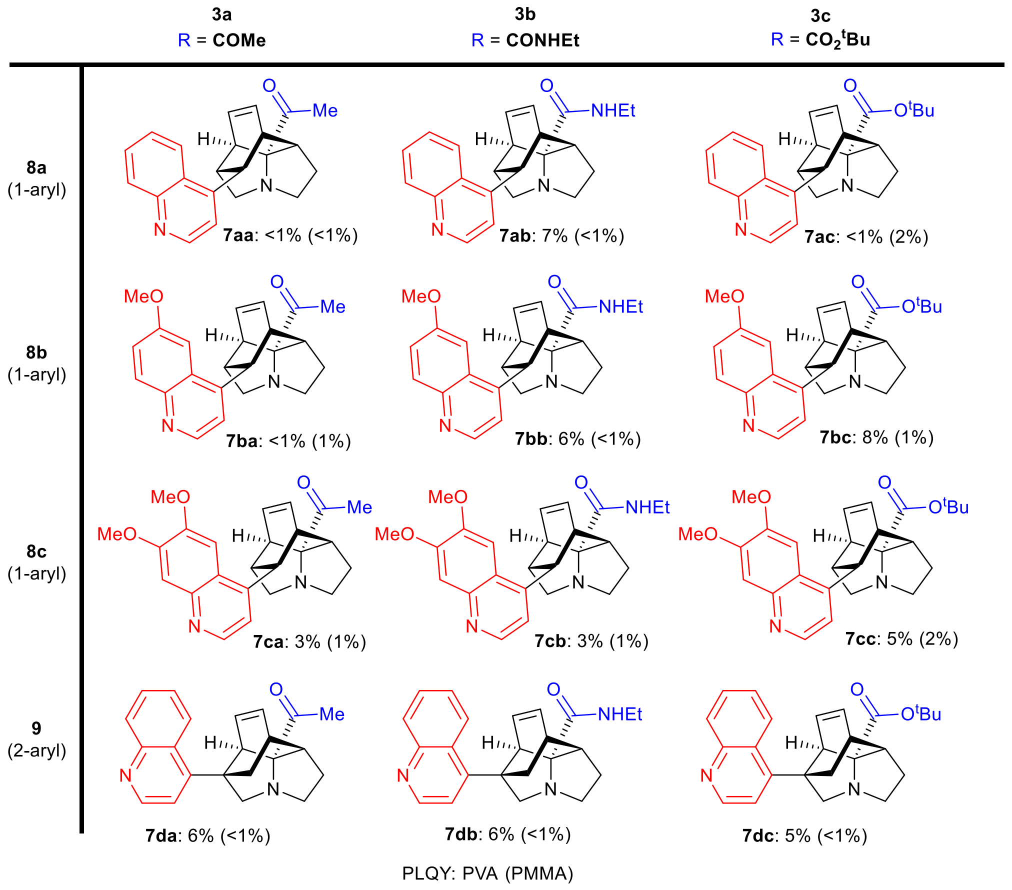

Table S2: PLQY data for monosubstituted quinoline (**S2b**) and disubstituted quinoline (**S2c**) in solution and in film. The values were obtained from two repeats.

	PLQY / %			
Compound	MeCN	0.1M H_2SO_4	PMMA	PVA
**S2b**	5	69	1	1
**S2c**	17	29	1	2

7. Theoretical Calculations

Ground state geometries

Conformer searches on all species were performed using the xtb package, implemented with the Conformer–Rotamer Ensemble Sampling Tool (CREST),[12,13] with default parameters other than an RMSD threshold of 1 Å and an energy threshold of 10 kJ mol^{-1}. A solvent field was used in all cases using the ALPB solvation model.[14] The lowest-energy conformer was then used as a starting point for subsequent DFT calculations. Ground-state optimisations were performed using Gaussian 16.[15]

Table S3: Distances between the N atom on the cage to the point of attachment of the quinoline ring containing the N atom for optimised ground state structures calculated at the ωB97X-D/6-31G(d) level[16]

	N - aryl distance / Å	
	neutral	protonated
**7aa**	4.614	4.556
**7ab**	4.610	4.609
**7ac**	4.627	4.592
**7ba**	4.606	4.563
**7bb**	4.603	4.607
**7bc**	4.615	4.593
**7ca**	4.605	4.536
**7cb**	4.606	4.590
**7cc**	4.615	4.578
**7da**	3.747	3.759
**7db**	3.747	3.757
**7dc**	3.751	3.756

pK_a calculations

pK_a calculations were performed using the method described by Lian et al.[17] Protonation of each amine was calculated, both from the neutral forms to the singly protonated forms, and from the singly protonated forms to the doubly-protonated forms. For every compound the single protonation with the higher pK_a was calculated to be amine of the sp^3 cage, so only pK_as corresponding to initial protonation of the amine in the sp^3 cage and subsequent protonation of the amine in the quinoline are listed in Table S4. All of the pK_a values for the first protonation are calculated to lie between 9.41 and 11.56, and all the pK_a values for the second protonation lie between 3.94 and 6.82. Slight trends are evident within these ranges for the different compounds (e.g. the addition of MeO groups in **7bx** and **7cx** results in higher $X \rightarrow X^+$ pK_a values than calculated for **7ax** and **7dx**, and **7xb** are calculated to have lower $X^+ \rightarrow X^{2+}$ pK_a values than **7xa** or **7xc**). It should be noted that the mean unsigned error of this method of 0.35 pK_a units for aliphatic amines limits the conclusions that can be drawn from these values.[17]

Table S4: Calculated pK_a values of the species using the method of Lian et al.[17]

Compound	pK_a ($X \rightarrow X^+$)	pK_a ($X^+ \rightarrow X^{2+}$)
**7aa**	11.63	4.81
**7ab**	10.82	3.94
**7ac**	10.67	5.73
**7ba**	10.78	5.80
**7bb**	9.41	5.63
**7bc**	10.39	6.82
**7ca**	11.56	6.14
**7cb**	11.23	6.47
**7cc**	11.71	6.45
**7da**	10.38	4.90
**7db**	9.55	4.87
**7dc**	10.76	5.31

Electronic excitation calculations

Excited state optimisations were performed using Pysisyphus[18] with Orca v5.0.4[19] at the ωB97X-D/6-31G(d) level, consistent with previous studies on the closely related molecule, quinine.[11] During optimisations the CPCM solvent model was used to describe the acetonitrile solvent.[20]

Subsequent single point energy calculations were carried out with Gaussian 16[15] at the ωB97X-D/6-311+G(d,p) level using non-equilibrium linear response IEFPCM solvation in acetonitrile.

Aside from localised carbonyl excited states, emission from which there is no experimental evidence for, the lowest-lying excited states were found to be π-π* quinoline-centred states. Additionally, charge-transfer states were identified for all compounds **7dx**. The characters of these excited states are shown in Figures S15 and S16.

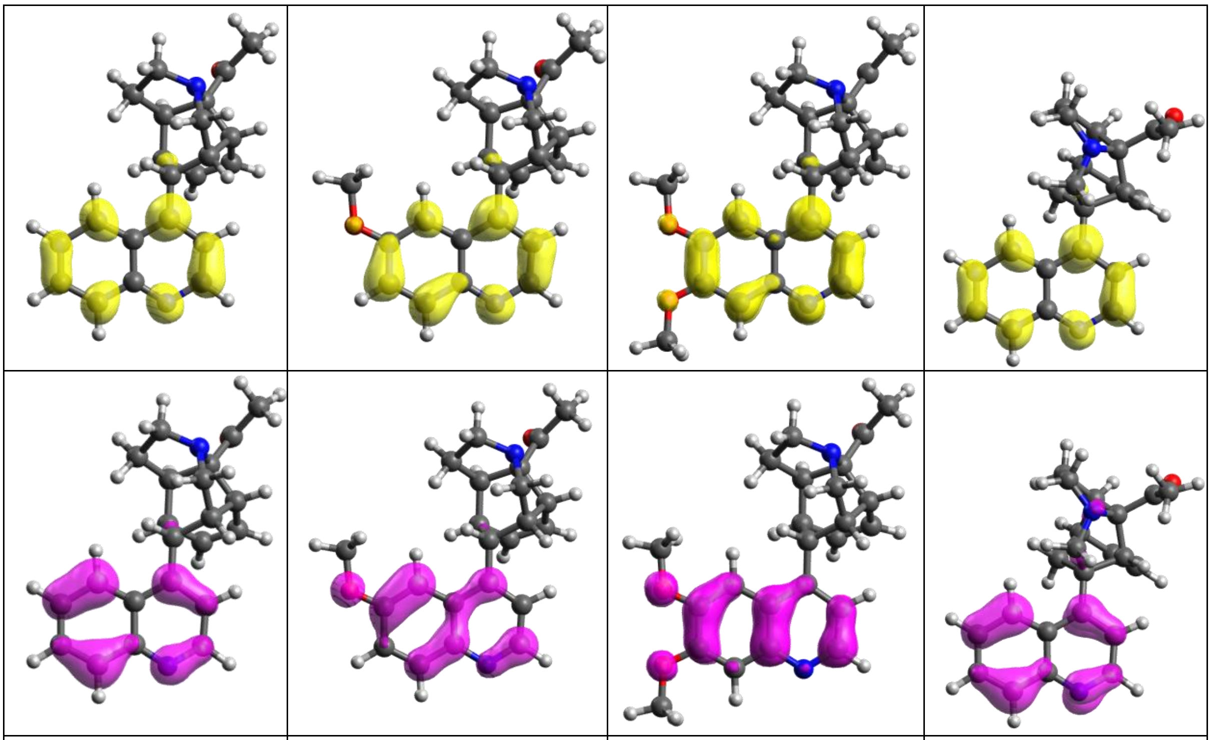

**7aa**	**7ba**	**7ca**	**7da**

Figure S15: Surfaces showing the holes (magenta) and electrons (yellow) calculated using Multiwfn v3.8[21] corresponding to the calculated π-π* locally excited states.

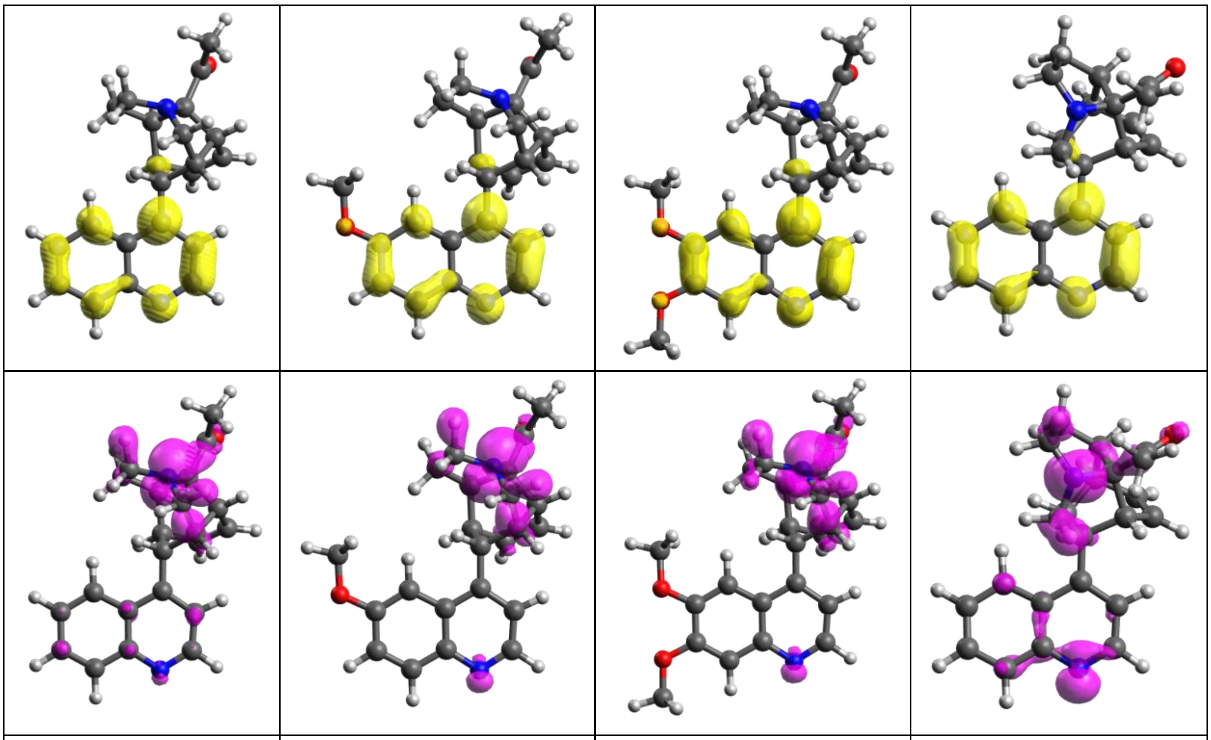

**7aa**	**7ba**	**7ca**	**7da**

Figure S16: Surfaces showing the holes (magenta) and electrons (yellow) calculated using Multiwfn v3.8[21] corresponding to the calculated charge-transfer excited states.

Energies of these excited states are given in Table S5, and the relative energies shown plotted in Figure S17.

Table S5: energies of the optimised excited states relative to the optimised ground state energies.

	π-π* / eV	CT / eV
**7aa**	4.24	5.04
**7ba**	4.03	5.19
**7ca**	4.17	5.25
**7da**	4.27	4.84

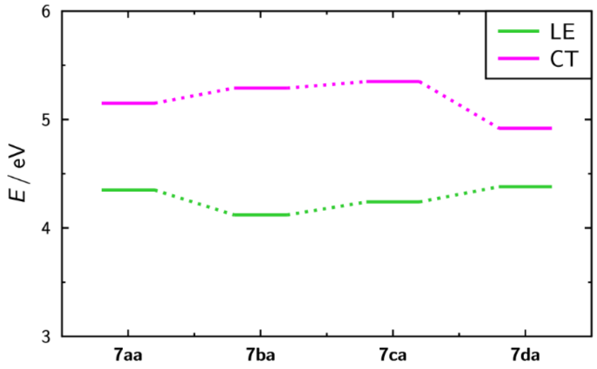

Figure S17: energies of the optimised excited states relative to the optimised ground state energies

Although the calculations suggest the CT states all lie at higher energy than the LE states, the overestimation of CT states is consistent with previous reports.[11] State-specific solvation methods were tested but resulted in the CT states all lying lower in energy than the respective LE states, and this underestimate is again consistent with previous work on quinine.[11]

8. Protonation Studies on Compound 7ab

A solution of compound **7ab** (10 mg, 0.028 mmol) in D_2O and d_4-MeOH (1.0 mL, 1:1, 0.028 M in **7ab**) was analysed by ^{1}H and ^{13}C NMR spectroscopy (Figures S18 and S19). Concentrated H_2SO_4 (6.0 µL, 0.11 mmol, 0.11 M, 3.9 equiv.) was added and the sample reanalysed by ^{1}H and ^{13}C NMR spectroscopy (Figures S20 and S21). Comparison of these spectra shows significant changes in chemical shift of the environments neighbouring both amine moieties following addition of the acid, which are consistent with double protonation.

Figure S18: ^{1}H NMR (400 MHz) spectrum of unprotonated **7ab** in D_2O/d_4-MeOH

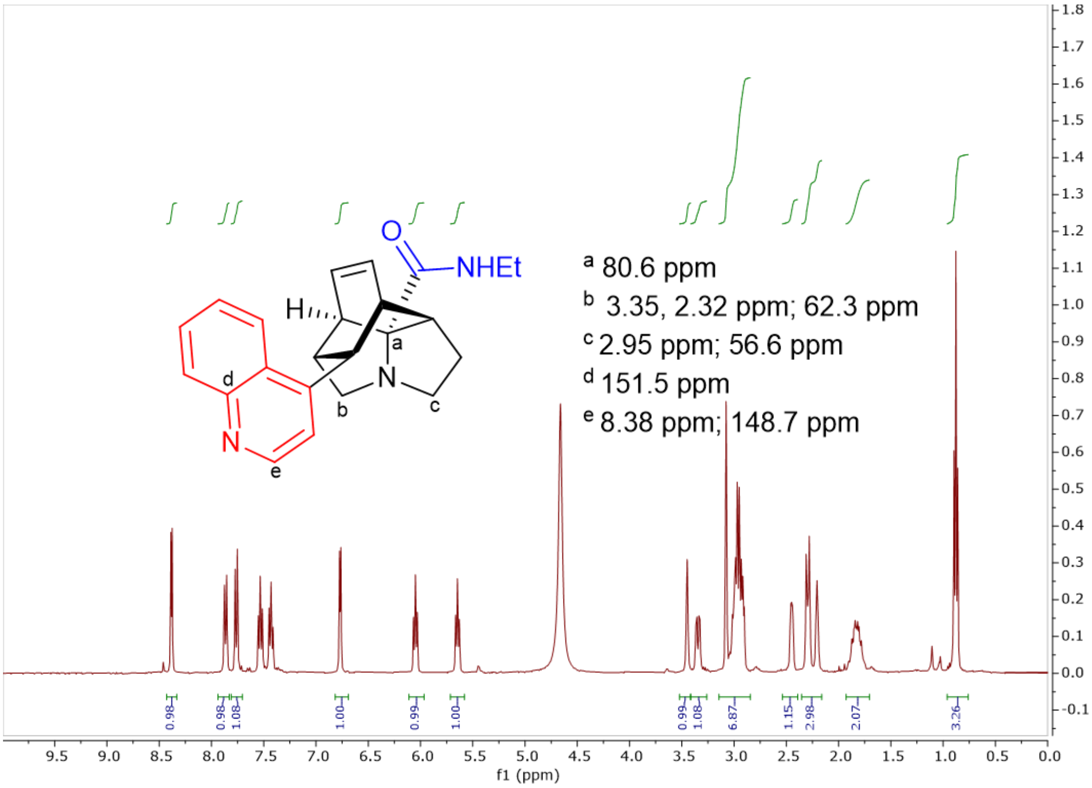

Figure S19: ^{13}C NMR (101 MHz) spectrum of unprotonated **7ab** in D_2O/d_4-MeOH

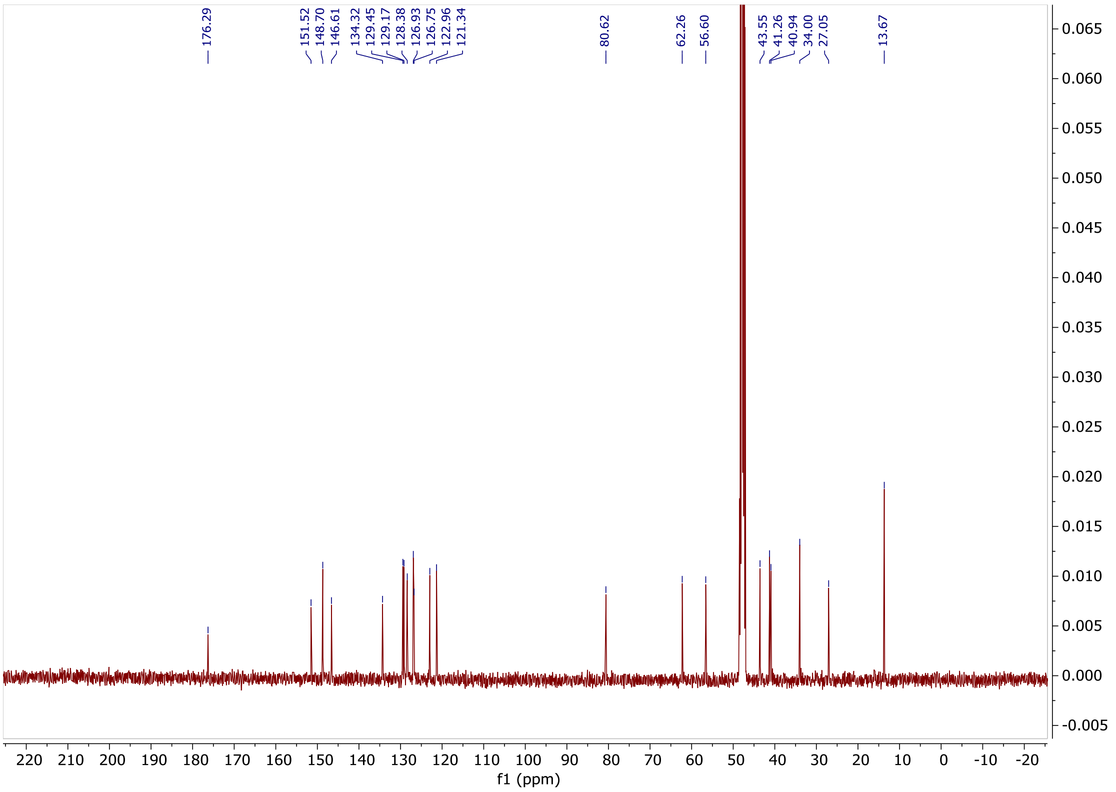

Figure S20: ^{1}H NMR (400 MHz) spectrum of **7ab** in D_2O/d_4-MeOH following addition of H_2SO_4

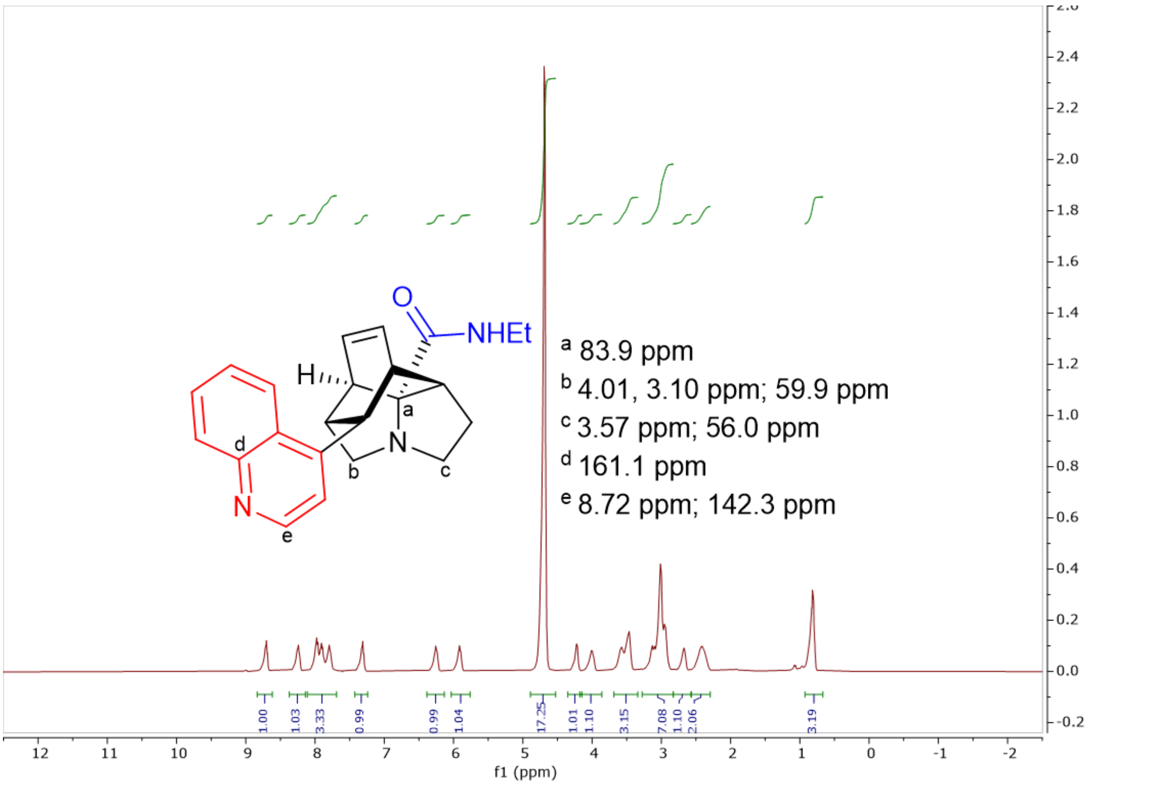

Figure S21: ^{13}C NMR (101 MHz) spectrum of **7ab** in D_2O/d_4-MeOH following addition of H_2SO_4

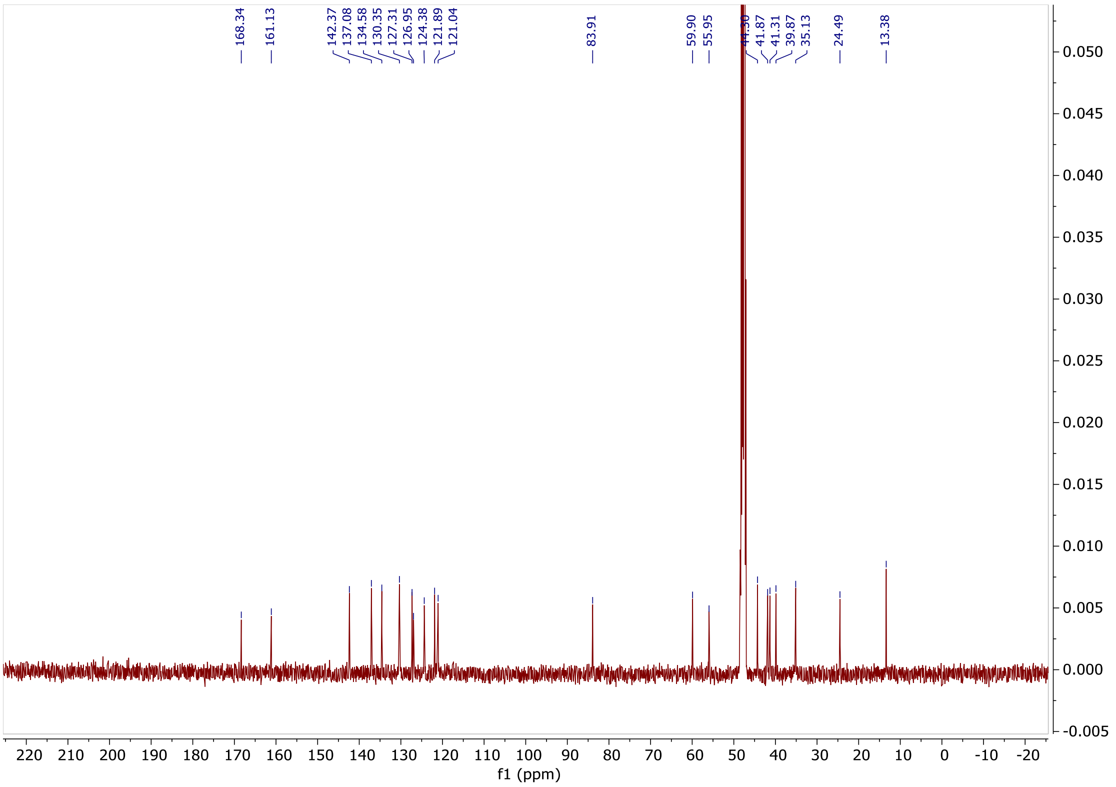

9. References

(1) De, K.; Legros, J.; Crousse, B.; Bonnet-Delpon, D. Solvent-Promoted and -Controlled Aza-Michael Reaction with Aromatic Amines. *J. Org. Chem.* **2009**, *74*, 6260-6265.

(2) Friestad, G. K.; Ji, A.; Baltrusaitis, J.; Korapala, C. S.; Qin, J. Scope of Stereoselective Mn-Mediated Radical Addition to Chiral Hydrazones and Application in a Formal Synthesis of Quinine. *J. Org. Chem.* **2012**, *77*, 3159-3180.

(3) Kutsumura, N.; Matsubara, Y.; Niwa, K.; Ito, A.; Saito, T. One-Pot Method for Regioselective Bromin-ation and Sequential Carbon–Carbon Bond-Forming Reactions of Allylic Alcohol Derivatives. *Eur. J. Org. Chem.* **2013**, 3337-3346.

(4) Maskill, K. G.; Knowles, J. P.; Elliott, L. D.; Alder, R. W.; Booker-Milburn, K. I. Complexity from Simplicity: Tricyclic Aziridines from the Rearrangement of Pyrroles by Batch and Flow Photochemistry. *Angew. Chem. Int. Ed.* **2013**, *52*, 1499-1502.

(5) Knowles, J. P.; Steeds, H. G.; Schwarz, M.; Latter, F.; Booker-Milburn, K. I. Pd-Catalyzed Cascade Reactions of Aziridines: One-Step Access to Complex Tetracyclic Amines. *Org. Lett.* **2021**, *23*, 4986-4990.

(6) Garza, V. J.; Krische, M. J. Hydroxymethylation beyond Carbonylation: Enantioselective Iridium-Catalyzed Reductive Coupling of Formaldehyde with Allylic Acetates via Enantiotopic π-Facial Discrimination. *J. Am. Chem. Soc.* **2016**, *138*, 3655–3658.

(7) Levitz, M.; Bogert, M. T. The Search for Superior Drugs for Tropical Diseases. I. Derivatives of Quininaldehyde and 6,7-Dimethoxycinchoninaldehyde. *J. Org. Chem.* **1945**, *10*, 341-346.

(8) Grimaux, E. *Bull. Chim. Soc. Fr.* **1882**, *38*, 127.

(9) Cresswell, A. J.; Lloyd-Jones, G. C. Room-Temperature Gold-Catalysed Arylation of Heteroarenes: Complementarity to Palladium Catalysis. *Chem. Eur. J.* **2016**, *22*, 12641-12645.

(10) Steeds, H. G.; Knowles, J. P.; Yu, W. L.; Richardson, J.; Cooper, K. G.; Booker-Milburn, K. I. Rapid Access to Azabicyclo[3.3.1]nonanes by a Tandem Diverted Tsuji–Trost Process. *Chem. Eur. J.* **2020**, *26*, 14330-14334.

(11) Turley, A. T.; Danos, A.; Prlj, A.; Monkman, A. P.; Curchod, B. F. E.; McGonigal, R. R.; Etherington, M. K. Modulation of Charge Transfer by N-Alkylation to Control Photoluminescence Energy and Quantum Yield. *Chem. Sci.* **2020**, *11*, 6990-6995.

(12) Grimme, S. Exploration of Chemical Compound, Conformer, and Reaction Space with Meta-Dynamics Simulations Based on Tight-Binding Quantum Chemical Calculations. *J. Chem. Theory Comput.* **2019**, *15*, 2847–2862.

(13) Pracht, P.; Bohle, F.; Grimme, S. Automated Exploration of the Low-Energy Chemical Space with Fast Quantum Chemical Methods. *Phys. Chem. Chem. Phys.* **2020**, *22*, 7169–7192.

(14) Ehlert, S.; Stahn, M.; Spicher, S.; Grimme, S. Robust and Efficient Implicit Solvation Model for Fast Semiempirical Methods. *J. Chem. Theory Comput.* **2021**, *17*, 4250–4261.

(15) Frisch, M. J.; Trucks, G. W.; Schlegel, H. B.; Scuseria, G. E.; Robb, M. A.; Cheeseman, J. R.; Scalmani, G.; Barone, V.; Mennucci, B.; Petersson, G. A.; Nakatsuji, H.; Caricato, M.; Li, X.; Hratchian, H. P.; Izmaylov, A. F.; Bloino, J.; Zheng, G.; Sonnenberg, J. L.; Hada, M.; Ehara, M.; Toyota, K.; Fukuda, R.; Hasegawa, J.; Ishida, M.; Nakajima, T.; Honda, Y.; Kitao, O.; Nakai, H.; Vreven, T.; Montgomery, J. J. A.; Peralta, J. E.; Ogliaro, F.; Bearpark, M.; Heyd, J. J.; Brothers, E.; Kudin, K. N.; Staroverov, V. N.; Kobayashi, R.; Normand, J.; Raghavachari, K.; Rendell, A.; Burant, J. C.; Iyengar, S. S.; Tomasi, J.; Cossi, M.; Rega, N.; Millam, J. M.; Klene, M.; Knox, J. E.; Cross, J. B.; Bakken, V.; Adamo, C.; Jaramillo, J.; Gomperts, R.; Stratmann, R. E.; Yazyev, O.; Austin, A. J.; Cammi, R.; Pomelli, C.; Ochterski, J.

W.; Martin, R. L.; Morokuma, K.; Zakrzewski, V. G.; Voth, G. A.; Salvador, P.; Dannenberg, J. J.; Dapprich, S.; Daniels, A. D.; Farkas, Ö.; Foresman, J. B.; Ortiz, J. V.; Cioslowski, J.; Fox, D. J. Gaussian 16, Revision C.01, 2019

(16) Chai, J.-D.; Head-Gordon, M. Systematic Optimization of Long-Range Corrected Hybrid Density Functionals. *J. Chem. Phys*. **2008**, *128*, 084106.

(17) Lian, P.; Johnston, R. C.; Parks, J. M.; Smith, J. C. Quantum Chemical Calculation of p*K*as of Environmentally Relevant Functional Groups: Carboxylic Acids, Amines, and Thiols in Aqueous Solution. *J. Phys. Chem. A* **2018**, *122*, 4366–4374.

(18) Steinmetzer, J.; Kupfer, S.; Gräfe, S. Pysisyphus: Exploring Potential Energy Surfaces in Ground and Excited States. *Int. J. Quantum Chem.* **2020**, *121*, e26390.

(19) (a) F. Neese, The Orca Program System. *Wiley Interdiscip. Rev. Comput. Mol. Sci.* **2012**, 2, 73−78. (b) F. Neese, Software Update: The Orca Program System – Version 5.0. *Wiley Interdiscip. Rev. Comput. Mol. Sci.* **2022,** 12, e1606.

(20) Barone, V.; Cossi, M. Quantum Calculation of Molecular Energies and Energy Gradients in Solution by a Conductor Solvent Model. *J. Phys. Chem. A* **1998**, *11*, 1995–2001.

(21) Lu, T.; Chen, F. Multiwfn: A Multifunctional Wavefunction Analyzer. *J. Comp. Chem*. **2012**, *33*, 580–592.

Monomethoxy quinoline acetate 8b

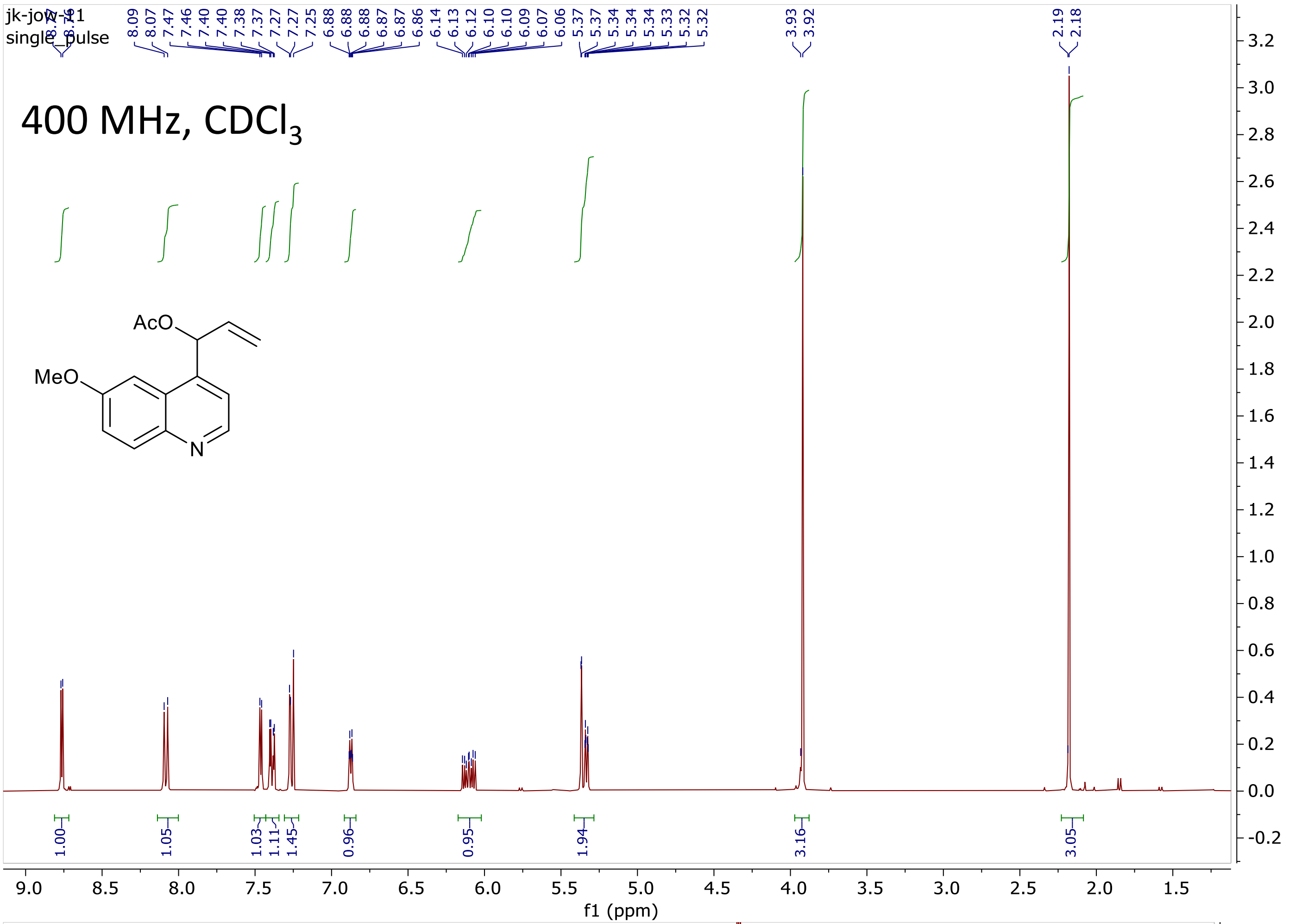

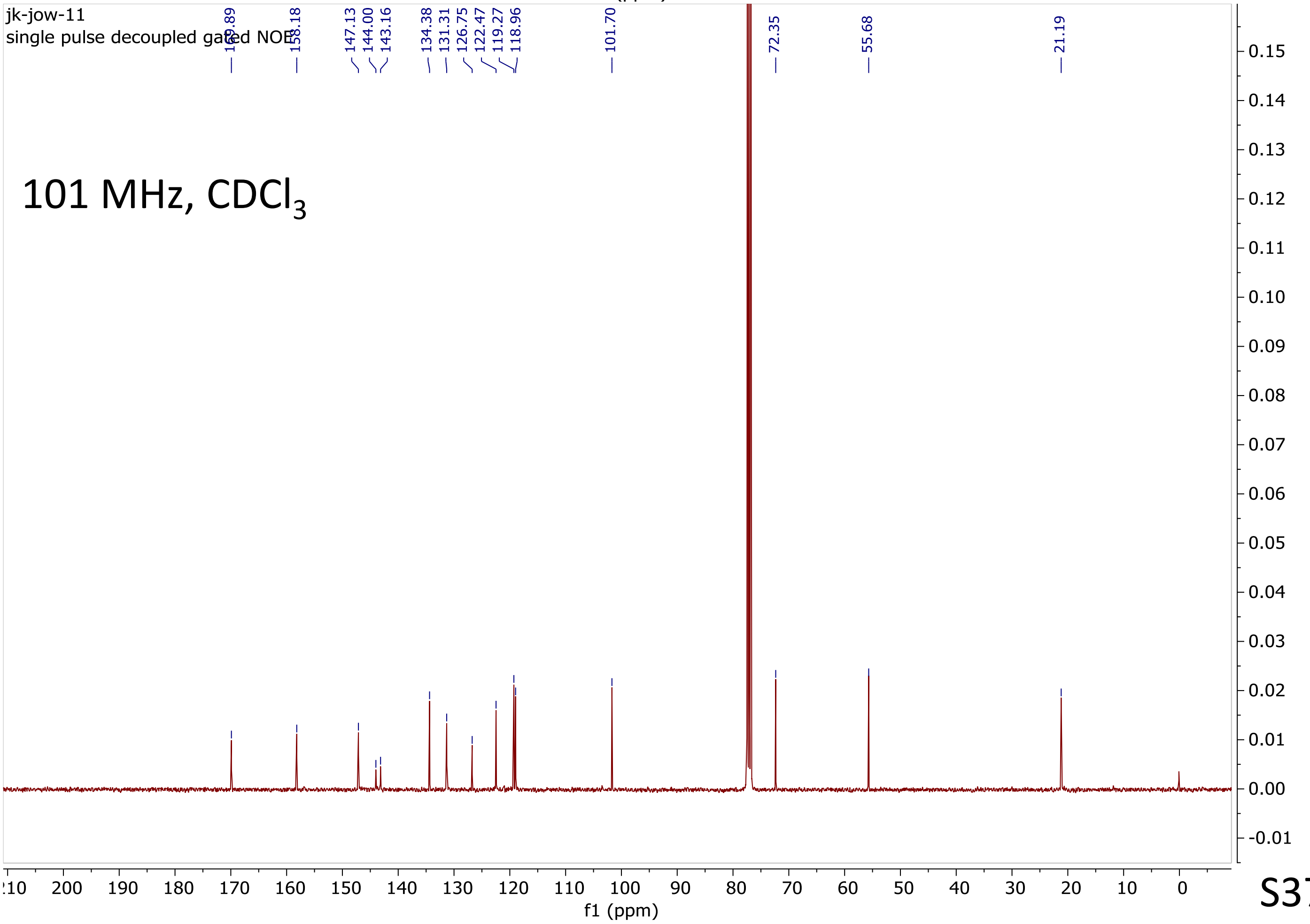

Dimethoxy quinoline acetate 8c

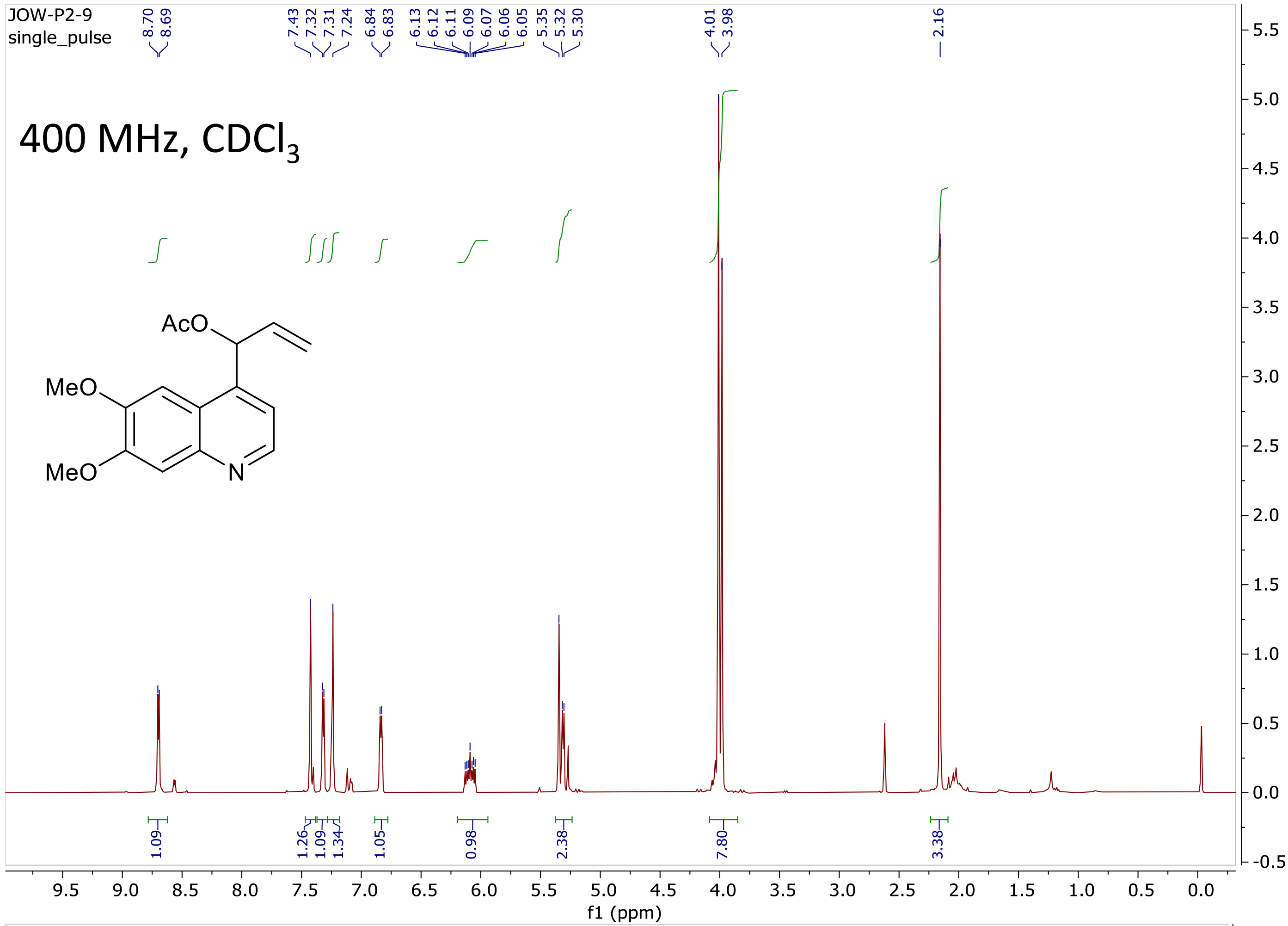

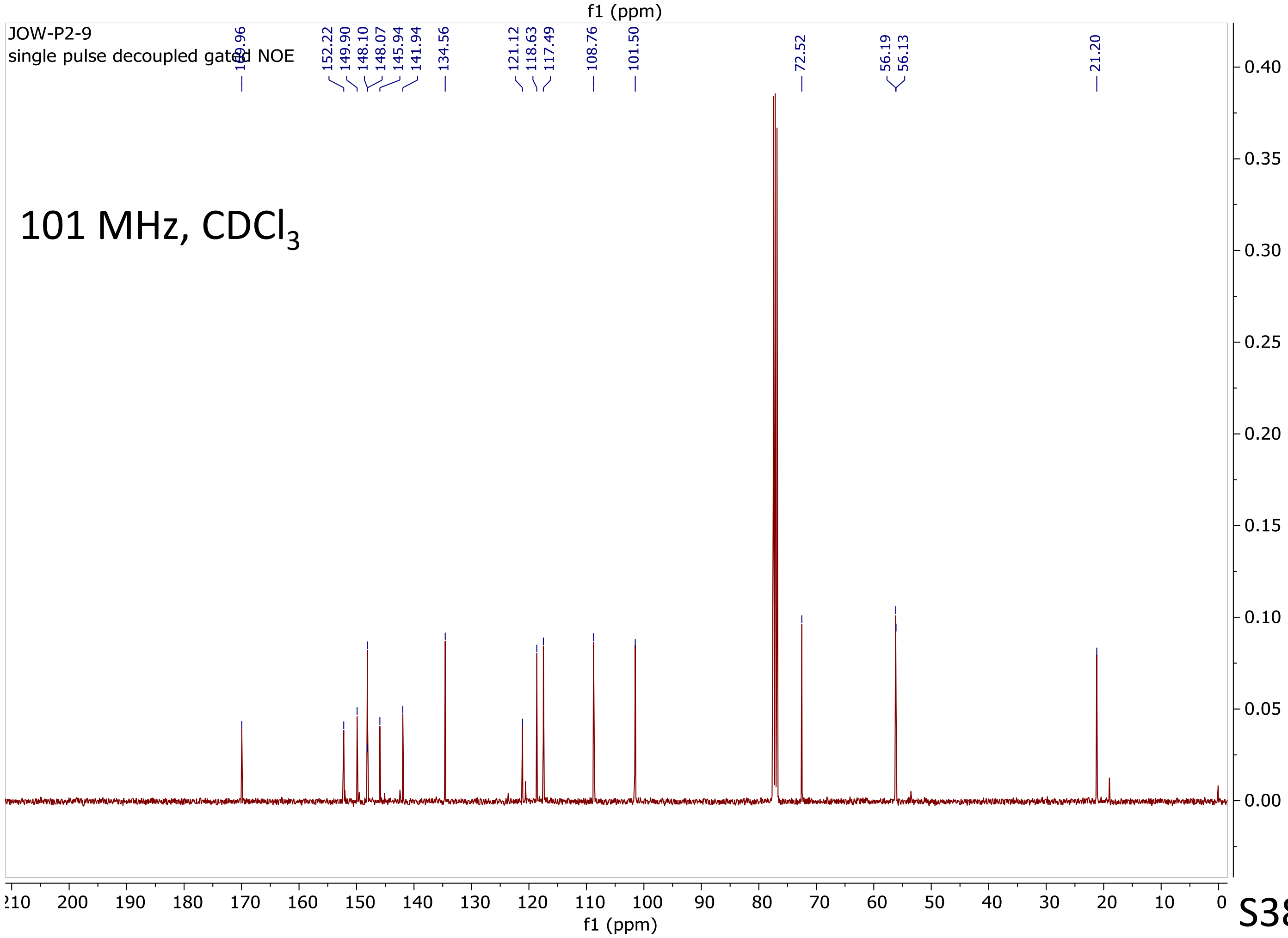

THP bromide S5b

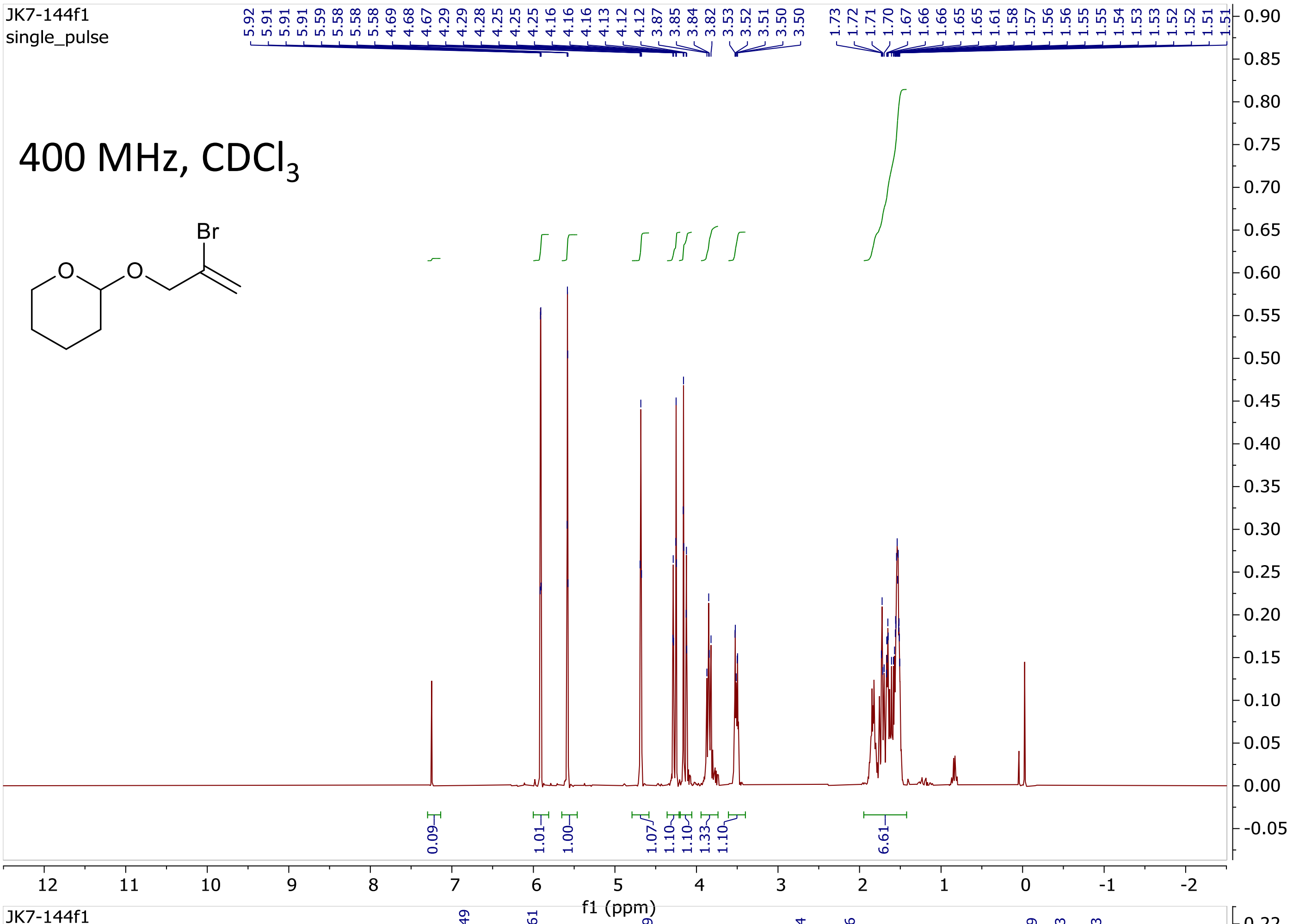

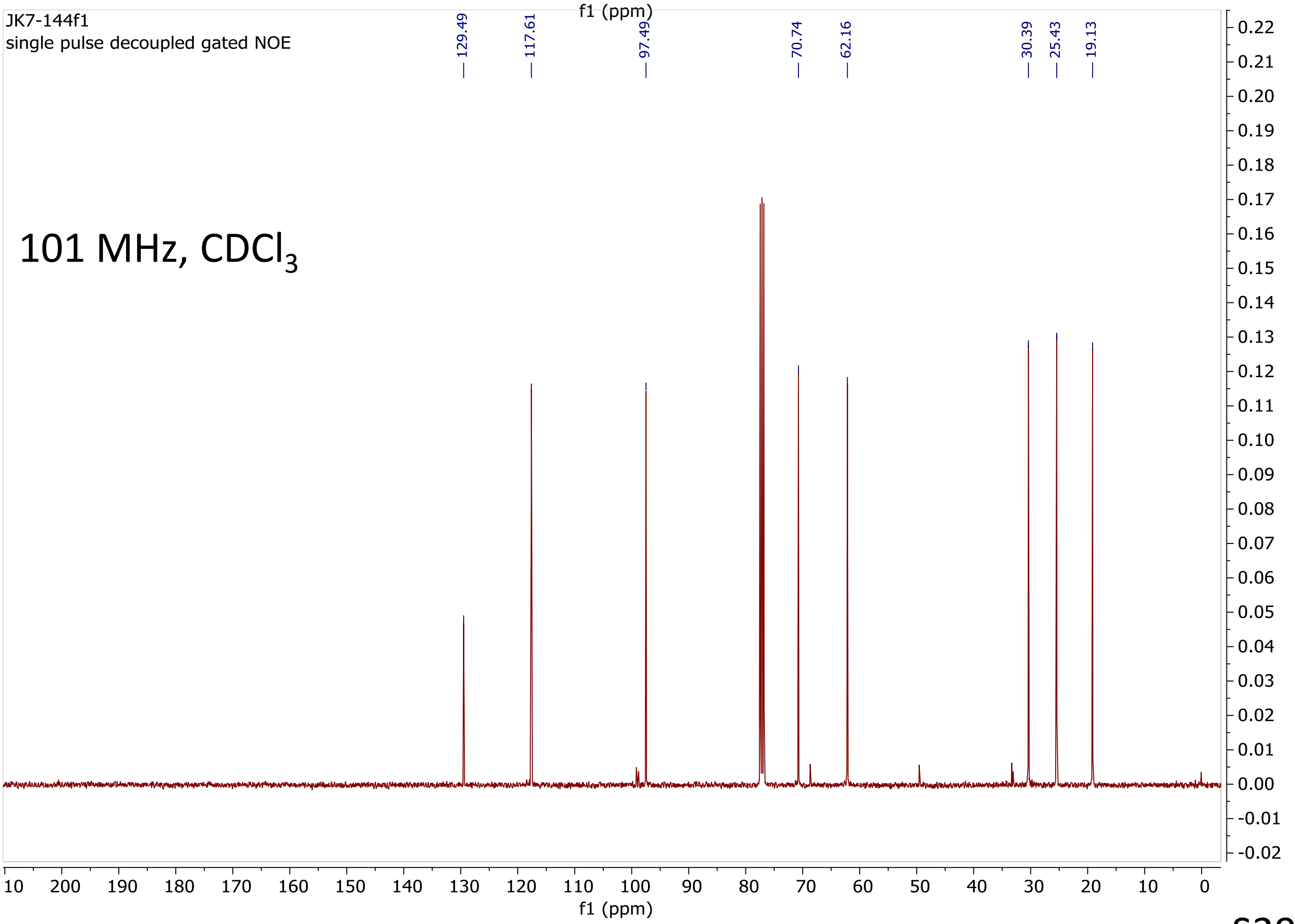

Quinoline THP S6b

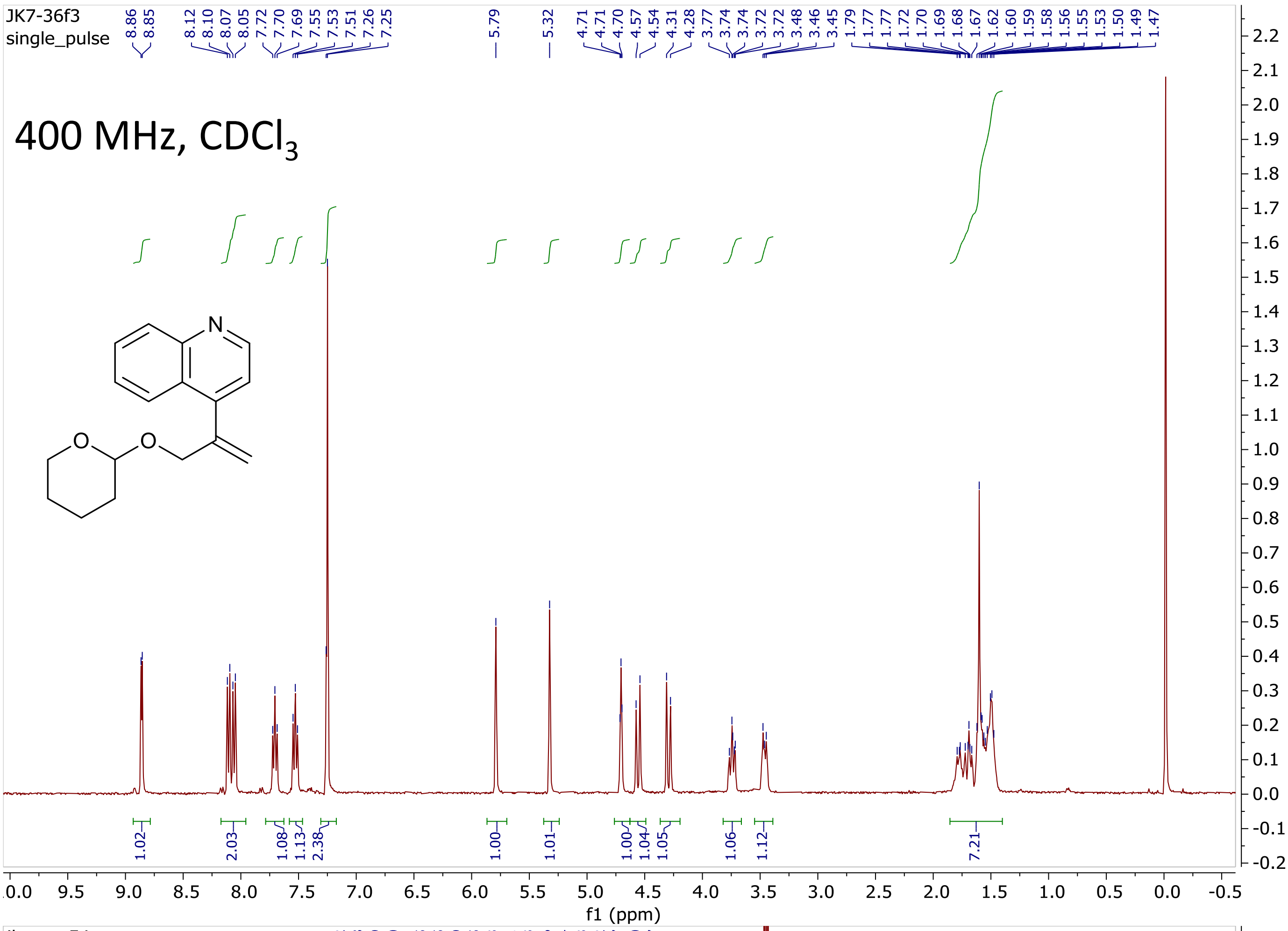

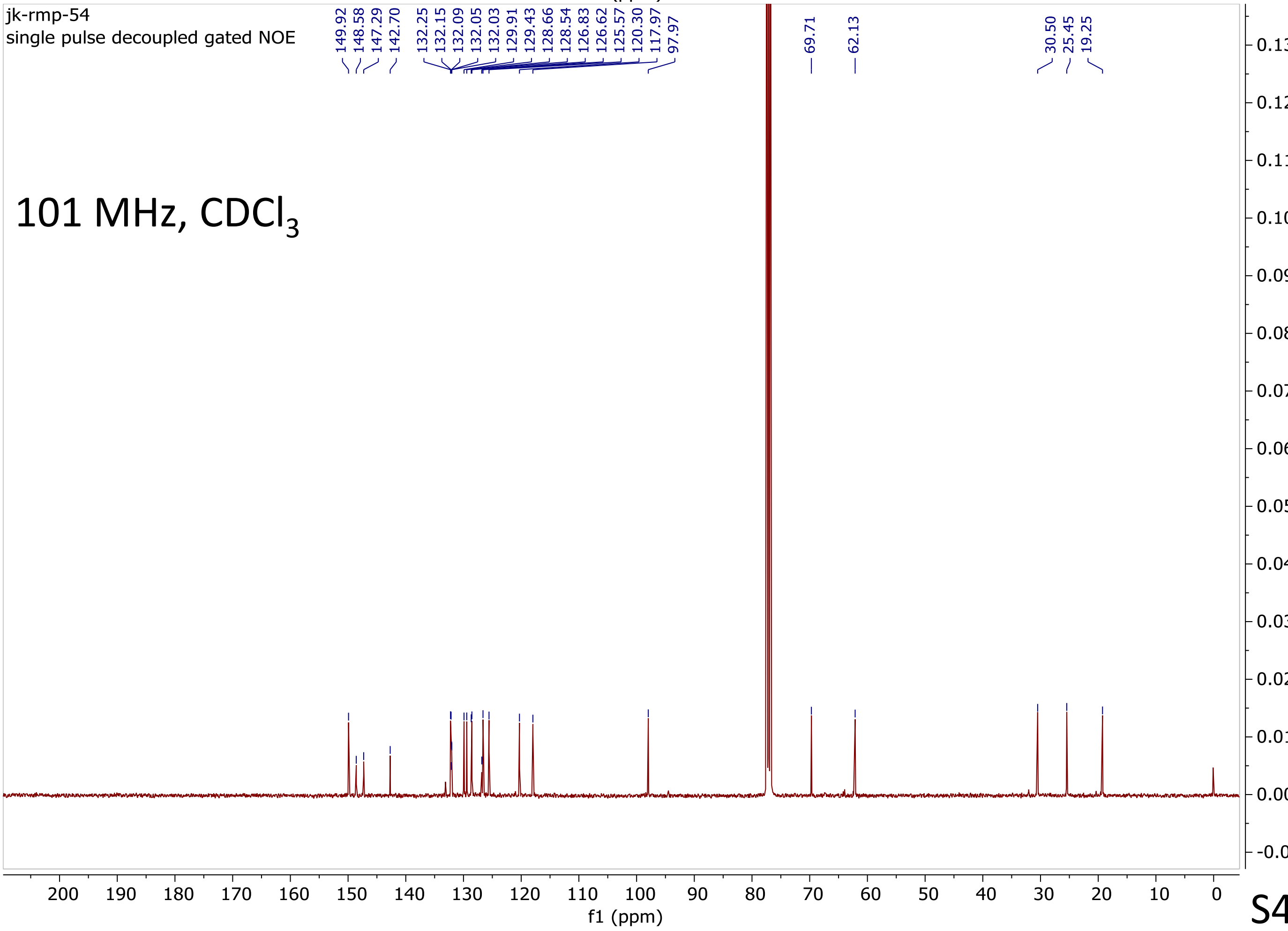

Quinoline alcohol S7

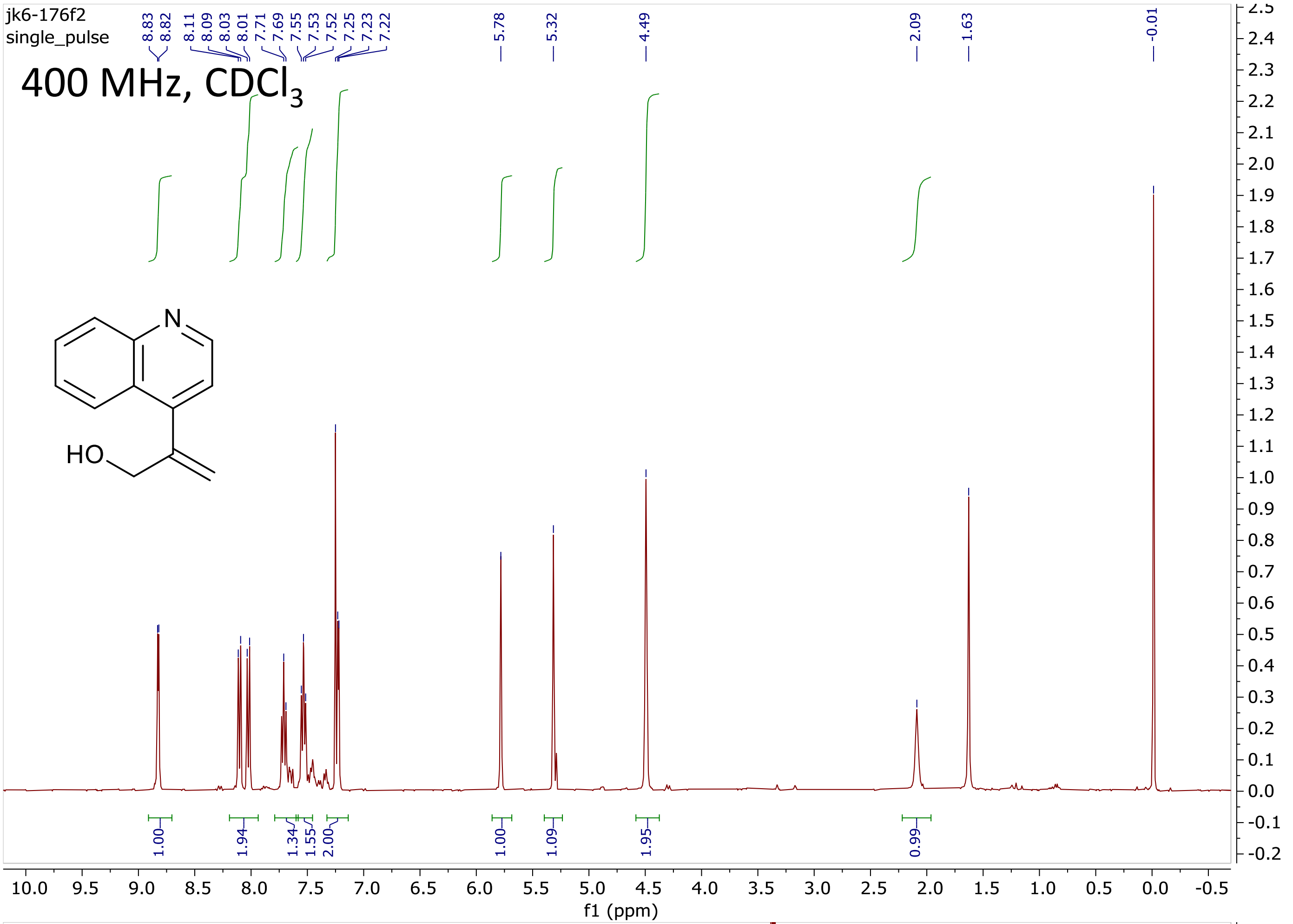

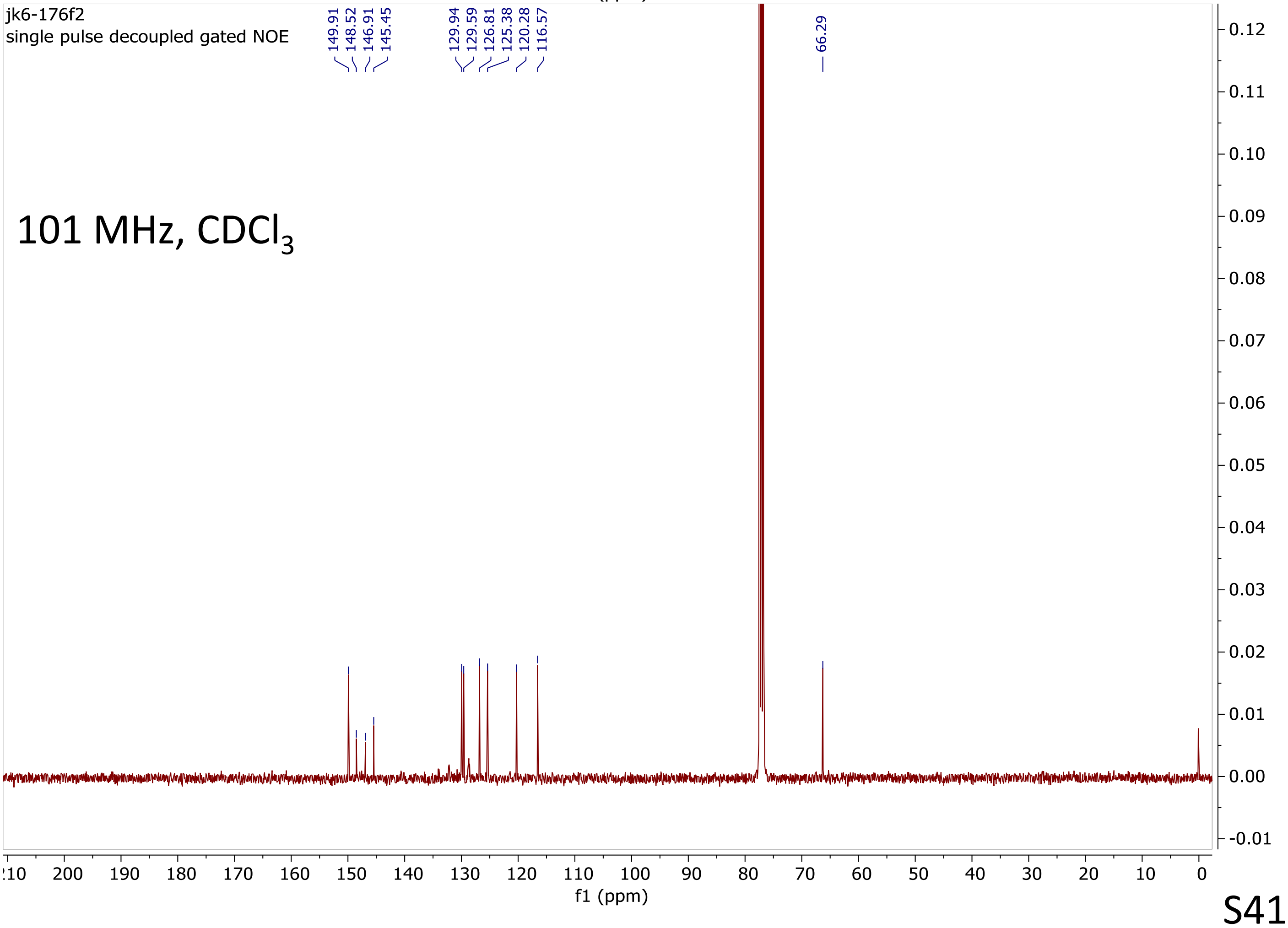

Quinoline acetate 9

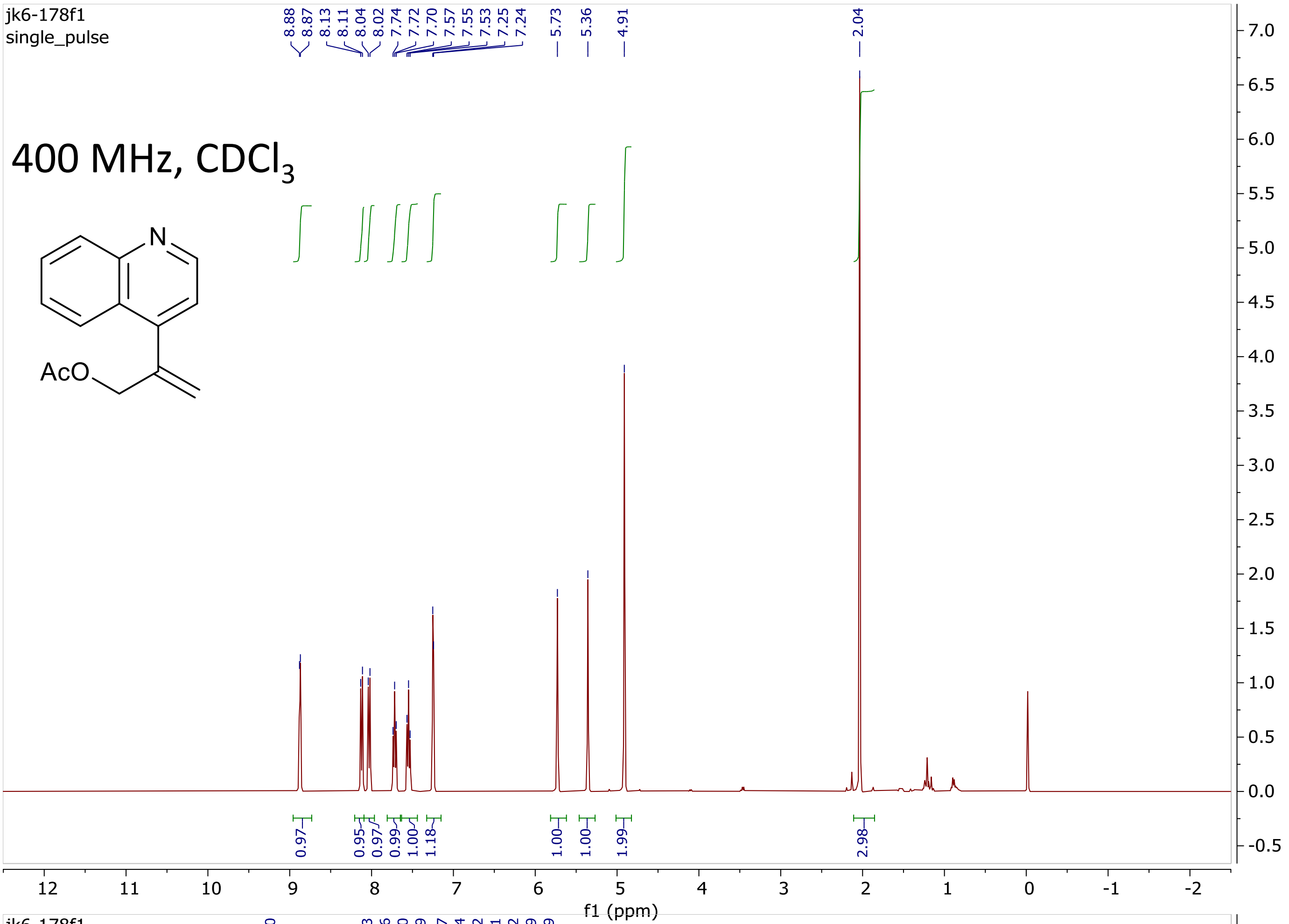

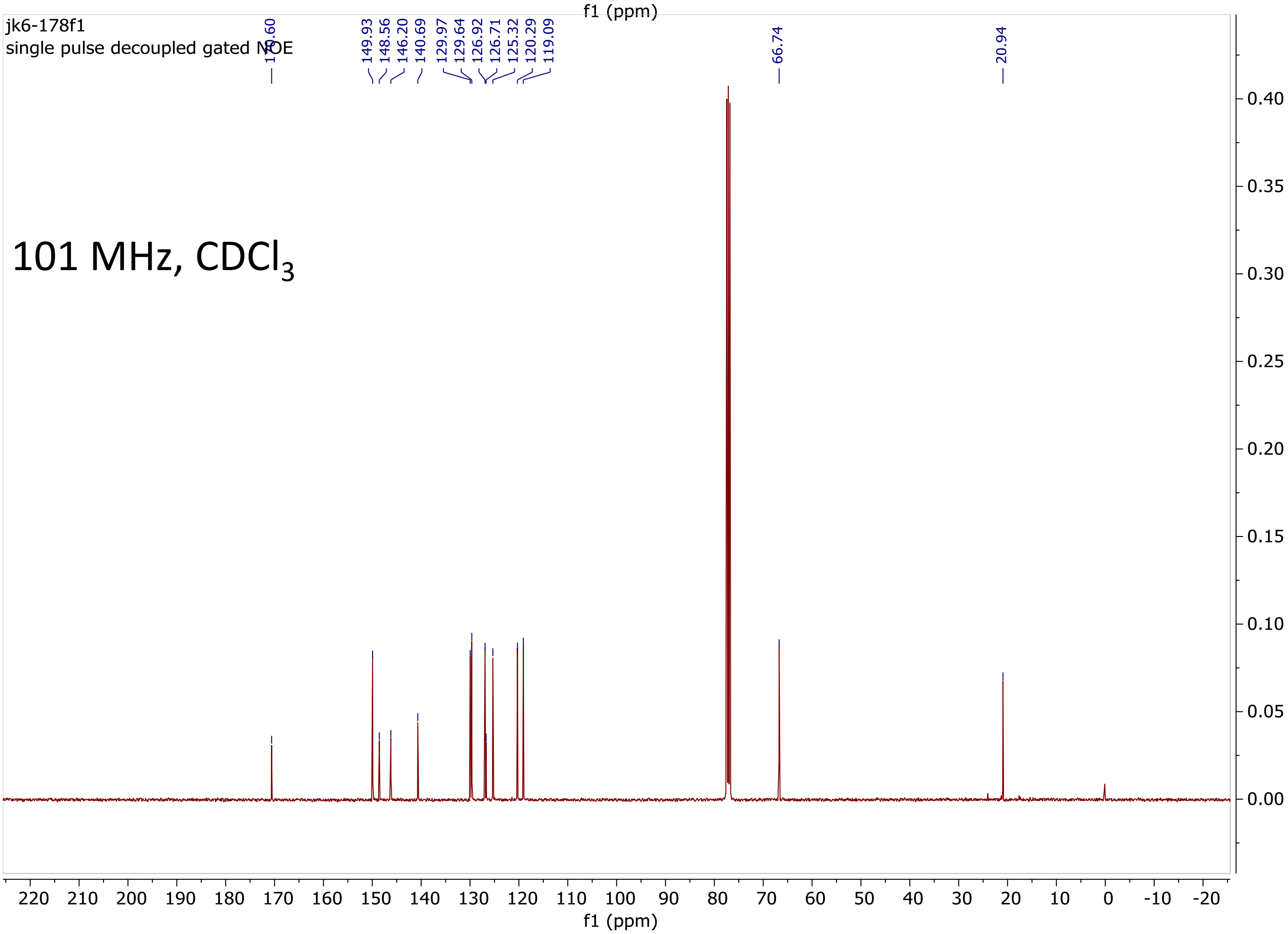

Cascade product 7aa

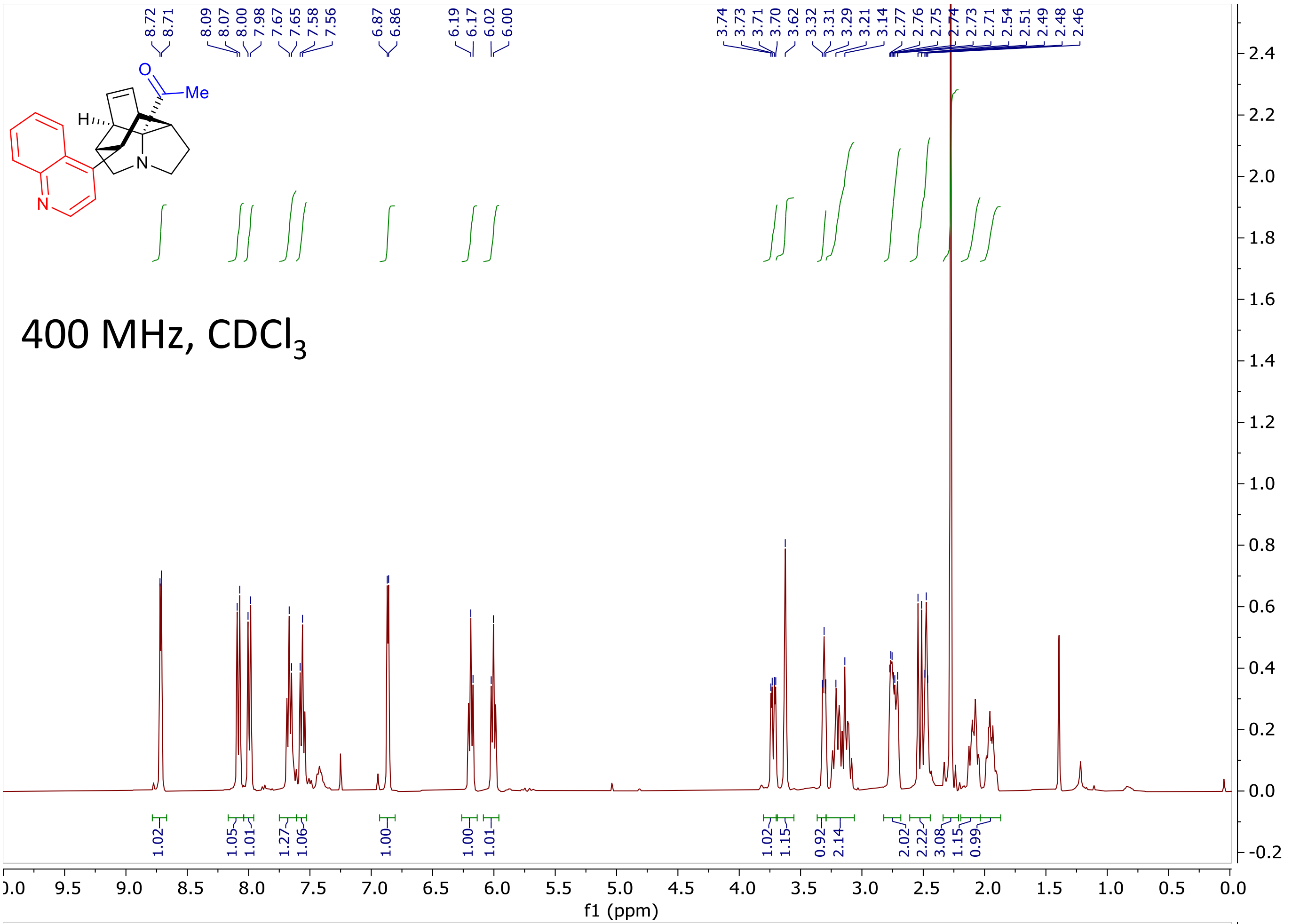

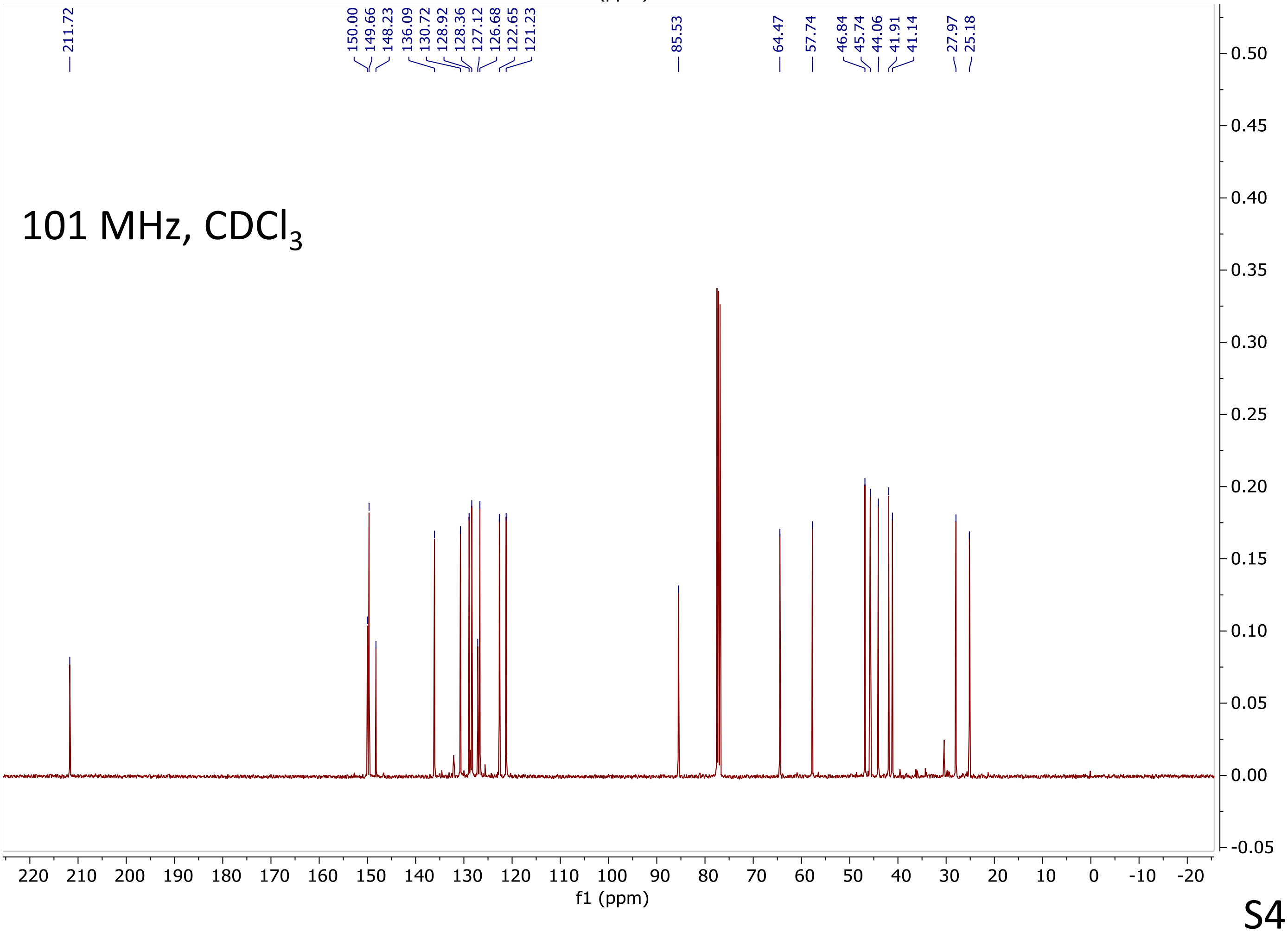

Cascade product 7ab

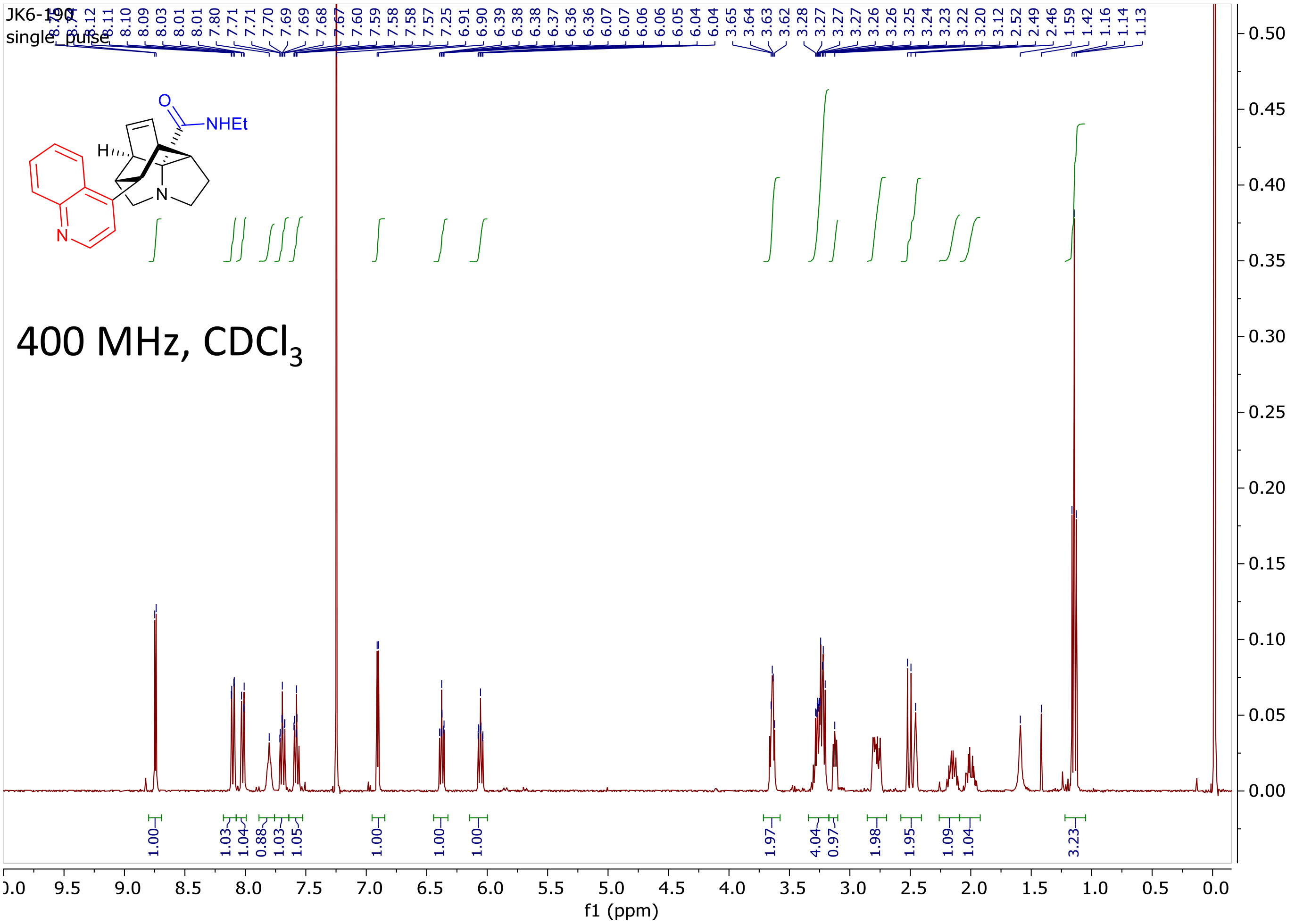

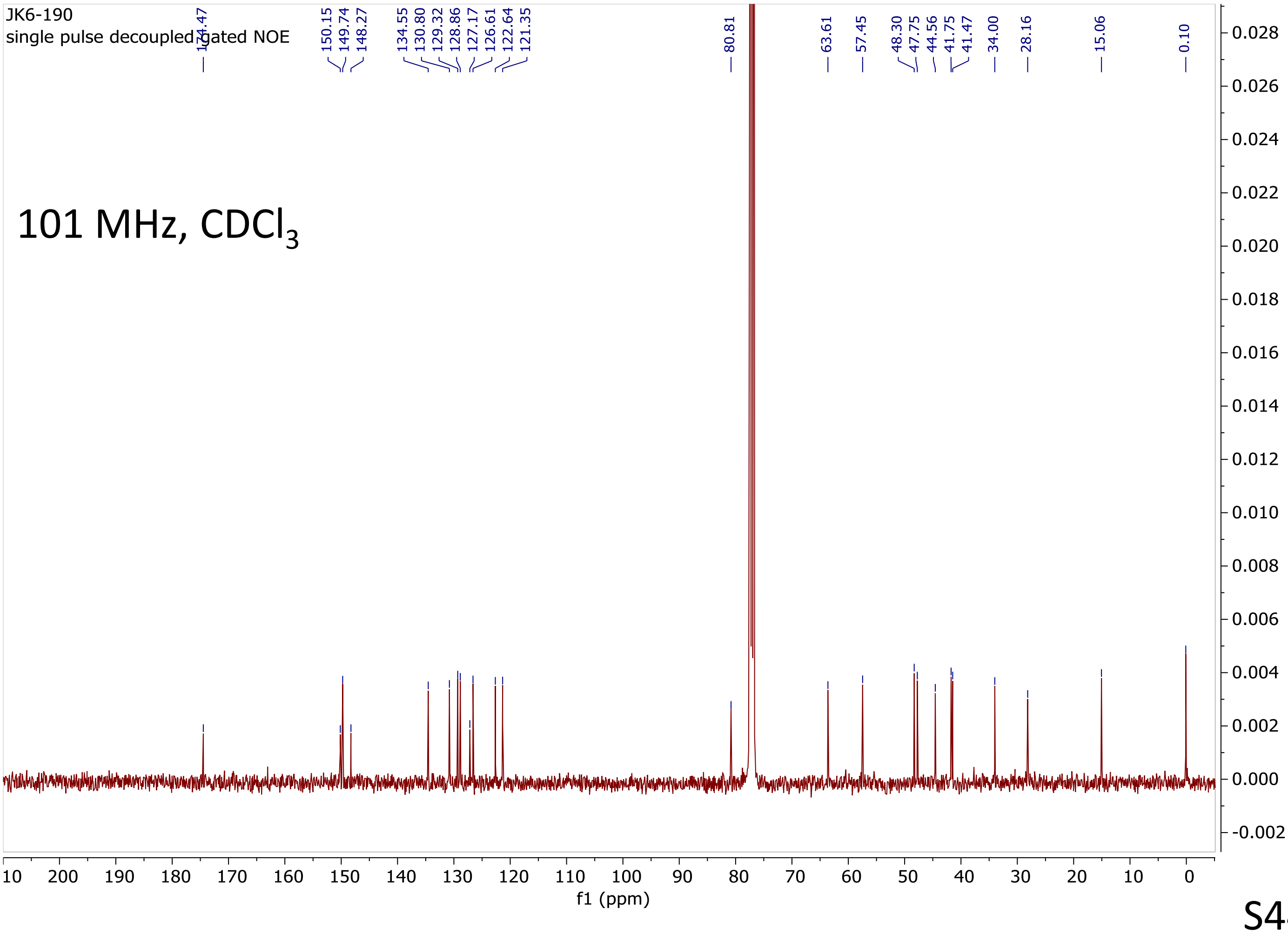

Cascade product 7ac

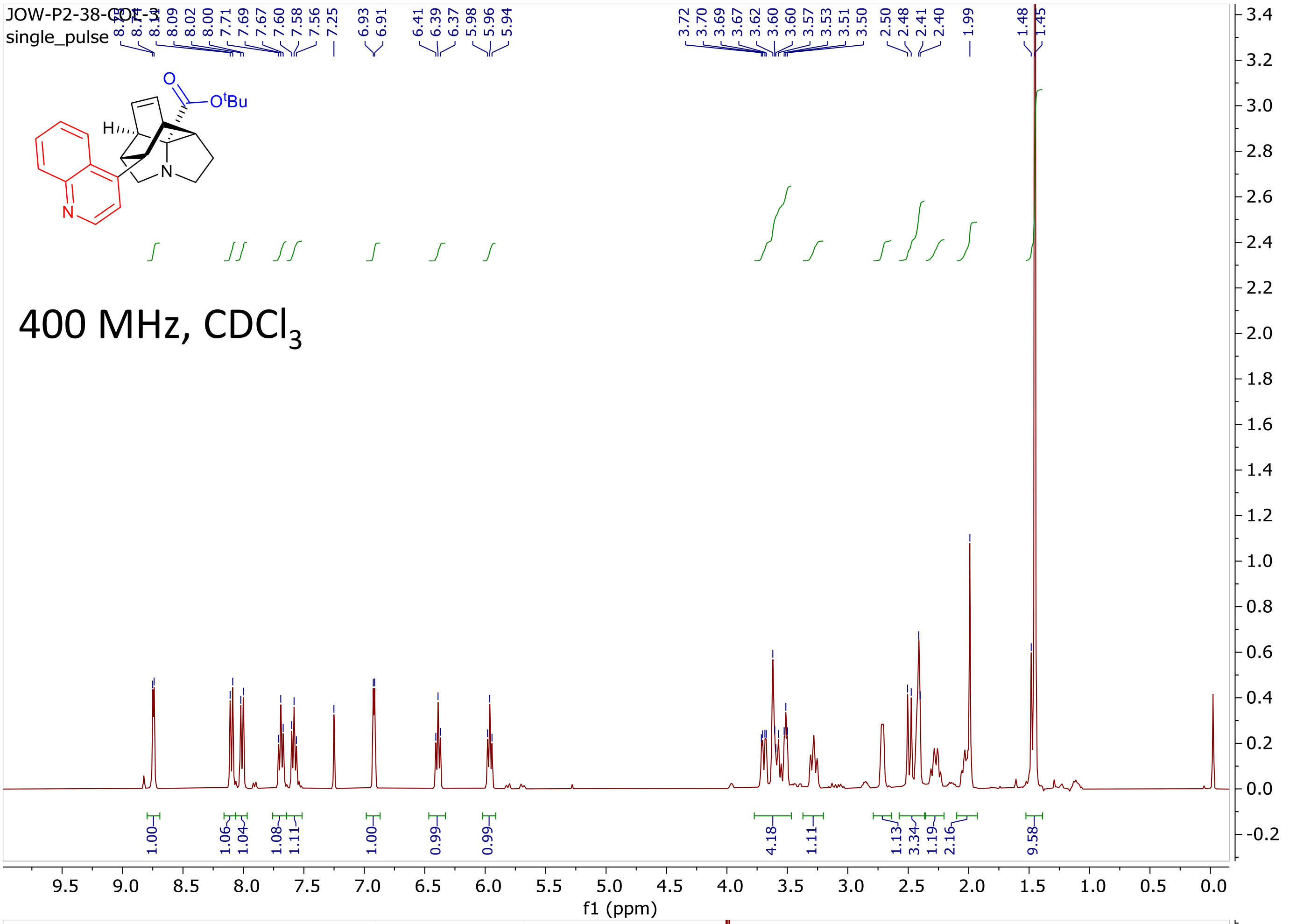

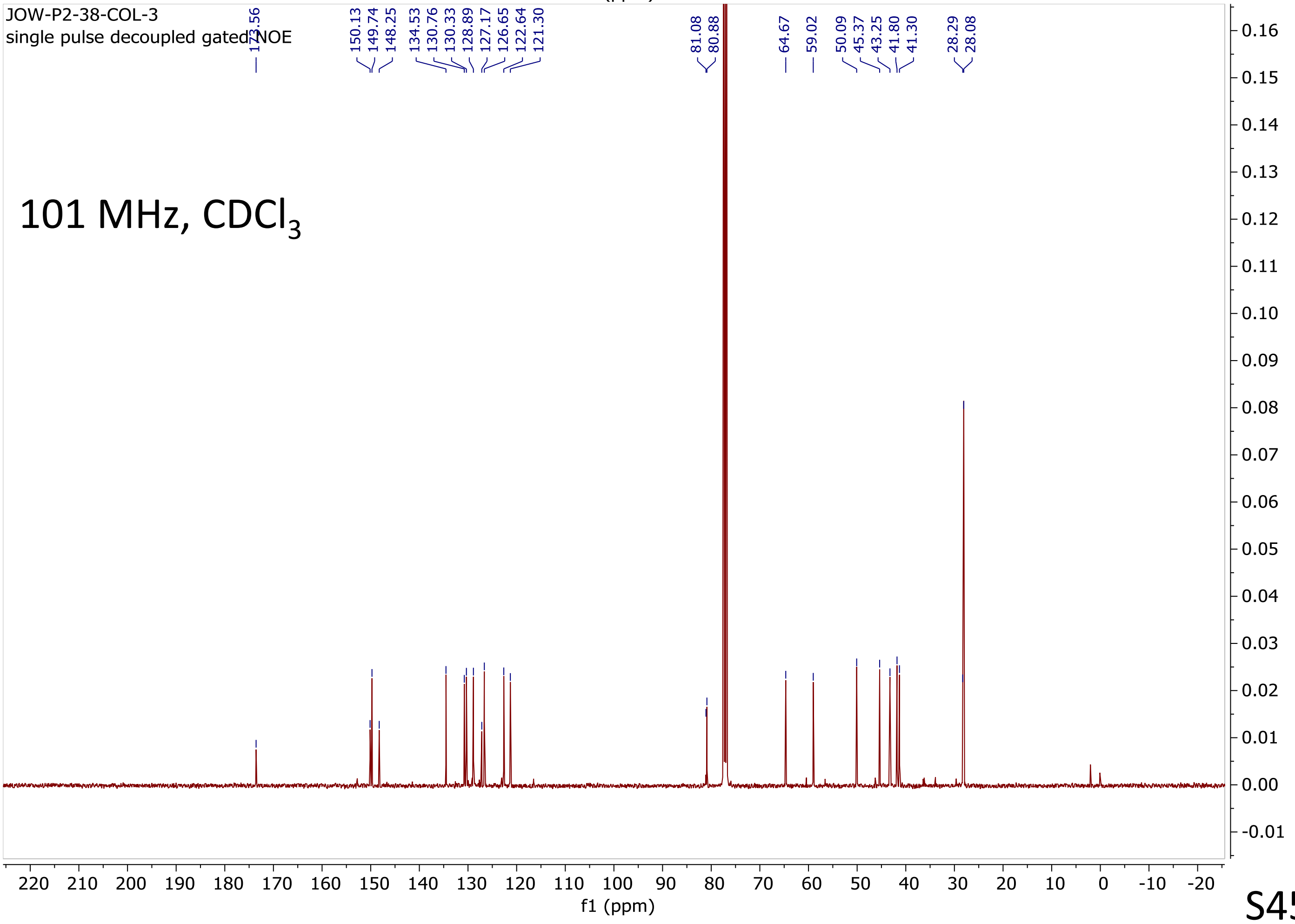

Cascade product 7ba

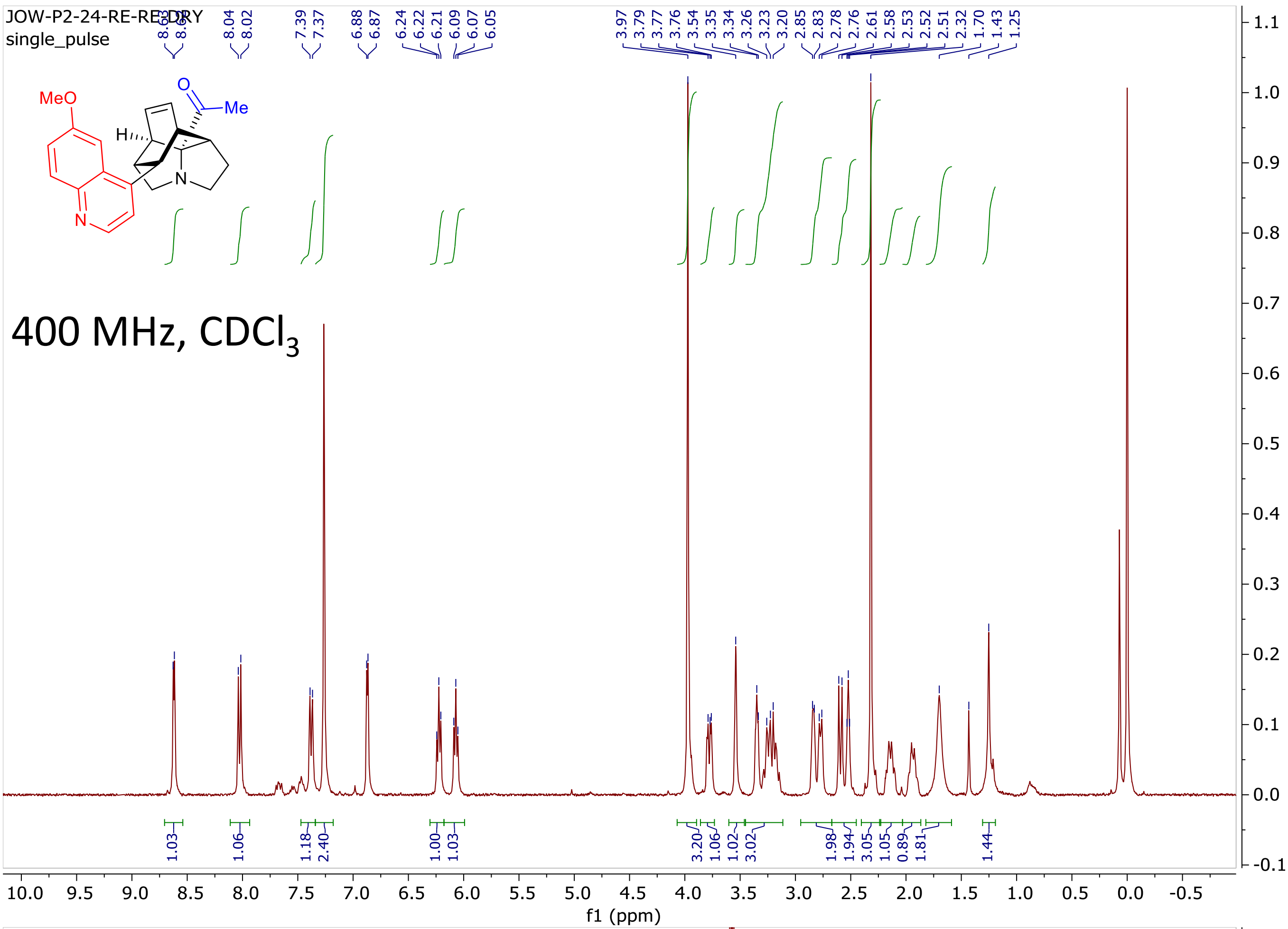

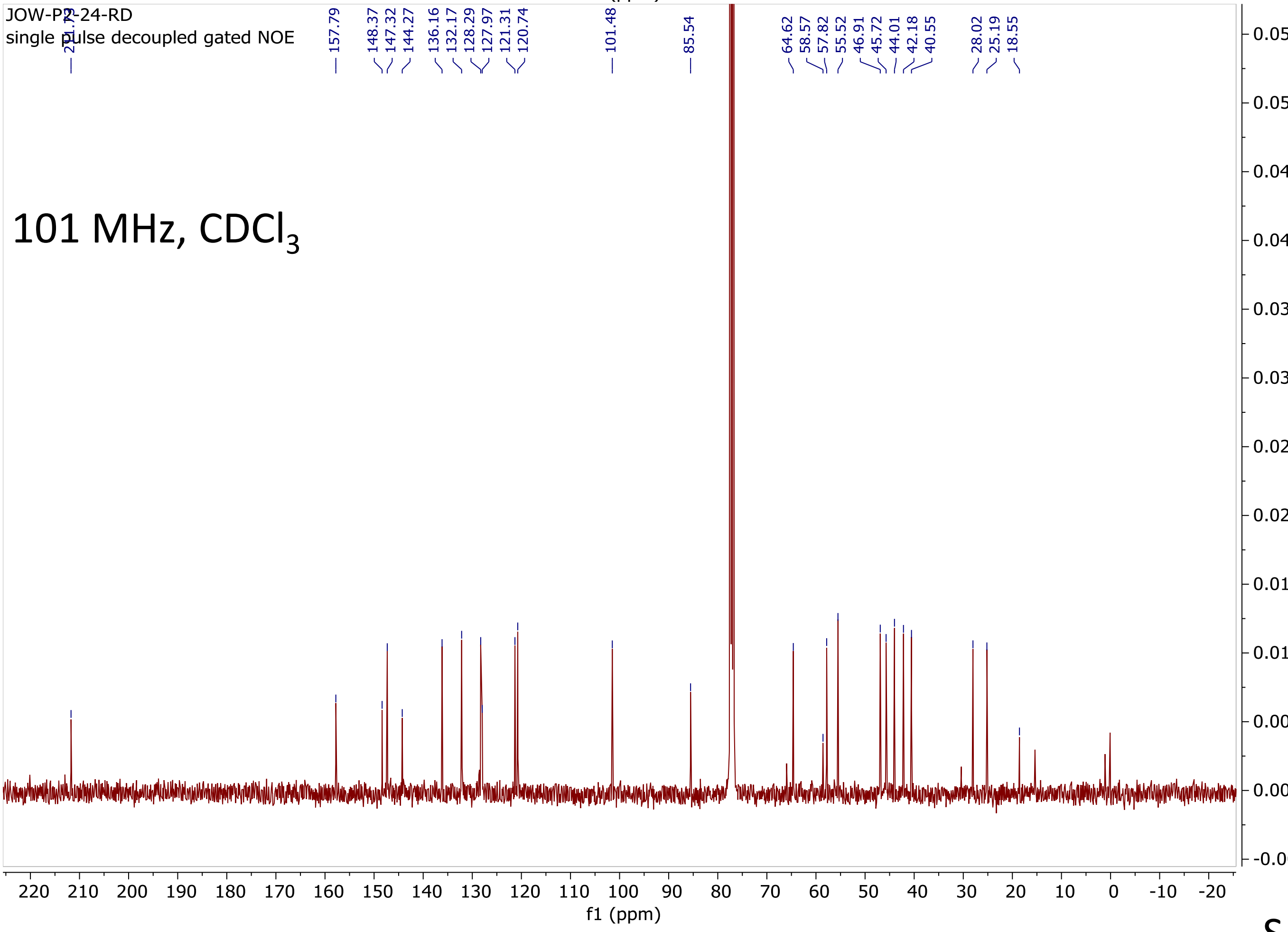

Cascade product 7bb

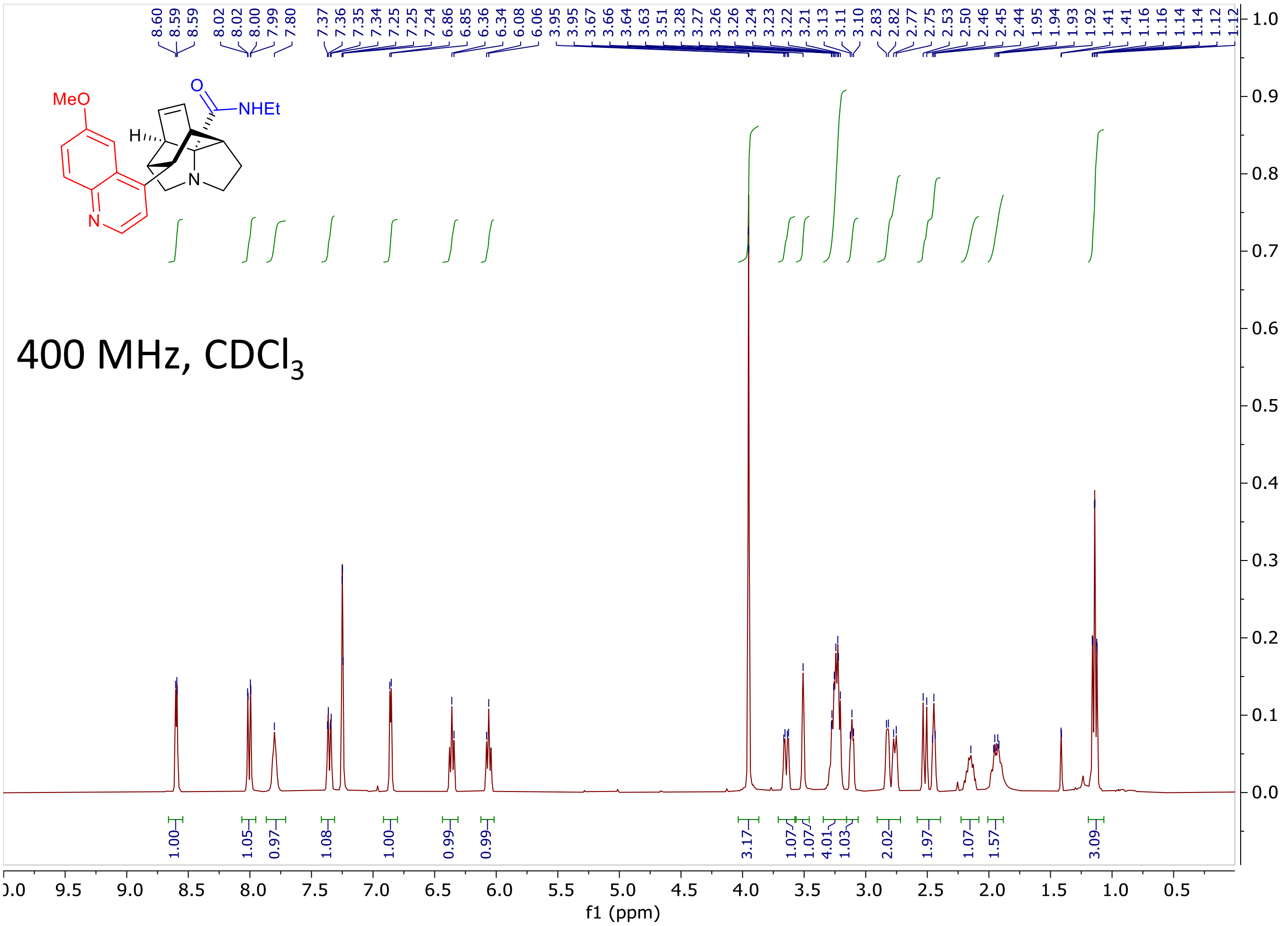

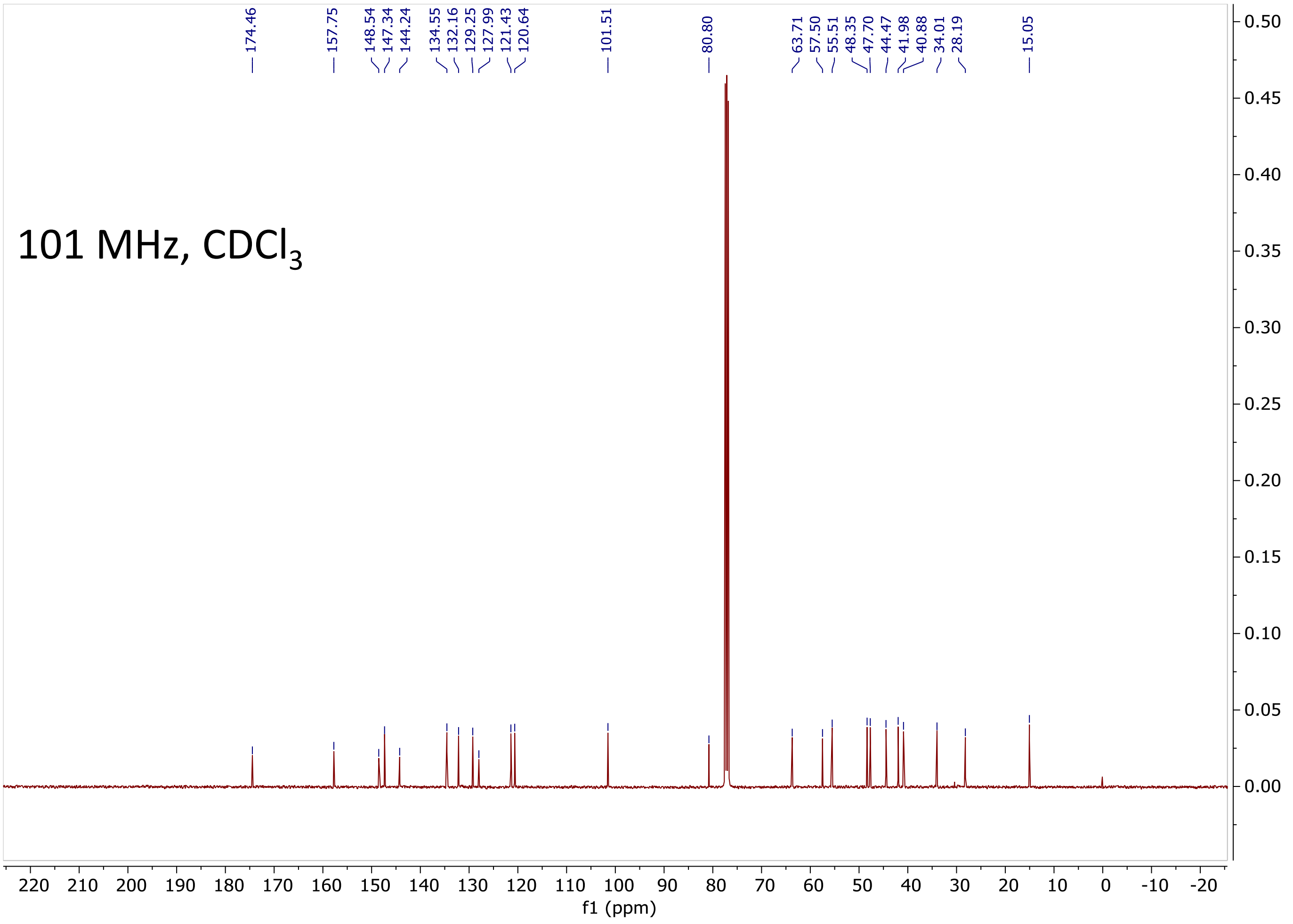

Cascade product 7bc

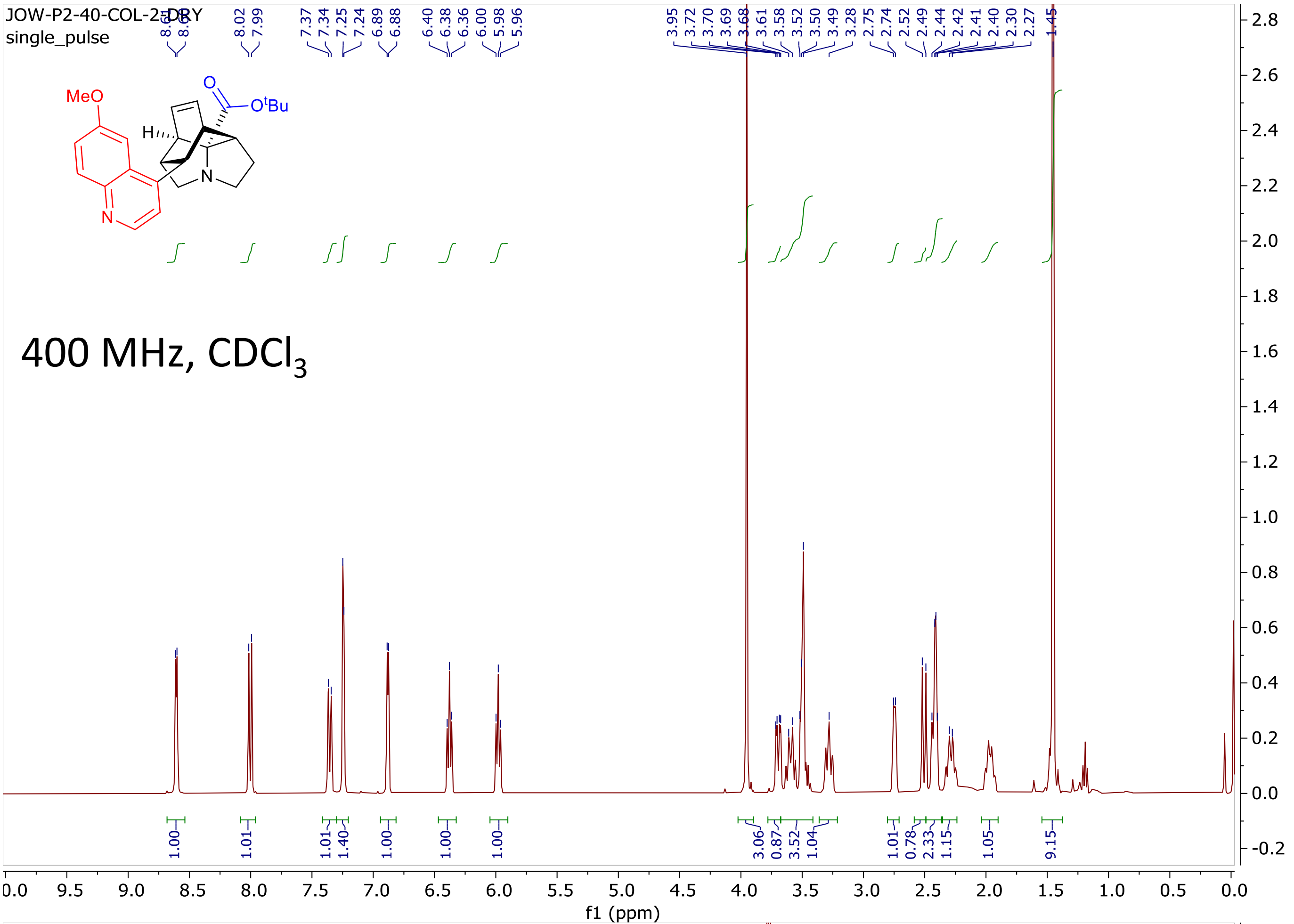

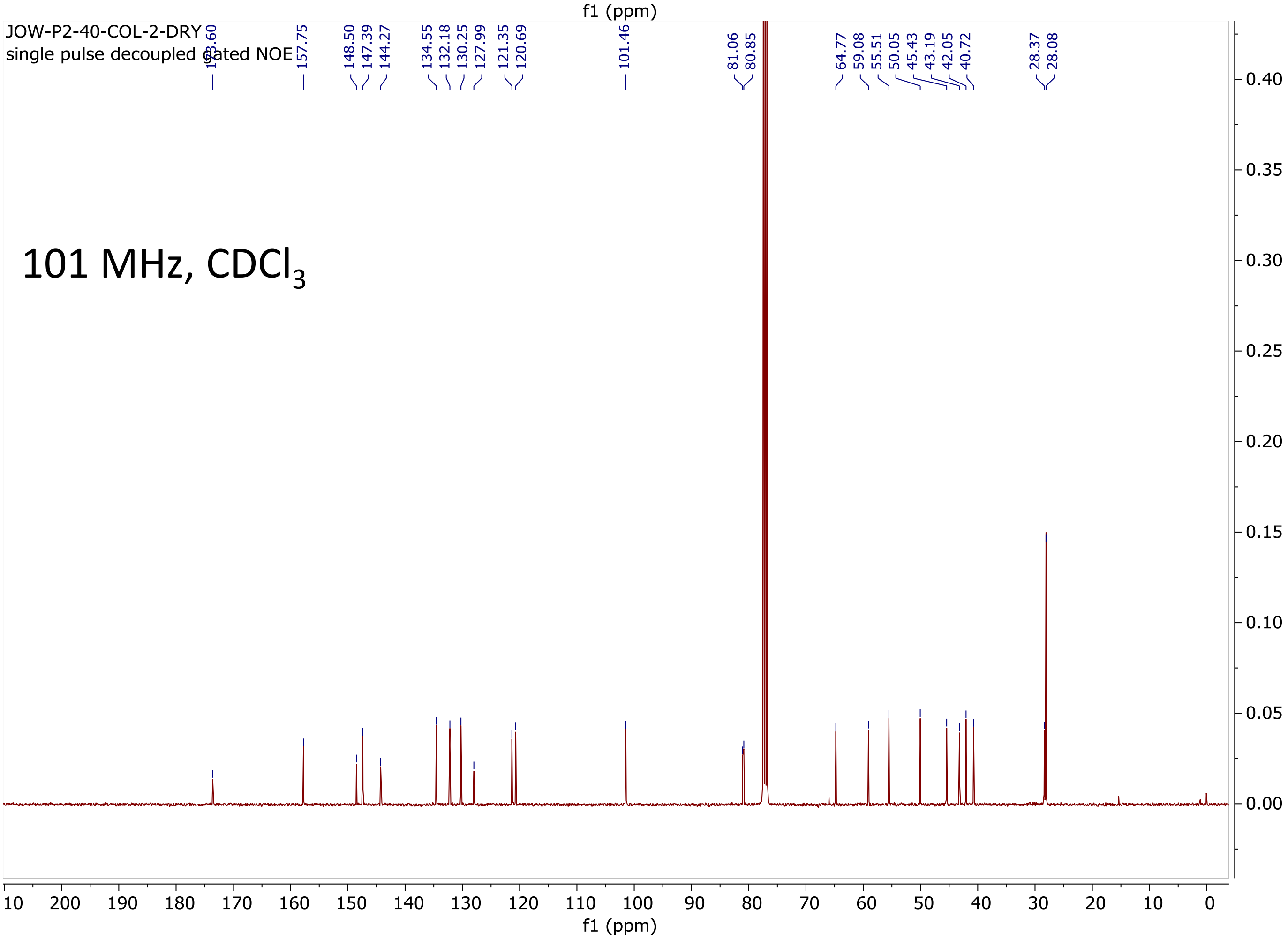

Cascade product 7ca

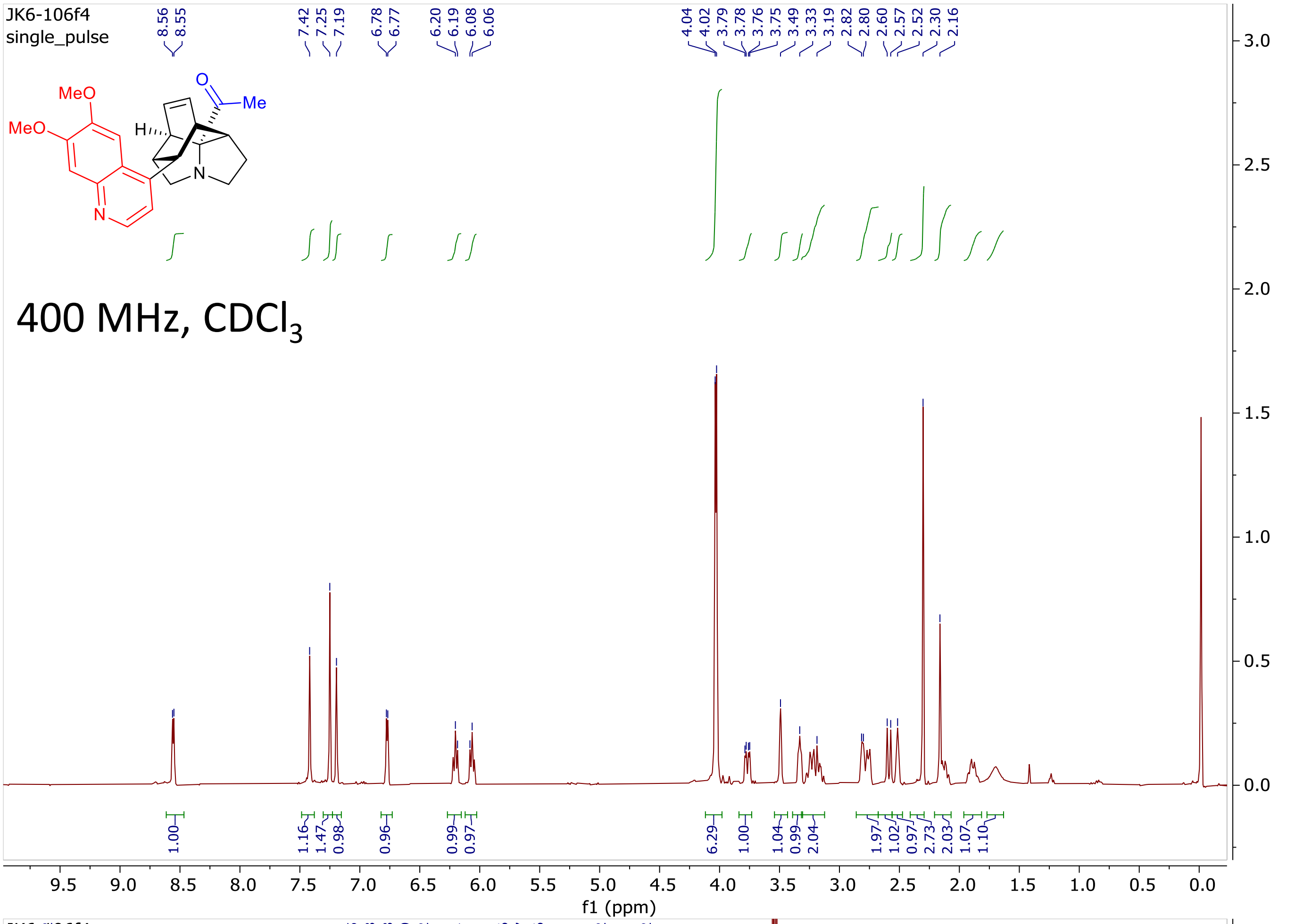

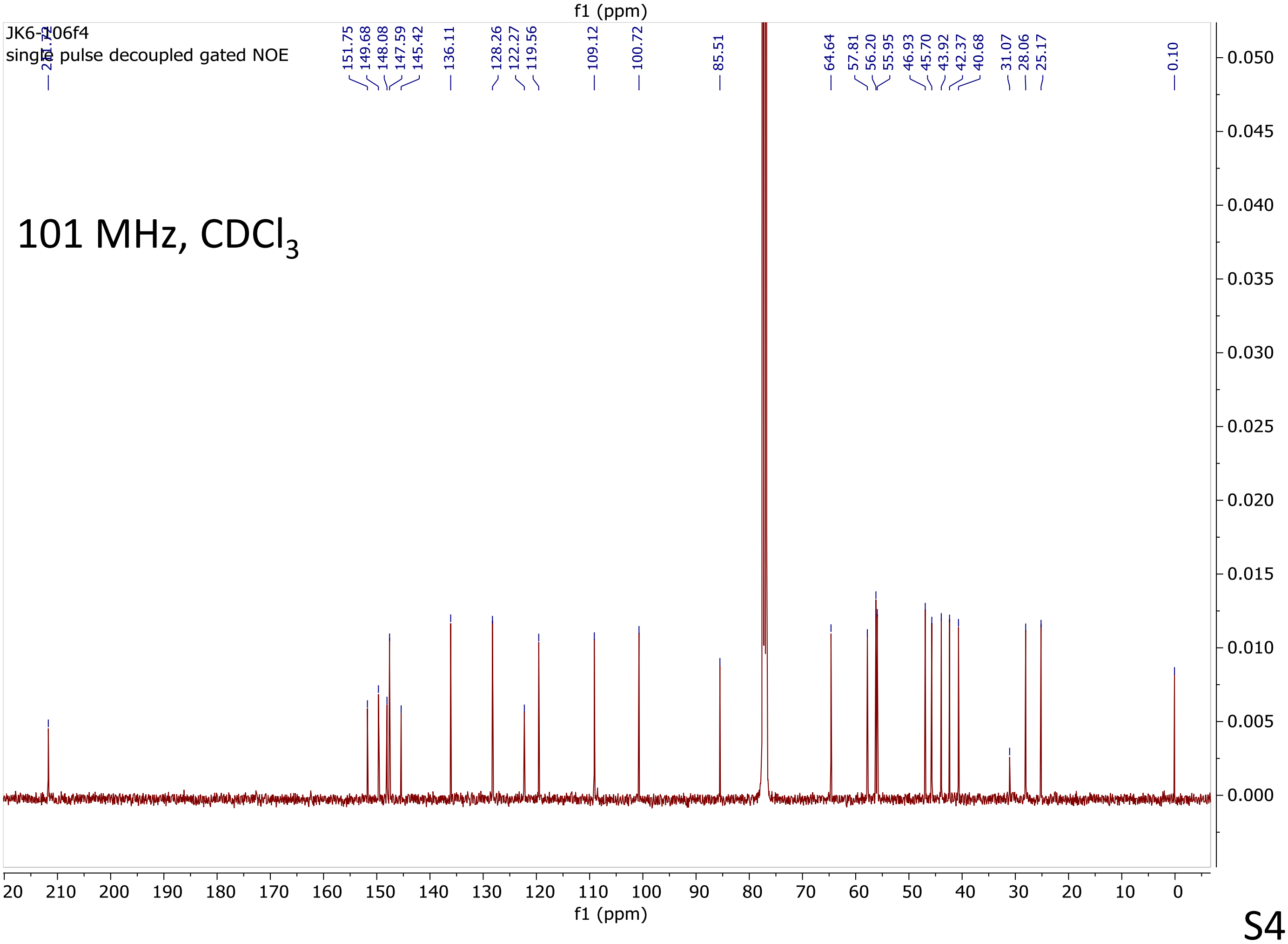

Cascade product 7cb

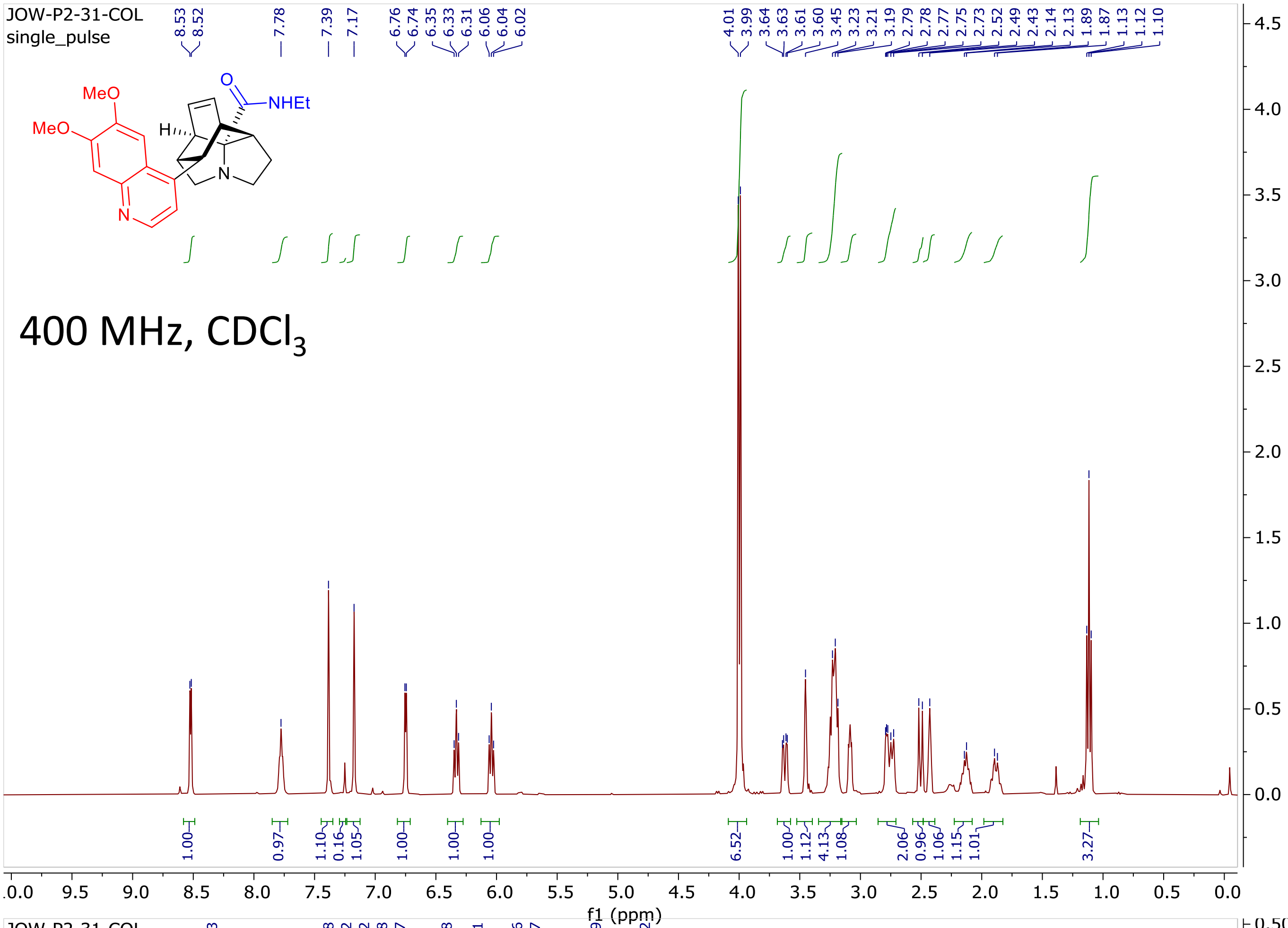

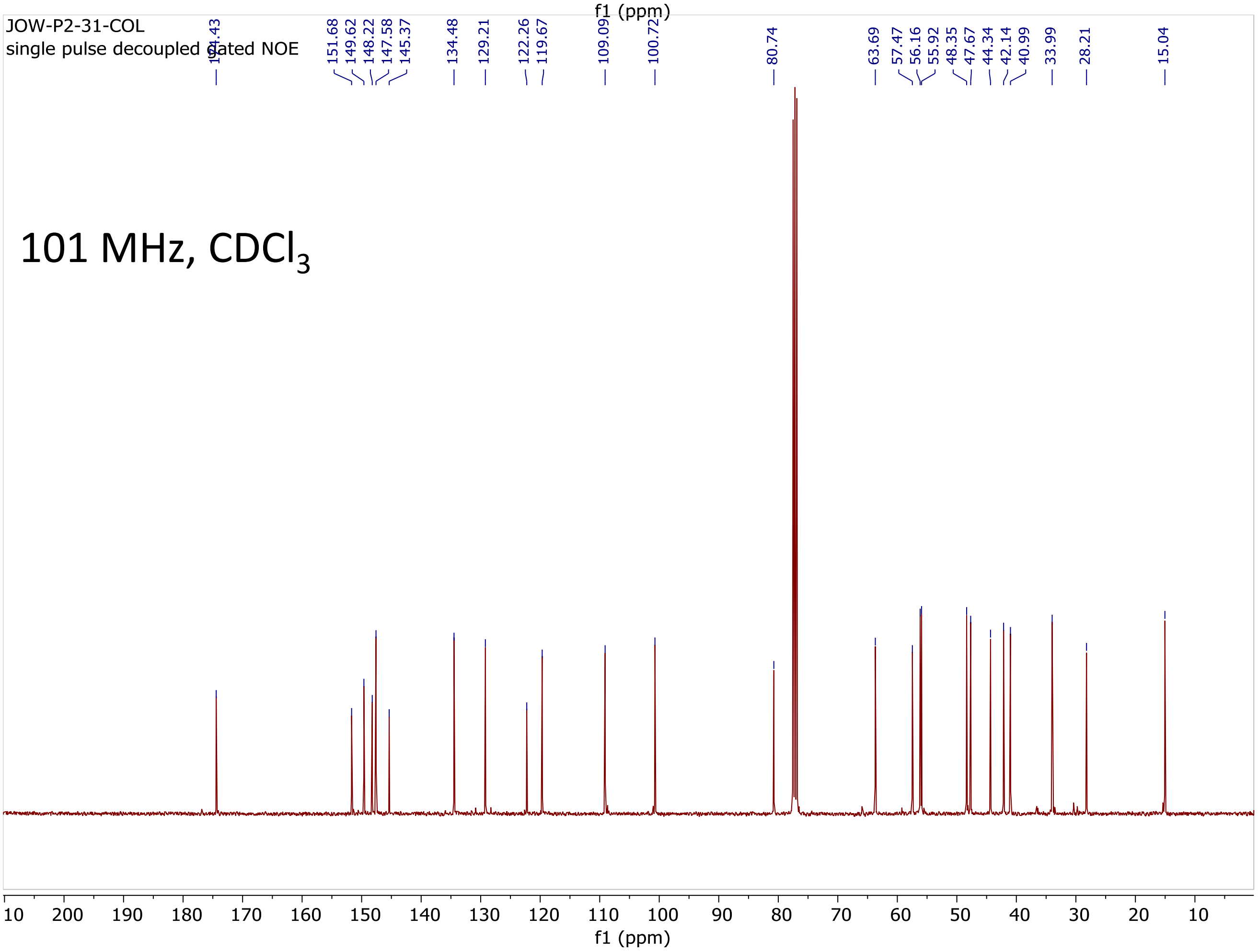

Cascade product 7cc

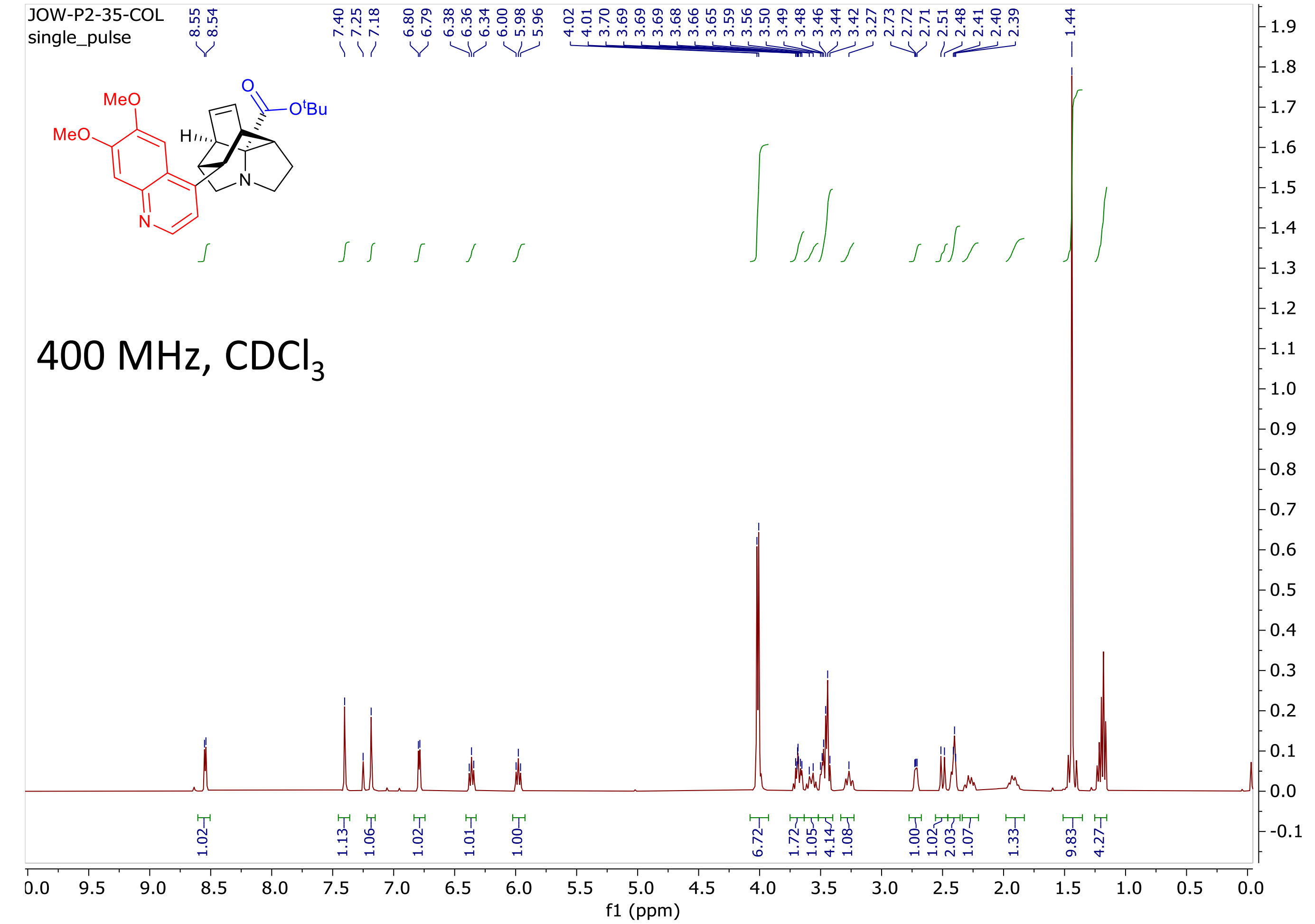

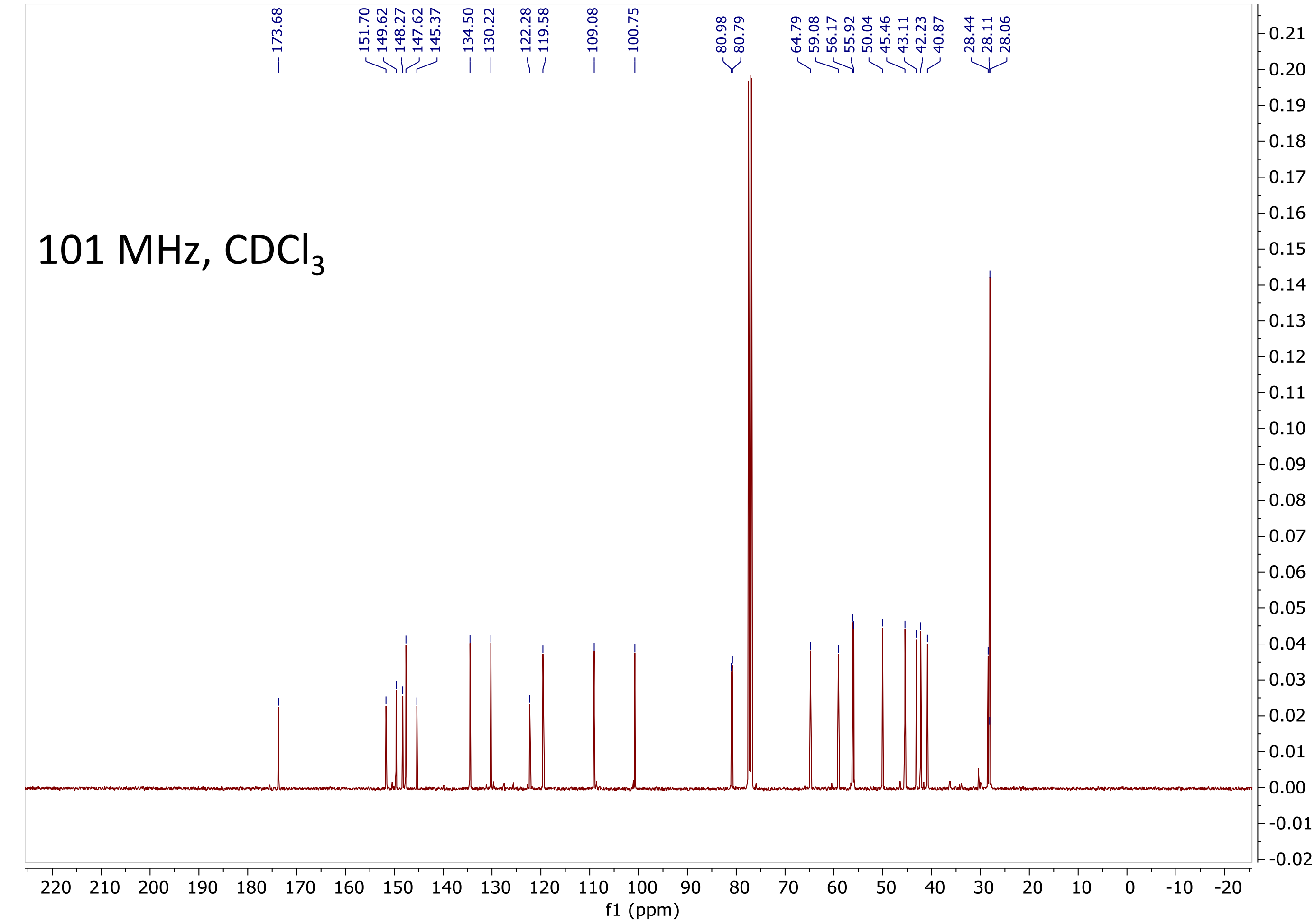

Cascade product 7da

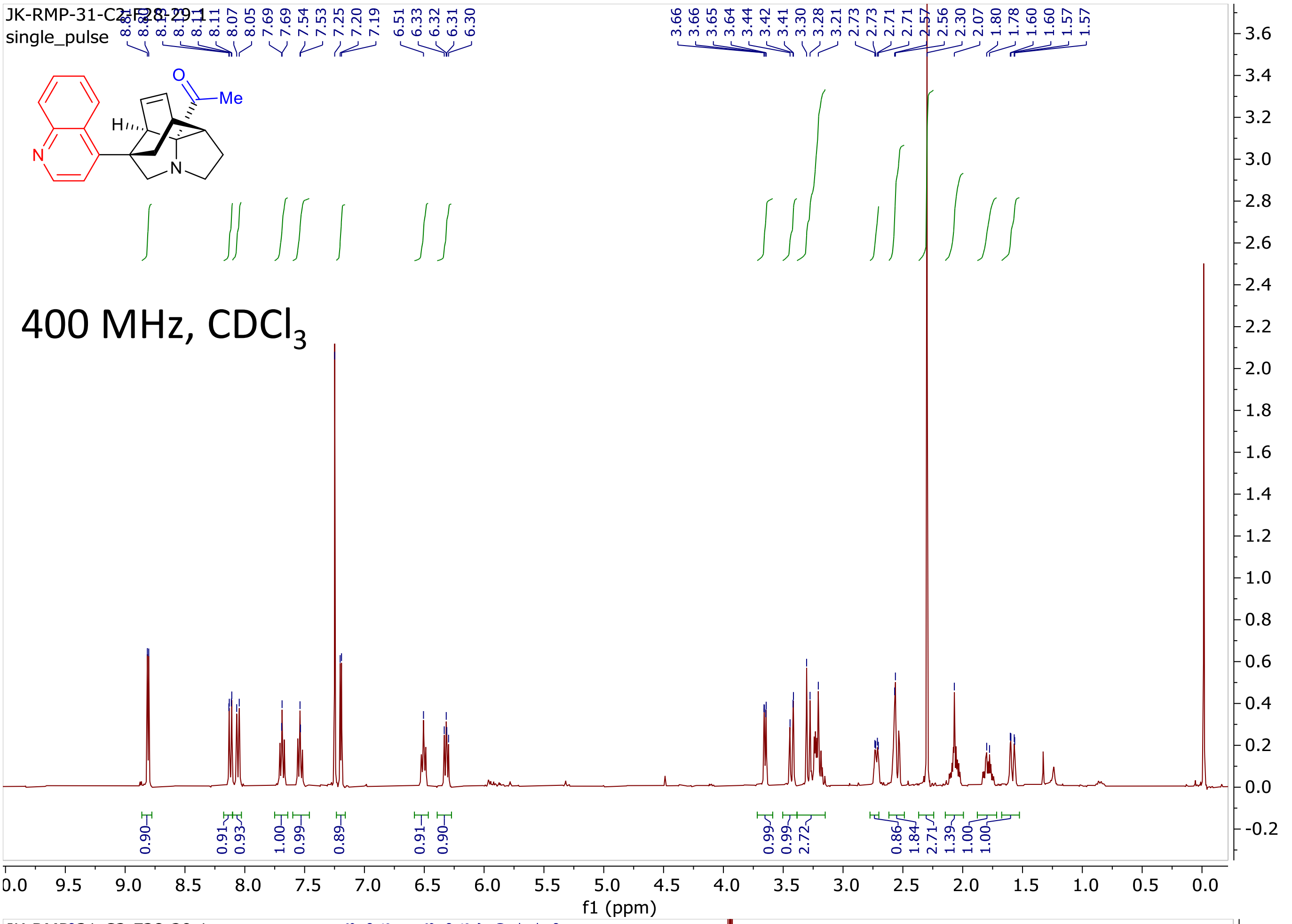

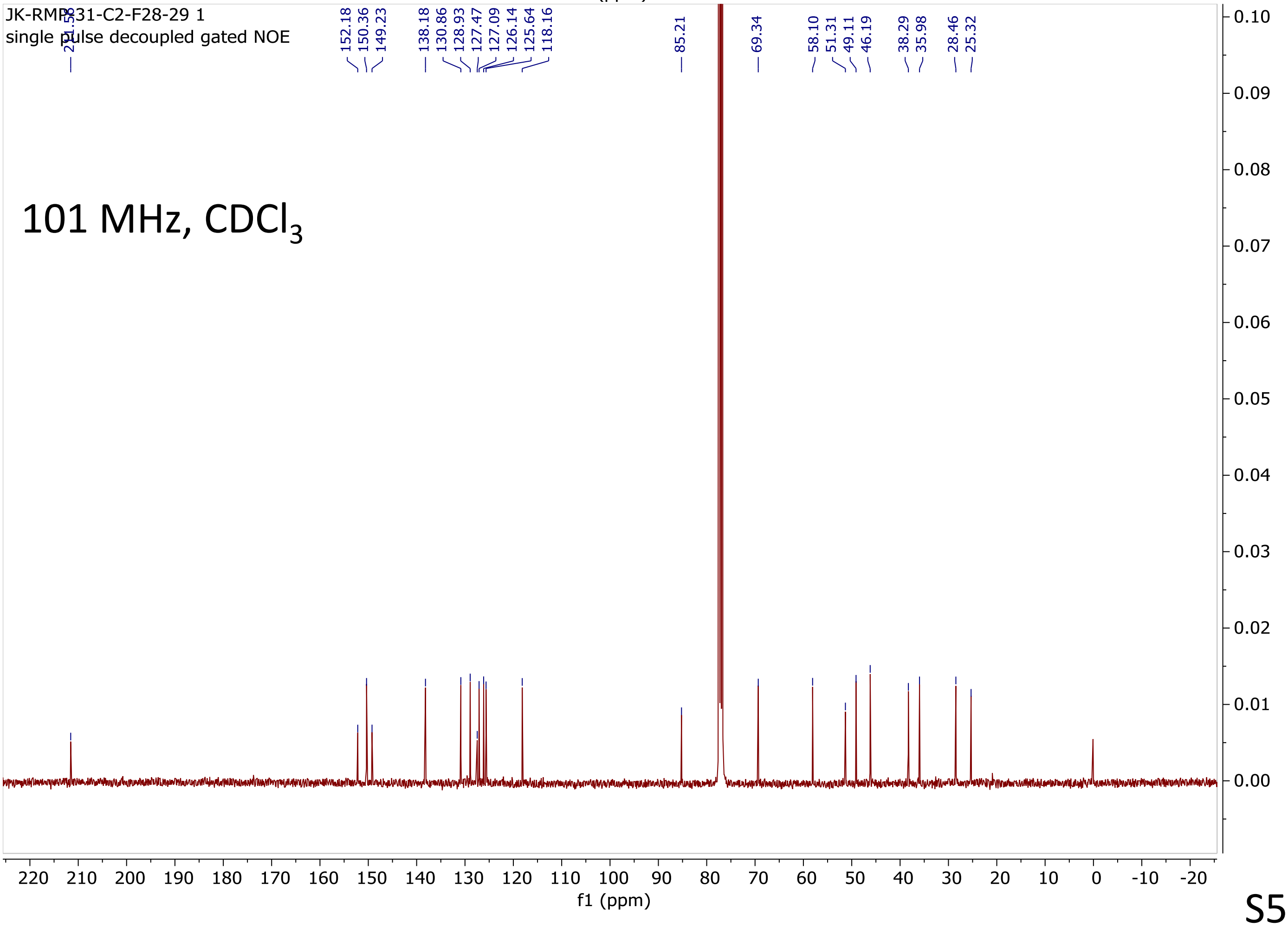

Cascade product 7db

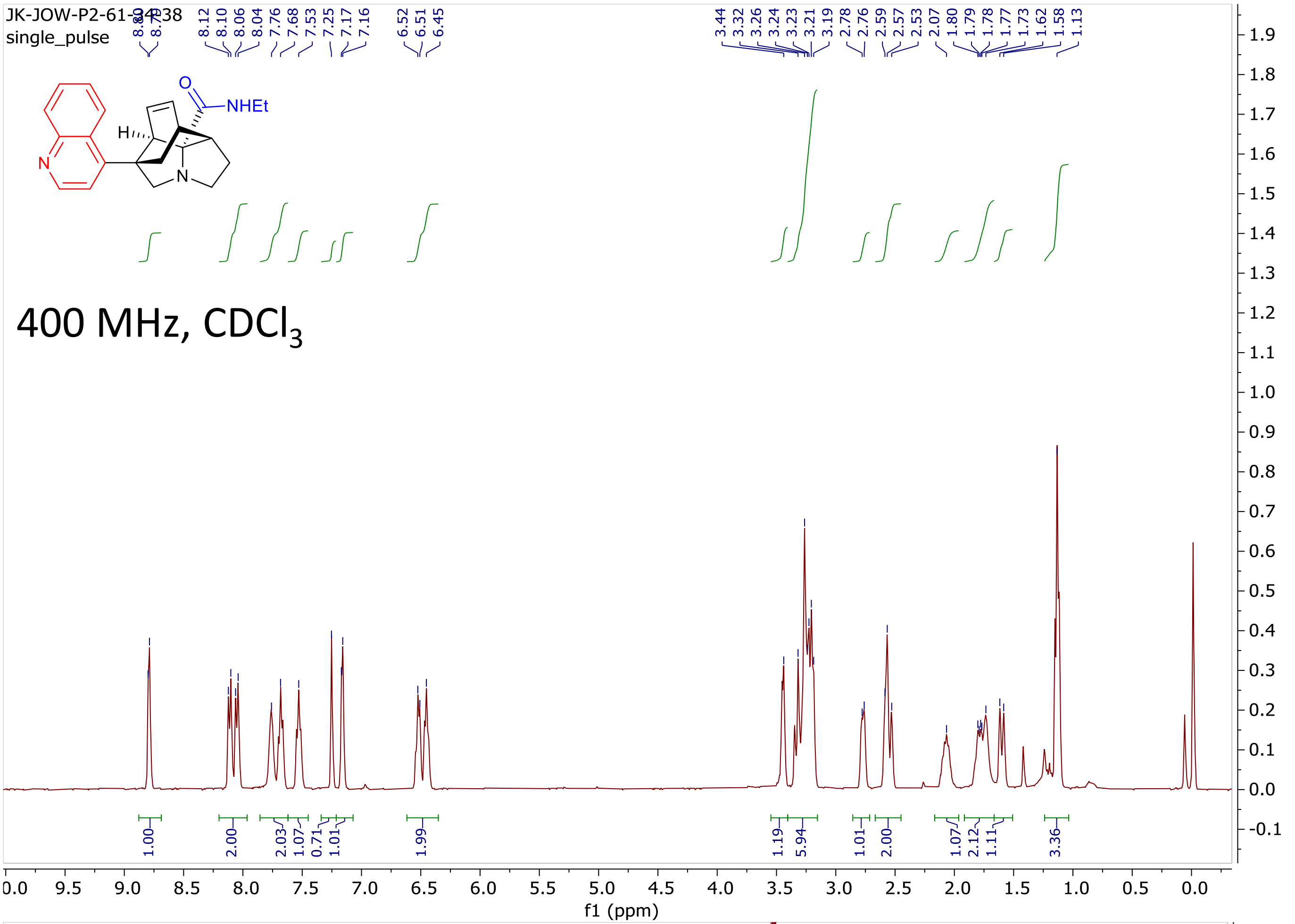

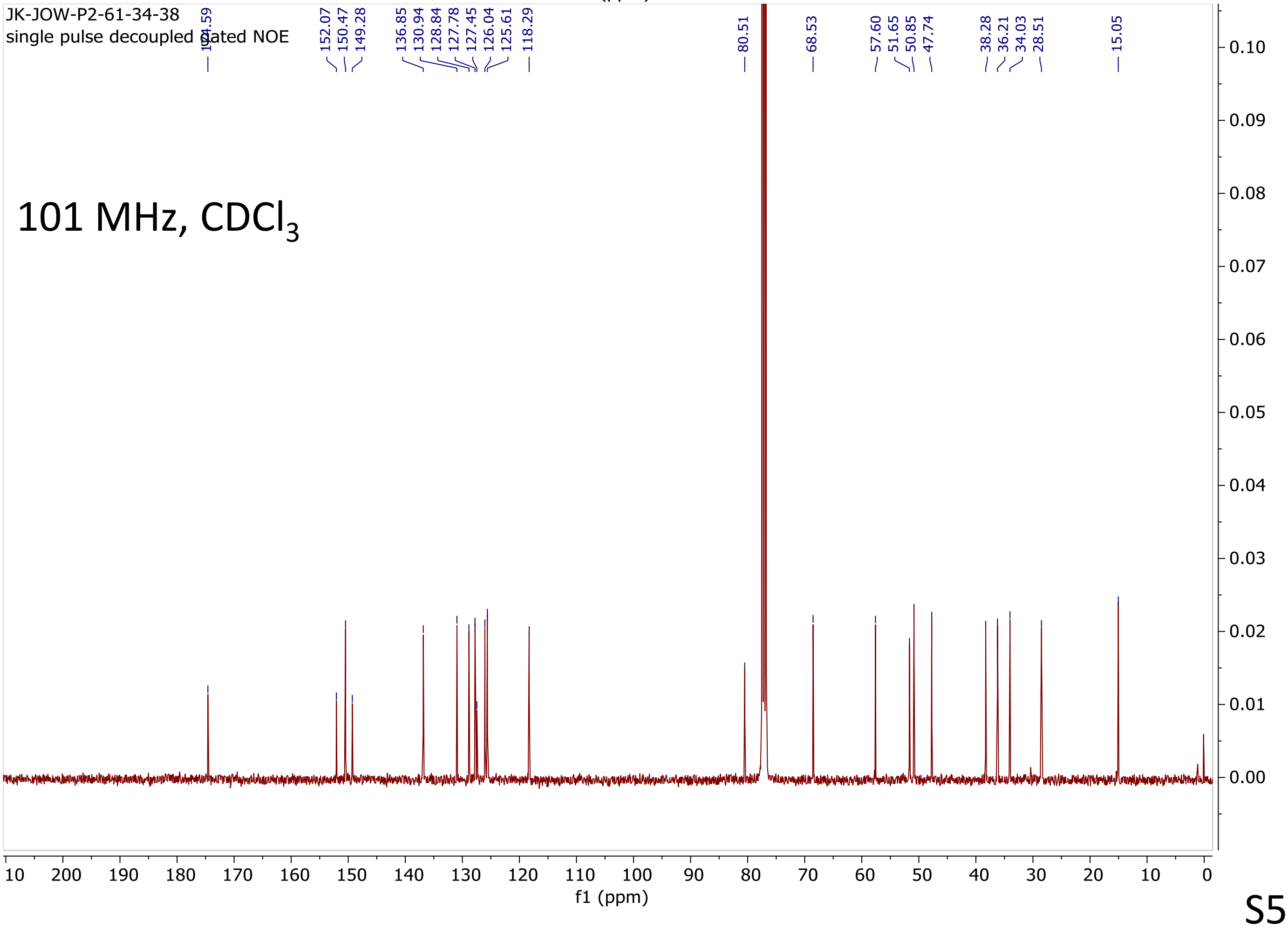

Cascade product 7dc

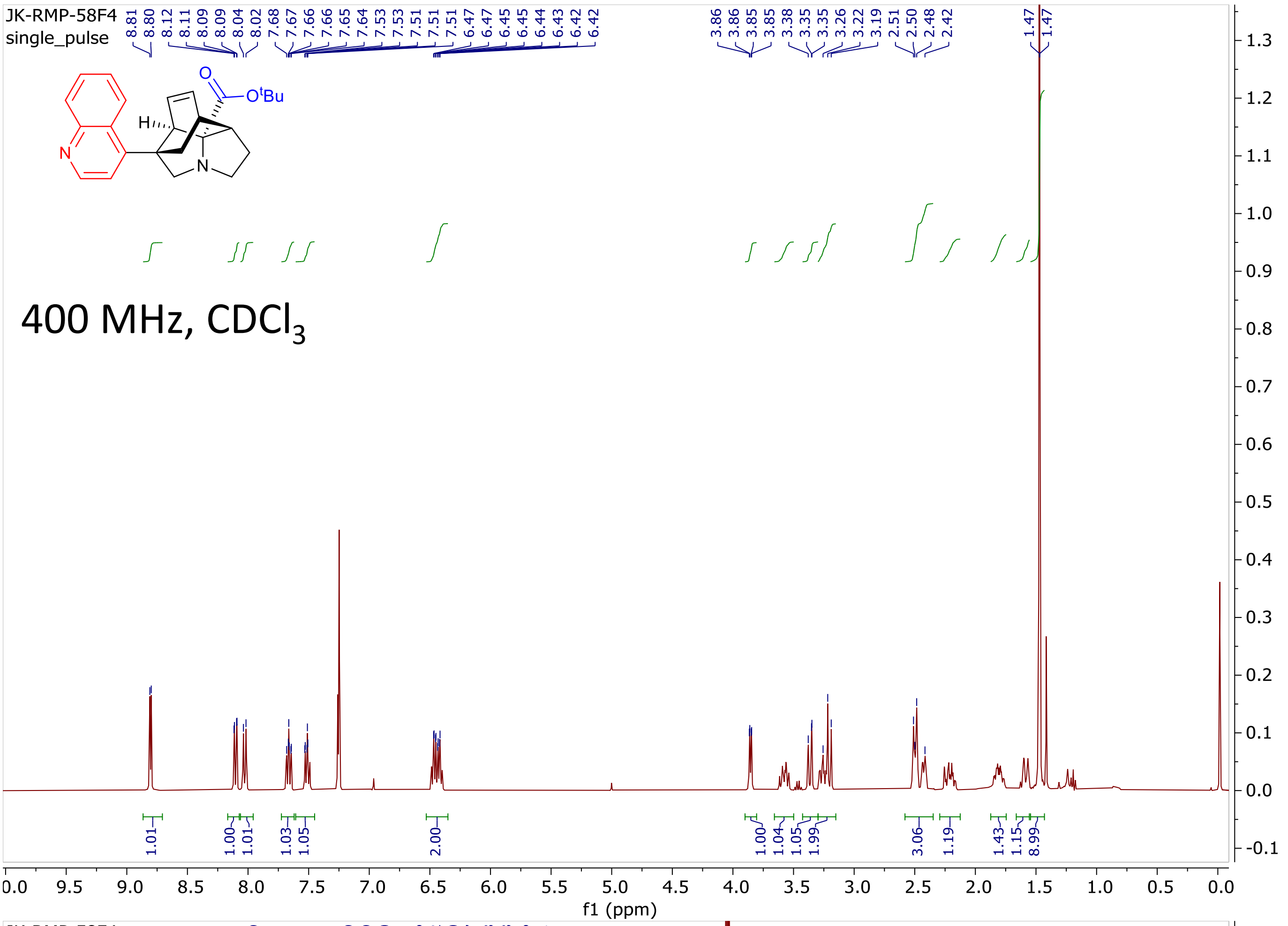

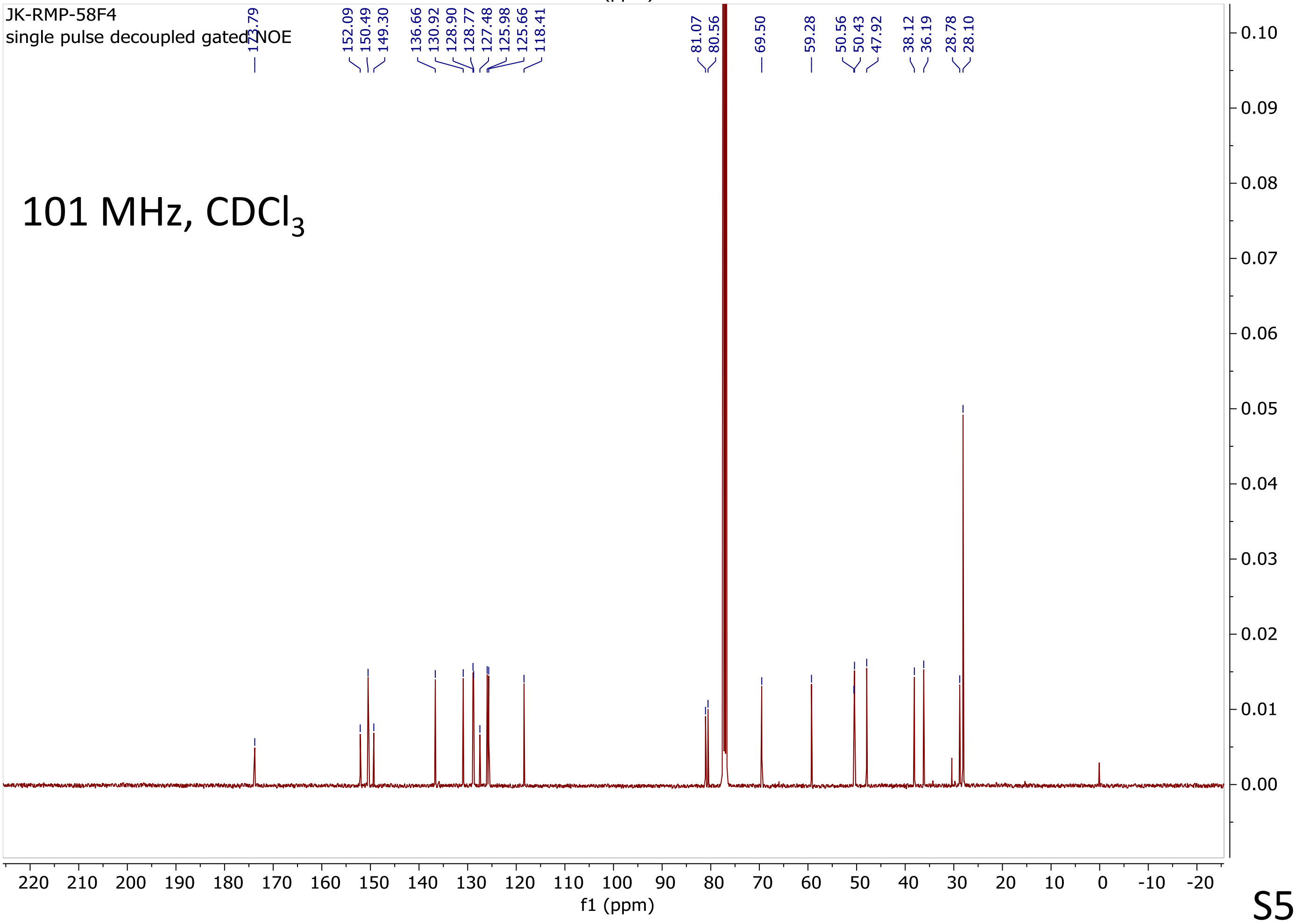

Supplement: Supplementary file 1 — jp4c05220_si_001.pdf [file jp4c05220_si_001.pdf]
